# Supplementary material for: Complete separation of benzene-cyclohexene-cyclohexane mixtures via temperature-dependent molecular sieving by a flexible chain-like coordination polymer
Source: Nat Commun. 2024 Mar 12;15:2240. doi: 10.1038/s41467-024-46556-6 (PMC10933443; doi:10.1038/s41467-024-46556-6)
Supplement: Supplementary file 1 — Supplementary Information [file 41467_2024_46556_MOESM1_ESM.pdf]

Supplementary Information for

Complete separation of benzene-cyclohexene-cyclohexane mixtures *via* temperature-dependent molecular sieving by a flexible chain-like coordination polymer

Feng Xie[1,5], Lihang Chen[2,5], Eder Moisés Cedeño Morales[3], Saif Ullah[4], Yiwen Fu[2], Timo Thonhauser[4], Kui Tan[3]*, Zongbi Bao[2]* and Jing Li[1]*

[1] Department of Chemistry and Chemical Biology, Rutgers University, 123 Bevier Road, Piscataway, New Jersey 08854, USA.

[2] Key Laboratory of Biomass Chemical Engineering of Ministry of Education, College of Chemical and Biological Engineering, Zhejiang University, Hangzhou 310027, P.R. China.

[3] Department of Chemistry, University of North Texas, 1155 Union Cir, Denton, Texas 76203, USA.

[4] Department of Physics and Center for Functional Materials, Wake Forest University, 1834 Wake Forest Road, Winston-Salem, North Carolina 27109, USA.

[5] These authors contributed equally to this work.

***** To whom correspondence should be addressed. Emails: kui.tan@unt.edu (K. Tan); baozb@zju.edu.cn (Z. Bao); jingli@rutgers.edu (J. Li)

This file includes:

Supplementary Figures 1 to 81

Supplementary Tables 1 to 6

Supplementary Note 1

Supplementary References 1 to 26

Supplementary Figures

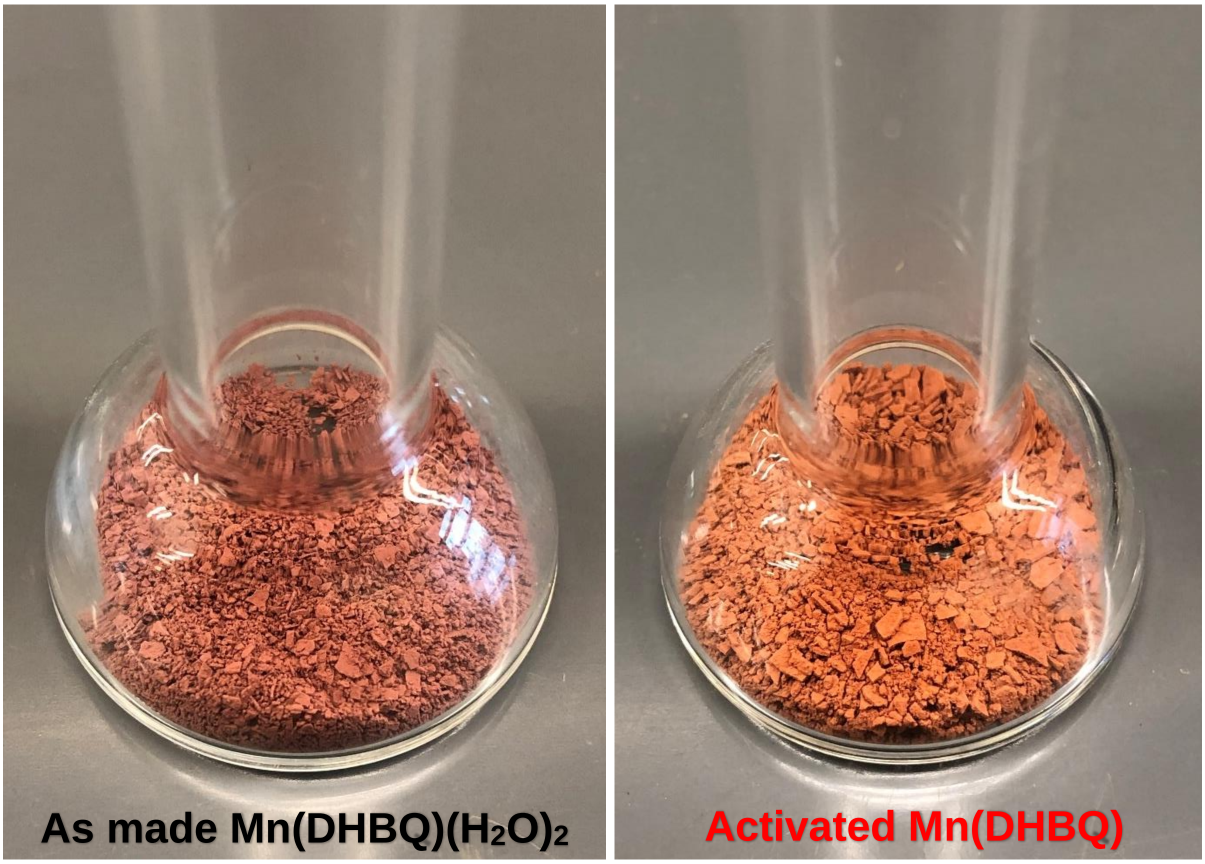

Supplementary Fig. 1. The as-made and activated (dehydrated) samples of Mn-DHBQ.

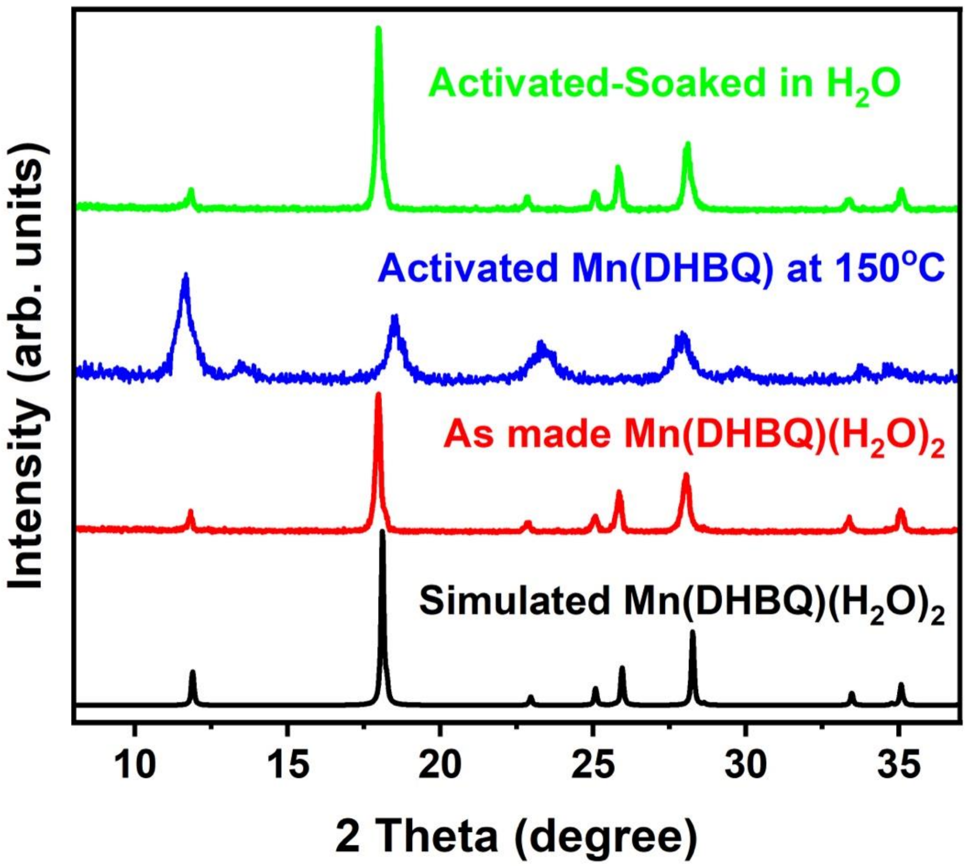

Supplementary Fig. 2. The powder X-ray diffraction (PXRD) patterns of the simulated, as-made, activated, and rehydrated Mn-DHBQ.

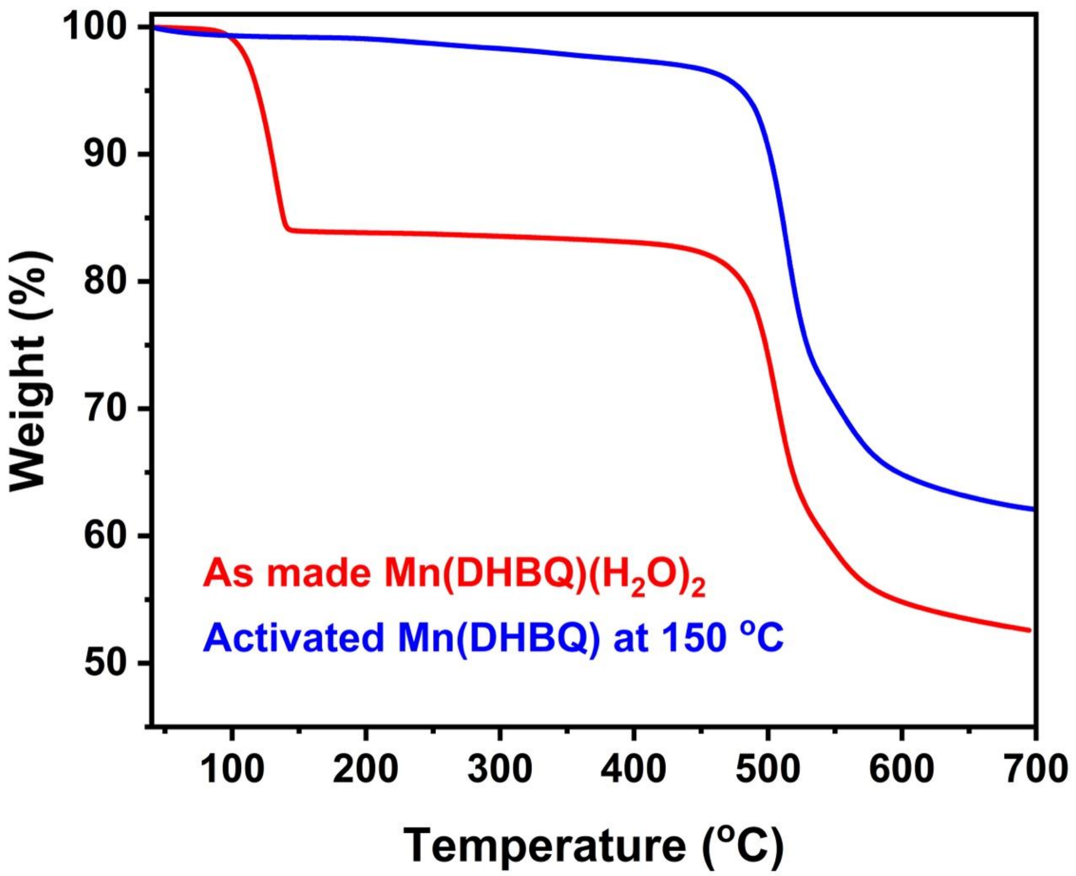

Supplementary Fig. 3. The TGA profiles of the as-made and activated Mn-DHBQ samples collected under nitrogen with a heat rate of 5 °C min^{-1}.

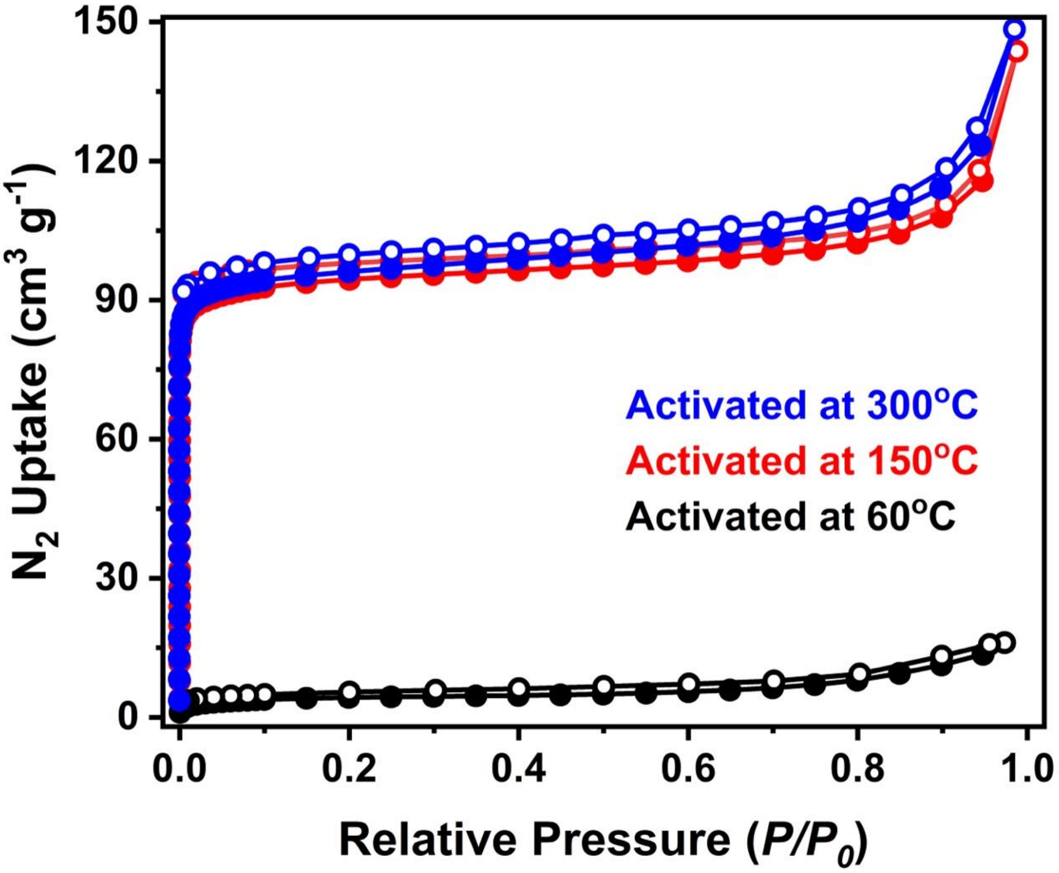

Supplementary Fig. 4. The N_2 sorption isotherms (77 K) on Mn-DHBQ samples activated at different temperatures. Water molecules remain coordinated to Mn at 60 °C.

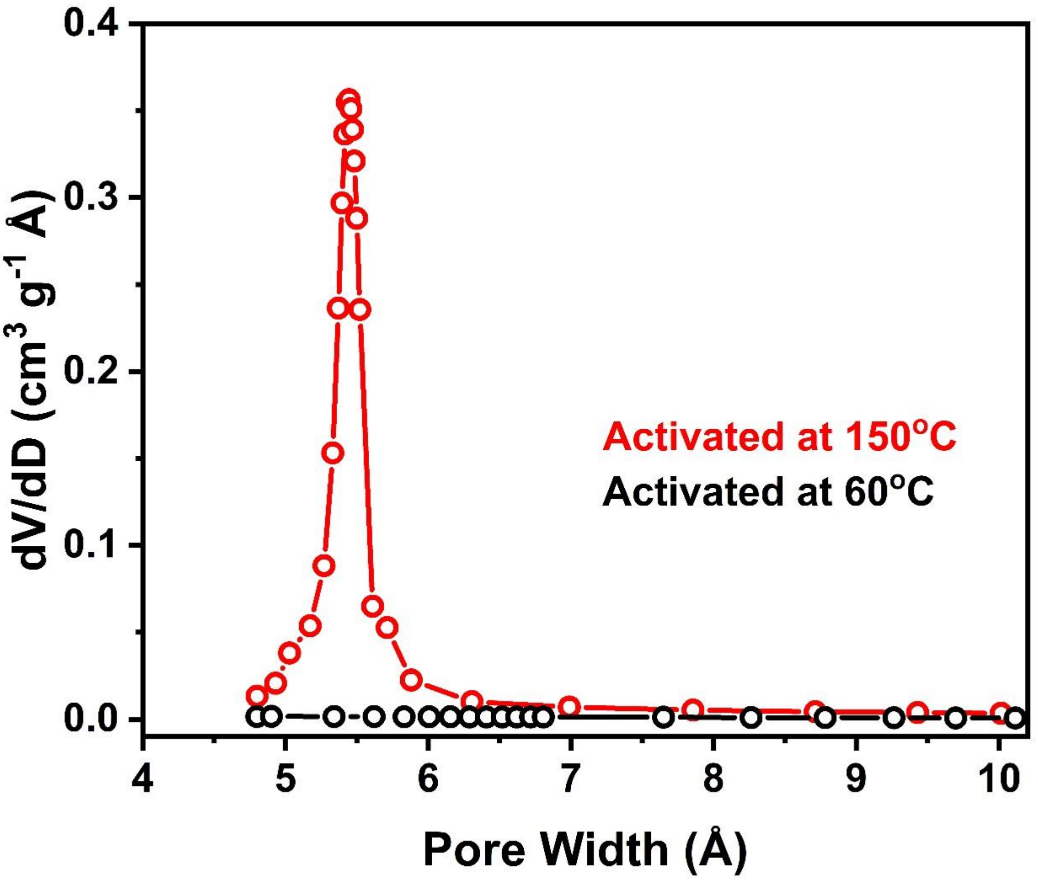

Supplementary Fig. 5. The estimated pore size distribution (H-K model) of Mn-DHBQ samples activated at different temperatures. Water molecules remain coordinated to Mn at 60 °C.

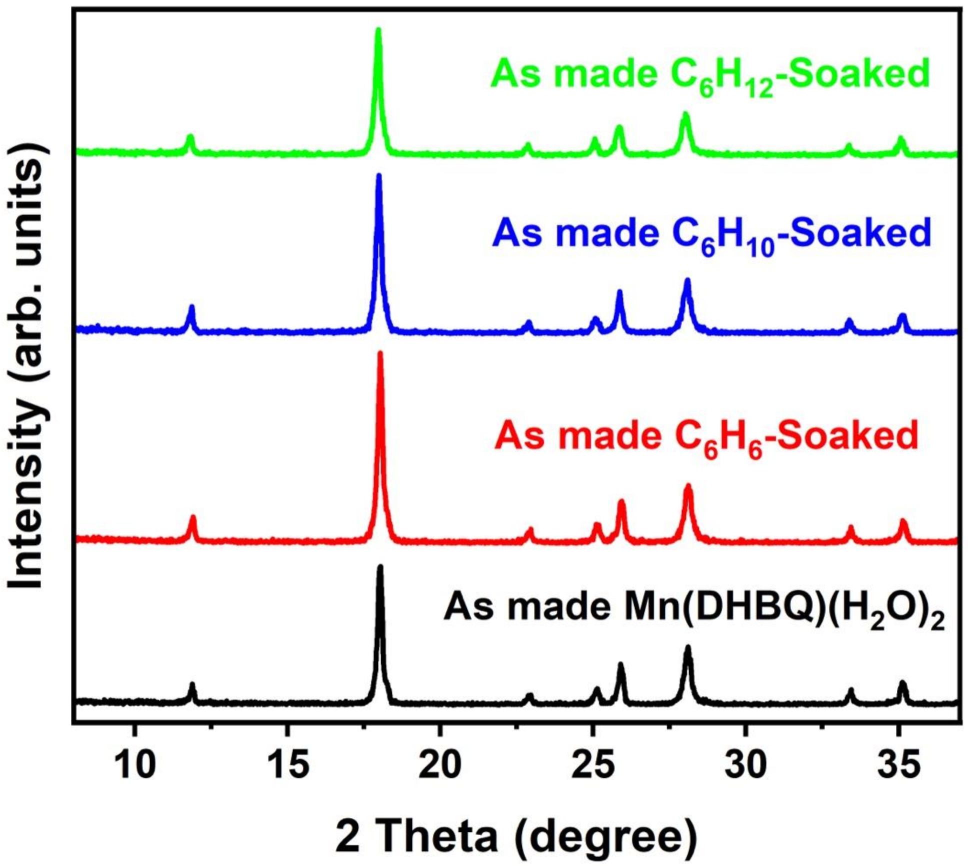

Supplementary Fig. 6. The PXRD patterns of the as-made and C6 cyclic hydrocarbon-loaded Mn-DHBQ samples.

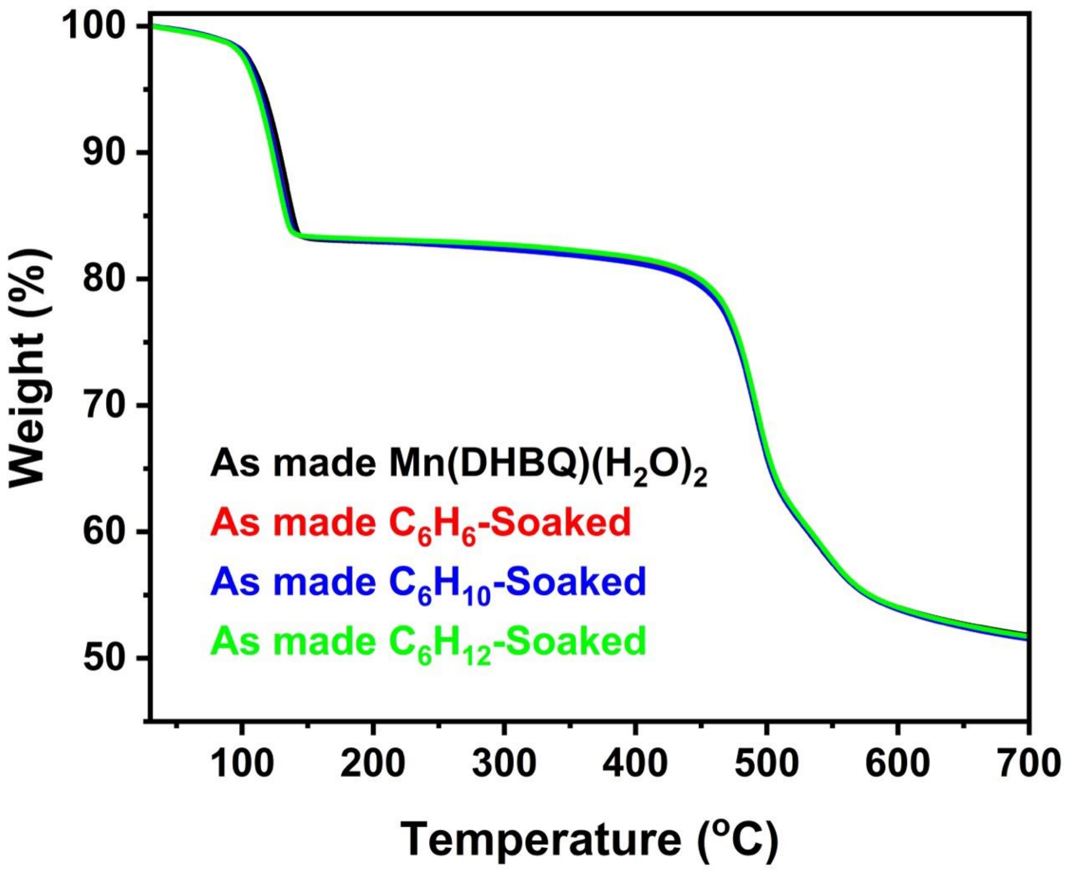

Supplementary Fig. 7. The thermogravimetric curves of fully hydrocarbon-loaded on as-made samples for C_6H_6@Mn(DHBQ)(H_2O)$_2$, C_6H_{10}@ Mn(DHBQ)(H_2O)$_2$, and C_6H_{12}@ Mn(DHBQ)(H_2O)$_2$ under nitrogen flowing environment with a heat rate of 5 °C min^{-1}.

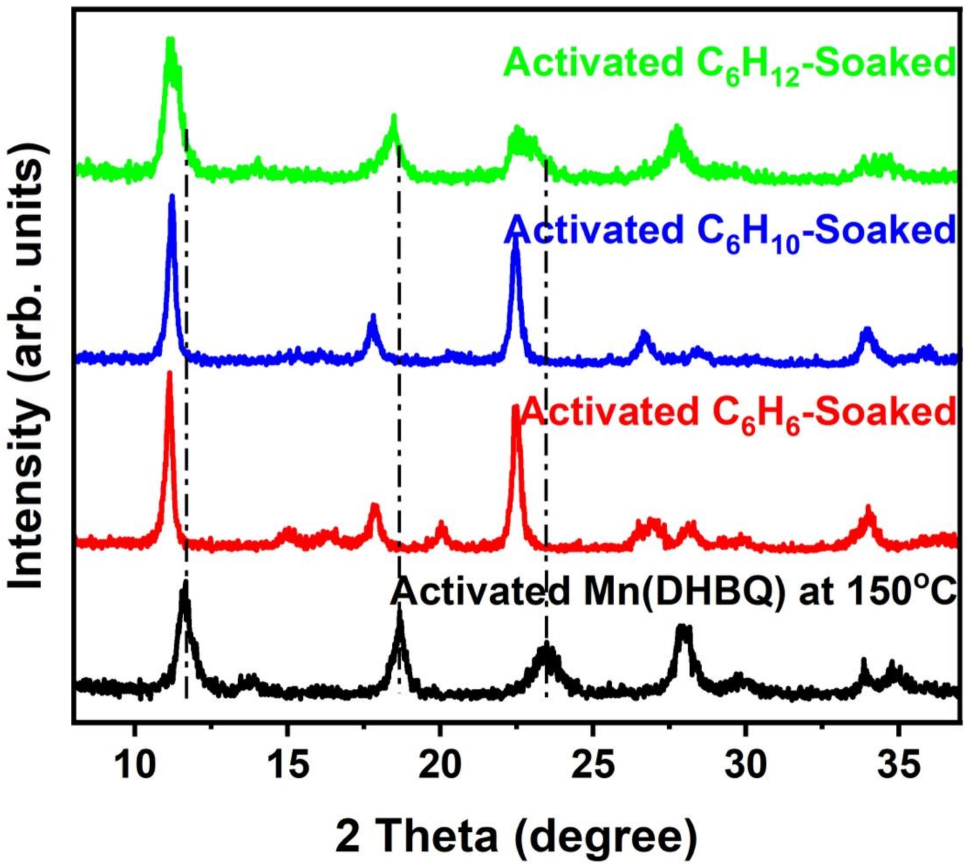

Supplementary Fig. 8. The PXRD patterns of the activated Mn-DHBQ samples after soaking in the C6 cyclic hydrocarbon liquids at room temperature.

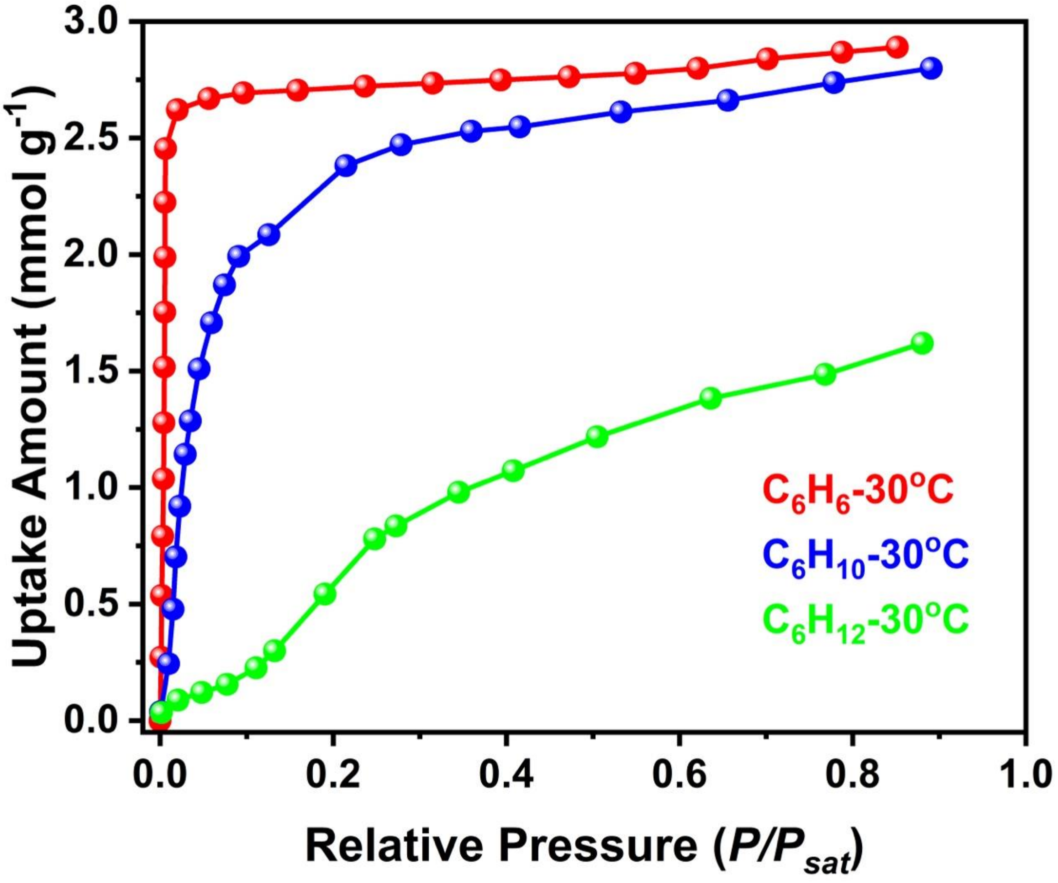

Supplementary Fig. 9. Single-component static adsorption isotherms of benzene (C_6H_6), cyclohexene (C_6H_{10}), and cyclohexane (C_6H_{12}) vapors on Mn-DHBQ sample at 30 °C.

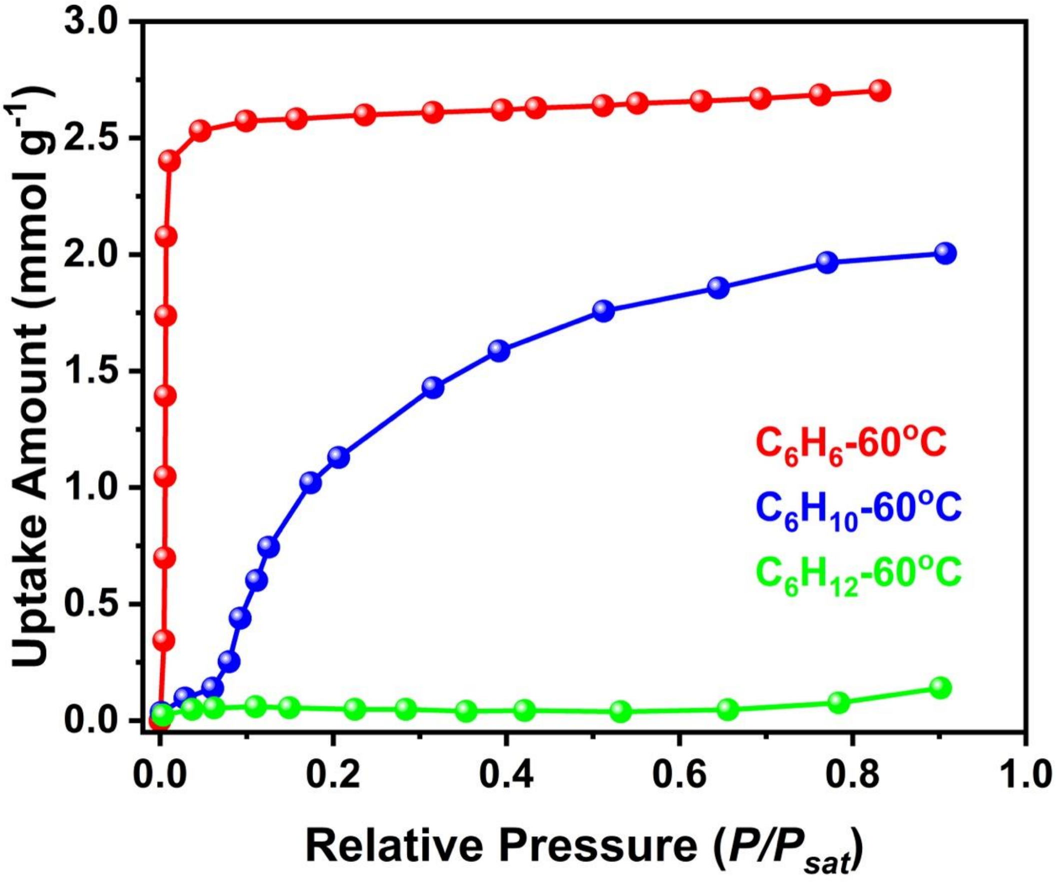

Supplementary Fig. 10. Single-component static adsorption isotherms of benzene (C_6H_6), cyclohexene (C_6H_{10}), and cyclohexane (C_6H_{12}) vapors on Mn-DHBQ sample at 60 °C.

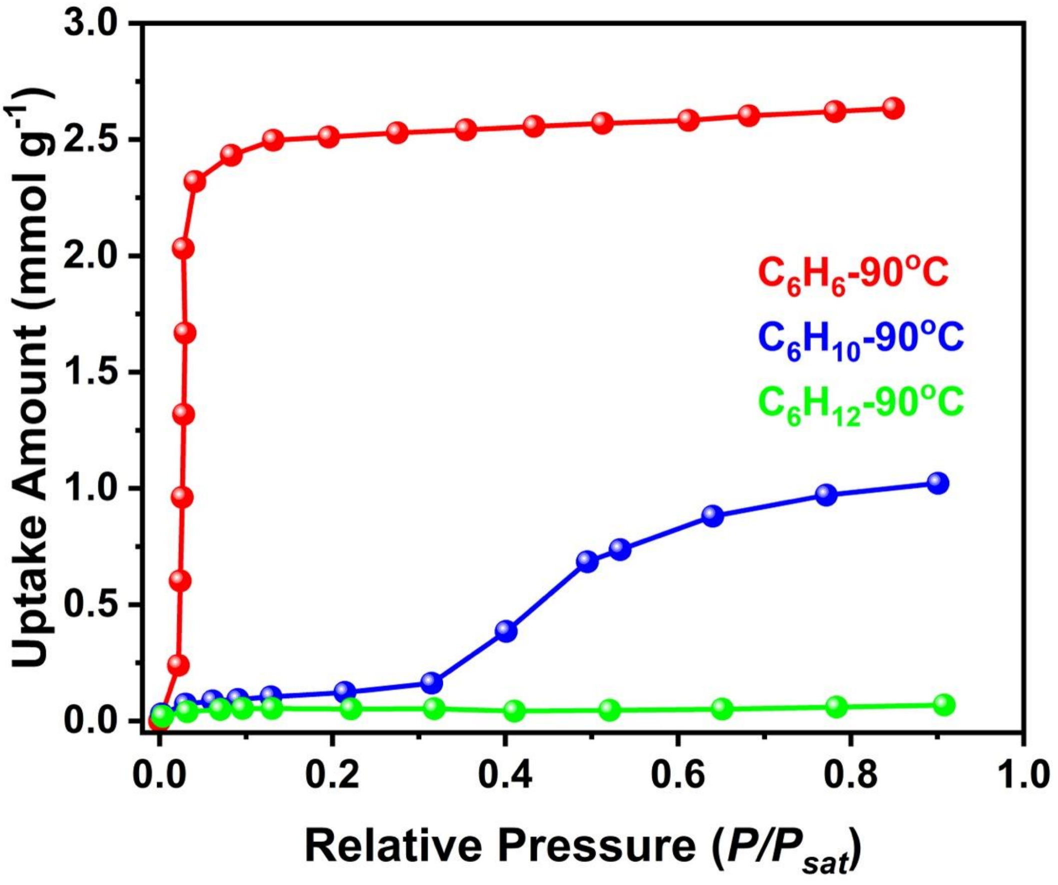

Supplementary Fig. 11. Single-component static adsorption isotherms of benzene (C_6H_6), cyclohexene (C_6H_{10}), and cyclohexane (C_6H_{12}) vapors on Mn-DHBQ sample at 90 °C.

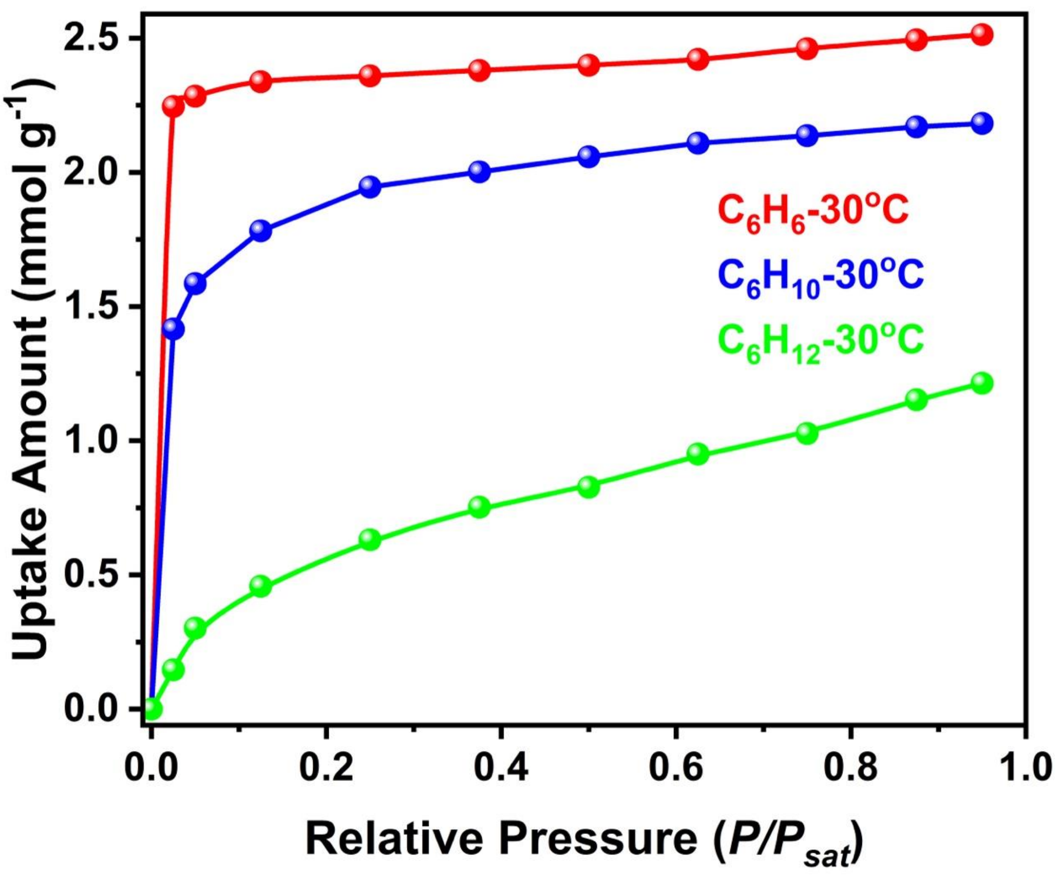

Supplementary Fig. 12. Dynamic adsorption isotherms of benzene (C_6H_6), cyclohexene (C_6H_{10}), and cyclohexane (C_6H_{12}) vapors on Mn-DHBQ sample at 30 °C.

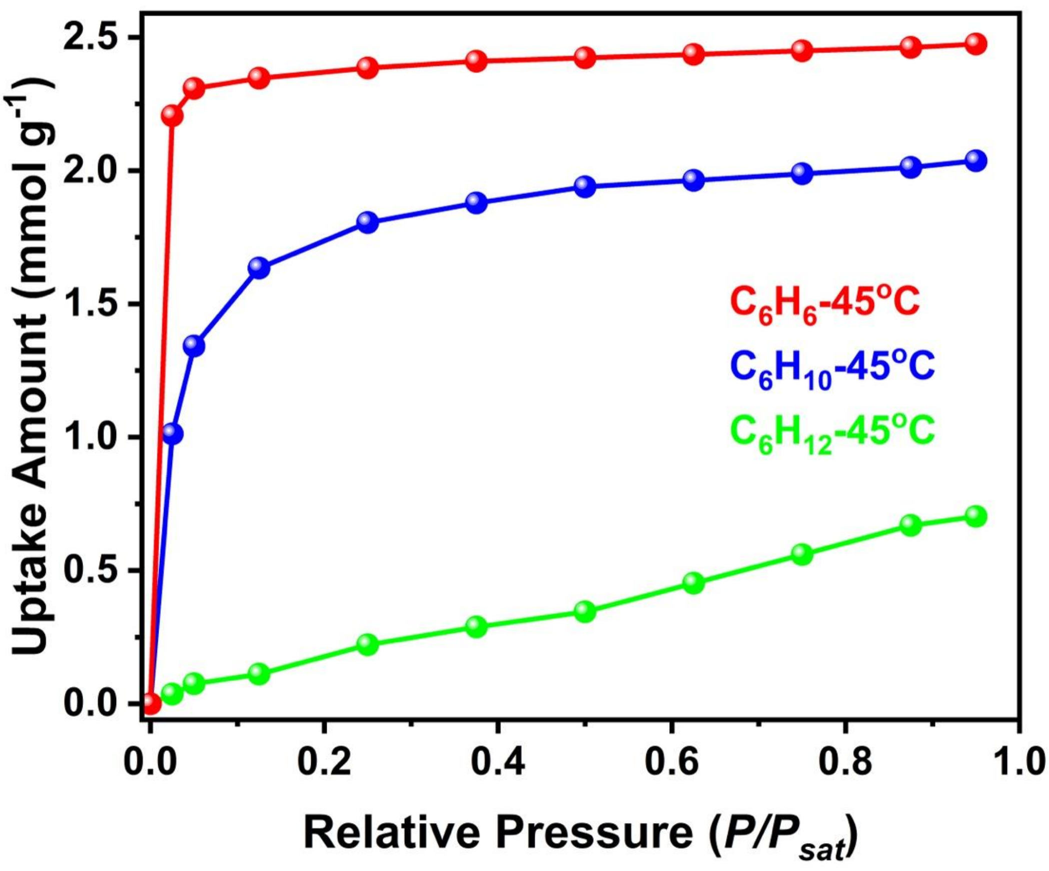

Supplementary Fig. 13. Dynamic adsorption isotherms of benzene (C_6H_6), cyclohexene (C_6H_{10}), and cyclohexane (C_6H_{12}) vapors on Mn-DHBQ sample at 45 °C.

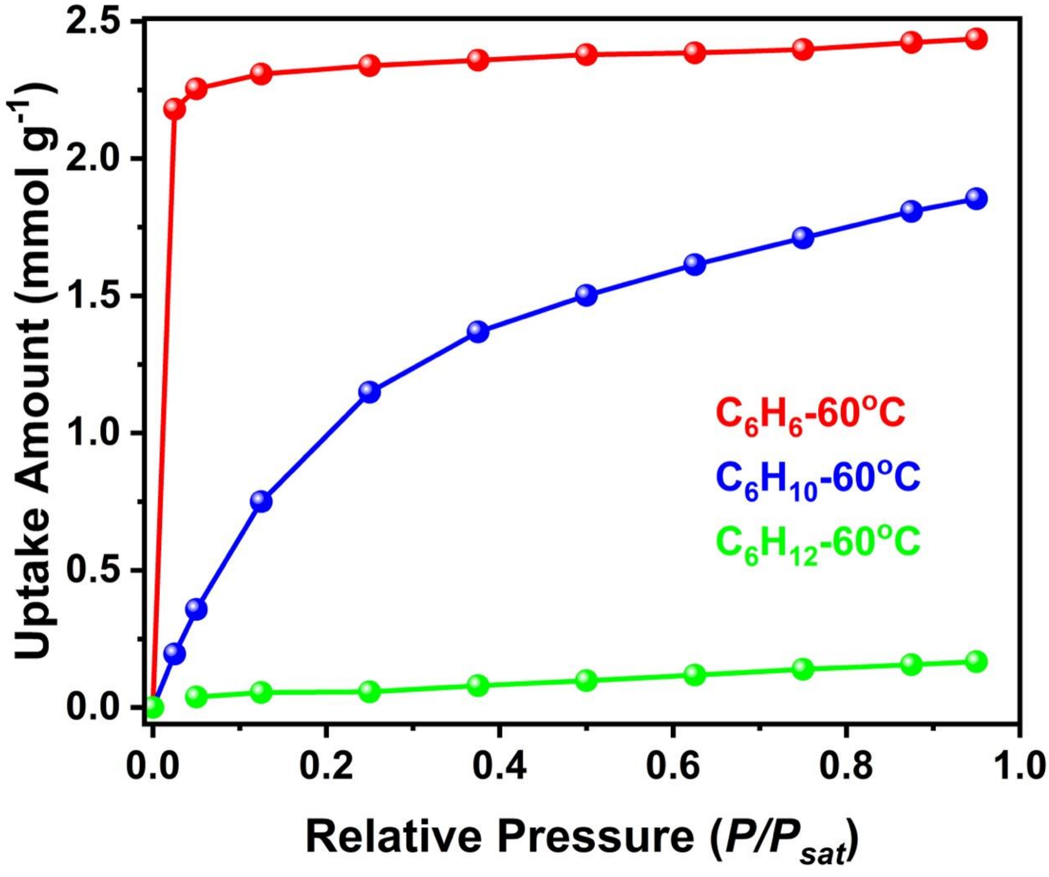

Supplementary Fig. 14. Dynamic adsorption isotherms of benzene (C_6H_6), cyclohexene (C_6H_{10}), and cyclohexane (C_6H_{12}) vapors on Mn-DHBQ sample at 60 °C.

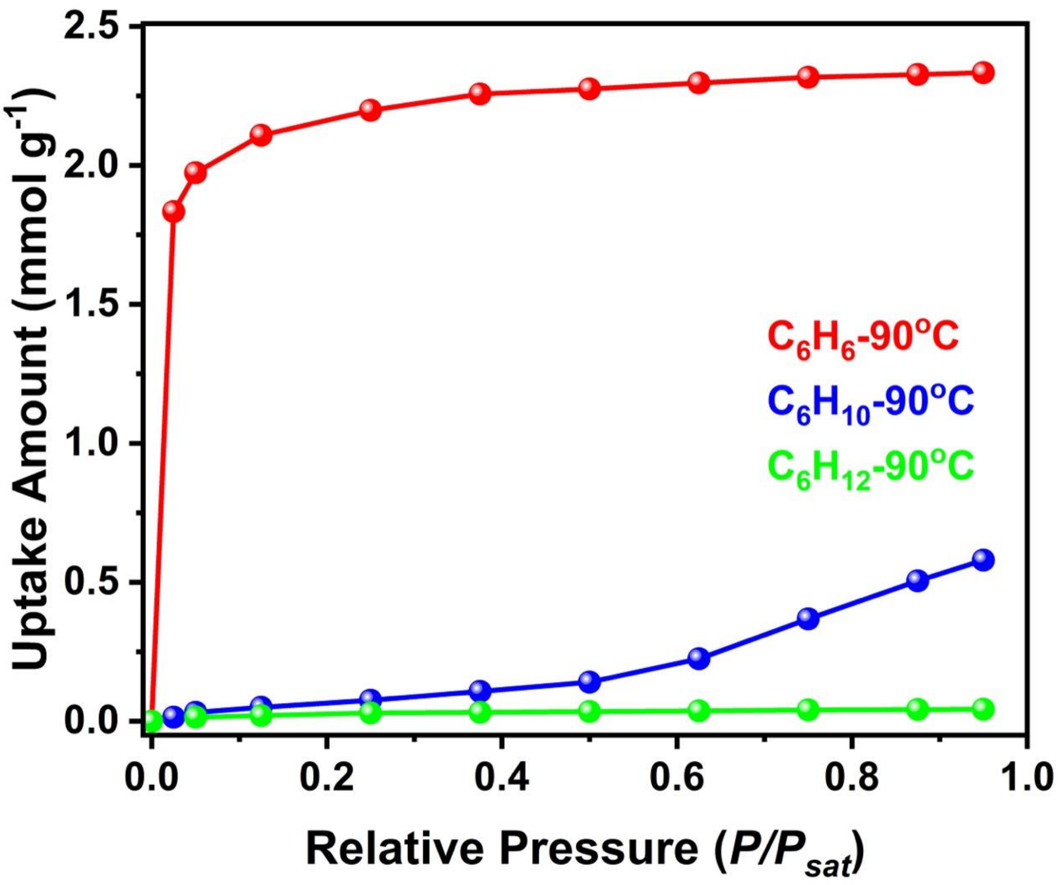

Supplementary Fig. 15. Dynamic adsorption isotherms of benzene (C_6H_6), cyclohexene (C_6H_{10}), and cyclohexane (C_6H_{12}) vapors on Mn-DHBQ sample at 90 °C.

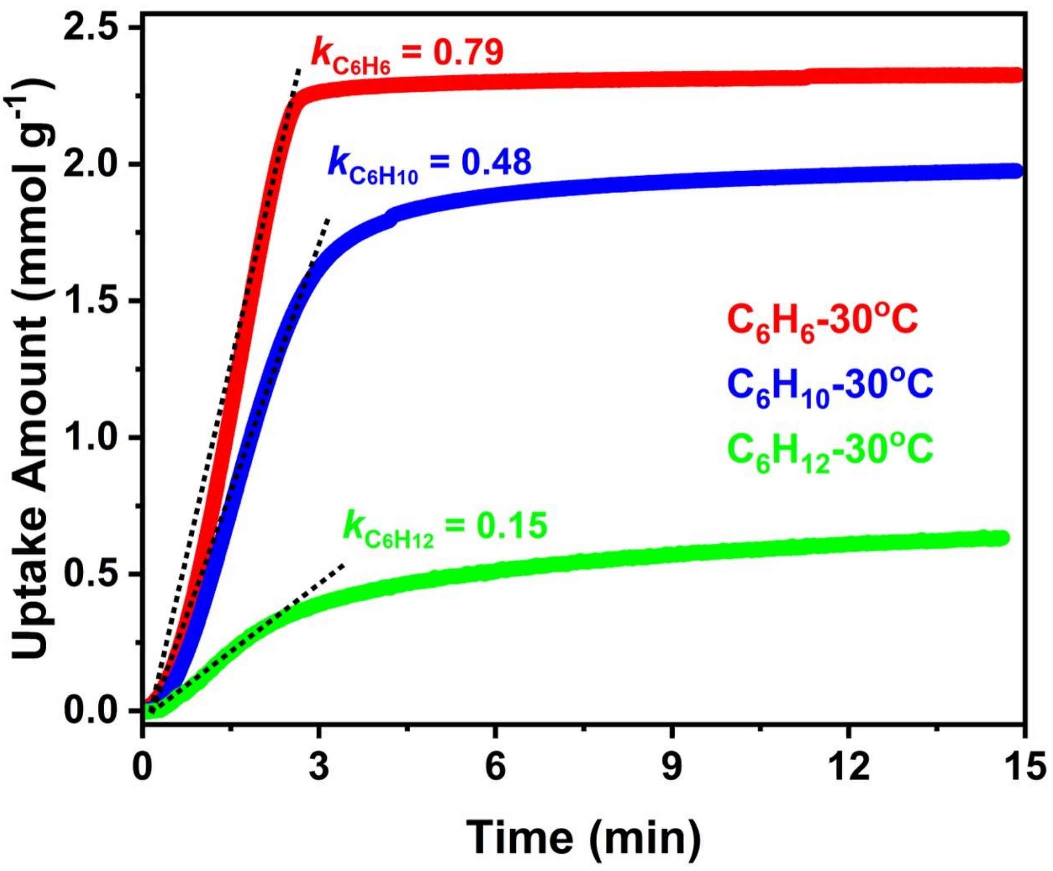

Supplementary Fig. 16. Adsorption kinetics of benzene (C_6H_6), cyclohexene (C_6H_{10}), and cyclohexane (C_6H_{12}) vapors on Mn-DHBQ sample at 30 °C and 0.3 relative pressure (P/P_{sat}). Slope k = uptake amount / time in the linear region.

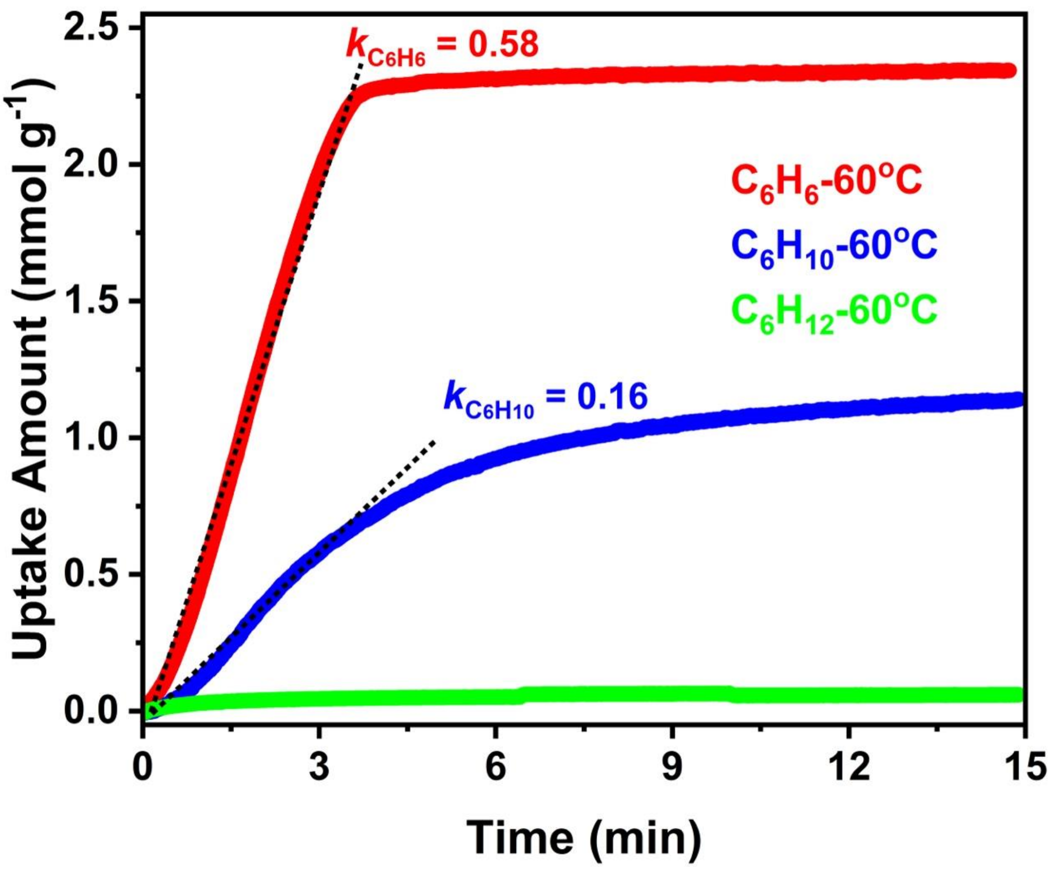

Supplementary Fig. 17. Adsorption kinetics of benzene (C_6H_6), cyclohexene (C_6H_{10}), and cyclohexane (C_6H_{12}) vapors on Mn-DHBQ sample at 60 °C and 0.3 relative pressure (P/P_{sat}). Slope k = uptake amount / time in the linear region.

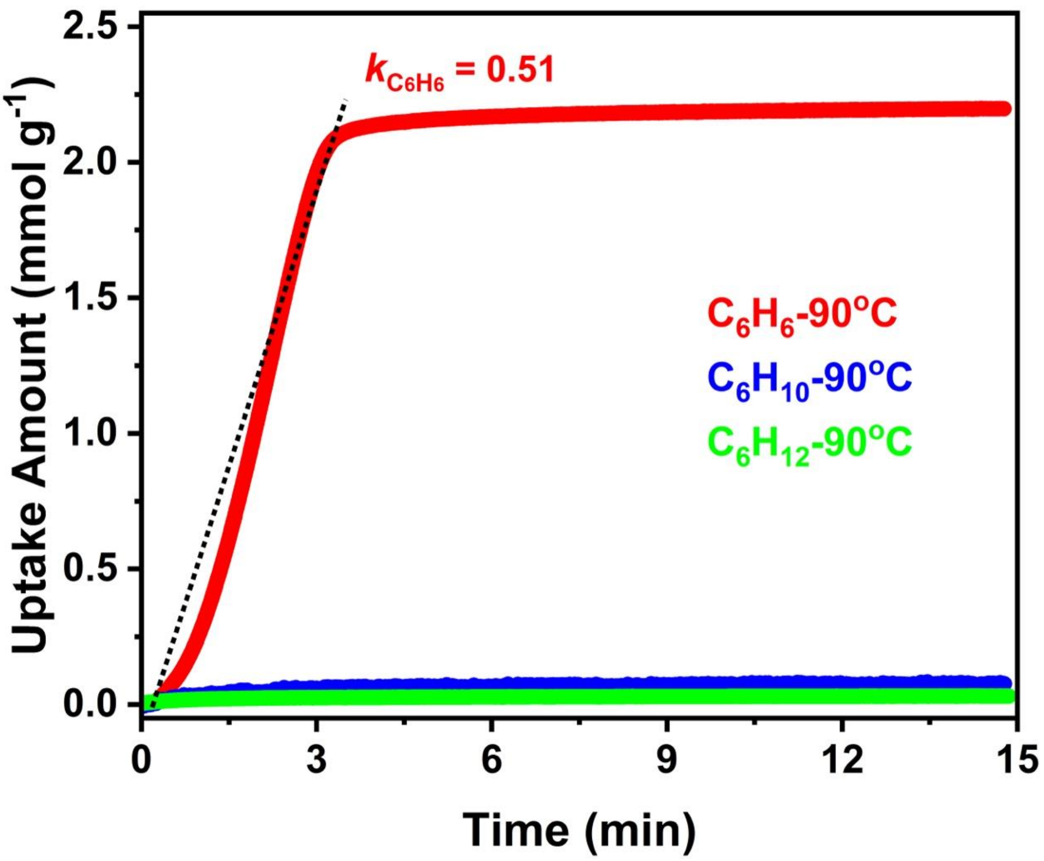

Supplementary Fig. 18. Adsorption kinetics of benzene (C_6H_6), cyclohexene (C_6H_{10}), and cyclohexane (C_6H_{12}) vapors on Mn-DHBQ sample at 90 °C and 0.3 relative pressure (P/P_{sat}). Slope k = uptake amount / time in the linear region.

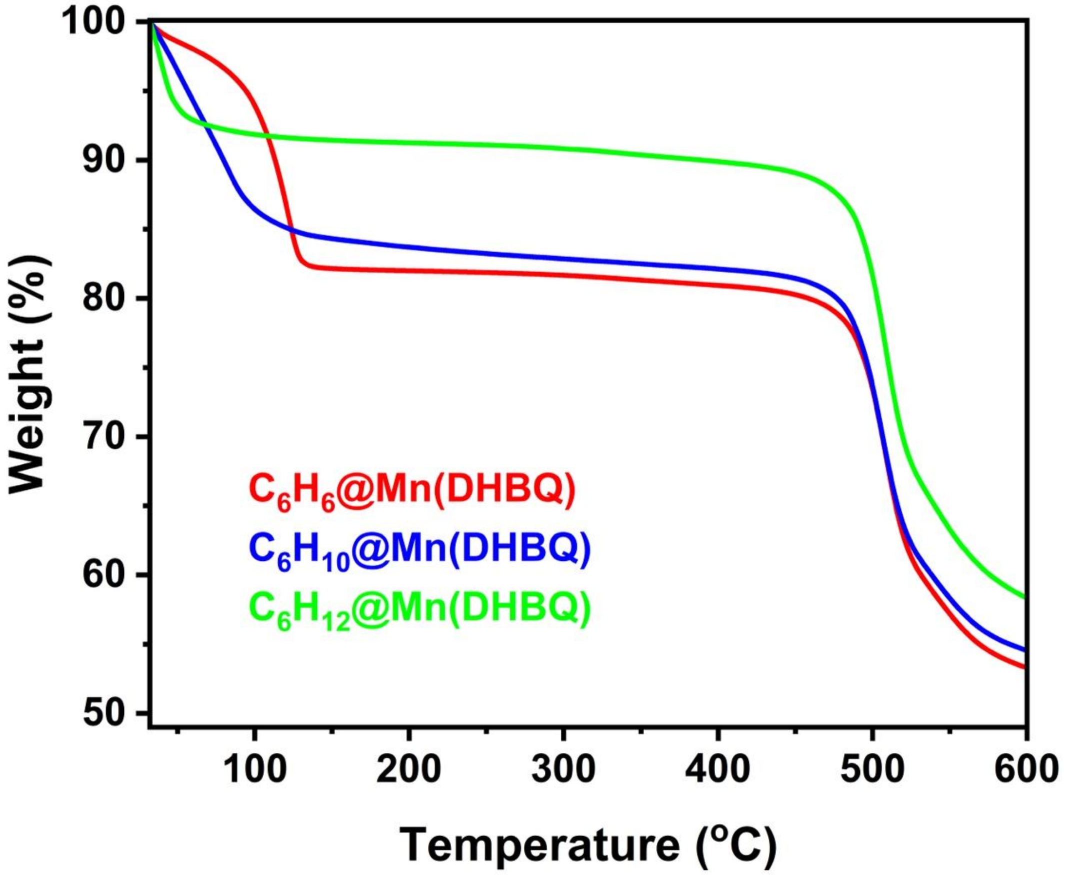

Supplementary Fig. 19. The thermogravimetric curves of fully hydrocarbon-loaded on activated samples for C_6H_6@Mn(DHBQ), C_6H_{10}@Mn(DHBQ), and C_6H_{12}@Mn(DHBQ) under nitrogen flowing environment with a heat rate of 5 °C min^{-1}.

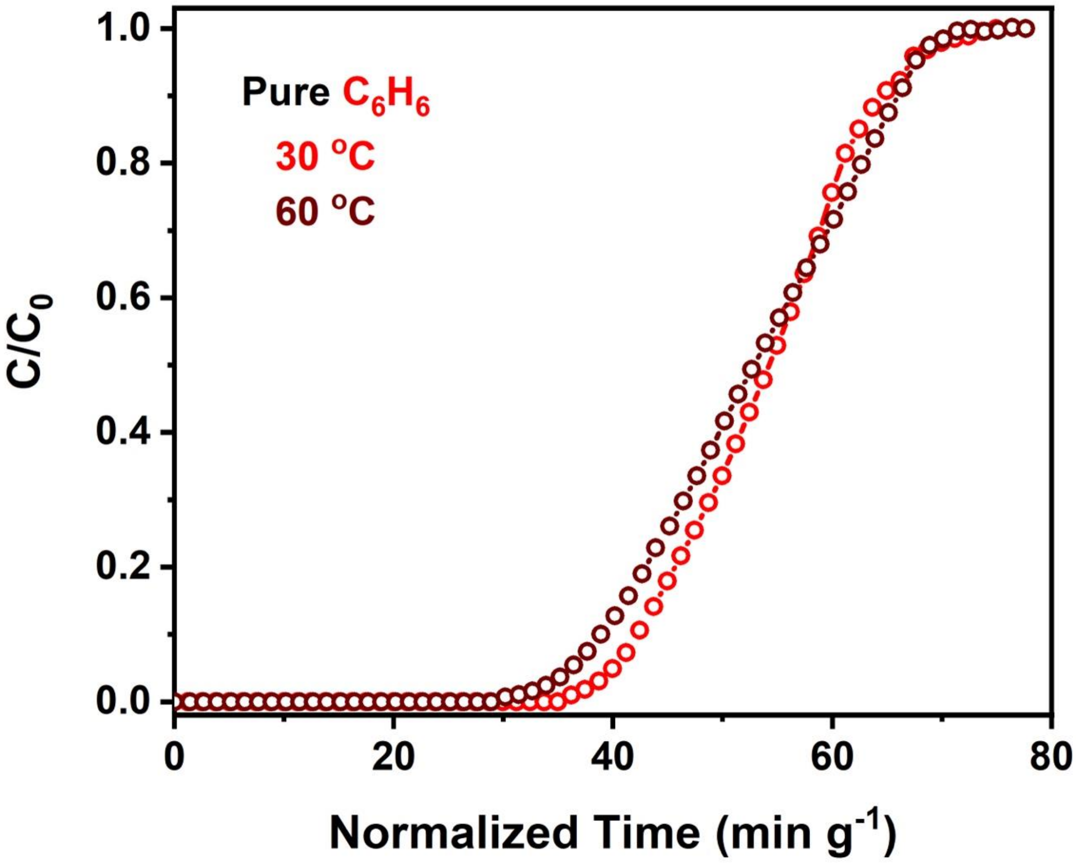

Supplementary Fig. 20. Vapor-phase breakthrough curves for pure benzene on Mn-DHBQ pellets packed in the single column at 30 and 60 °C. The benzene vapor phase was generated in the liquid hydrocarbon bubbler at 25 °C.

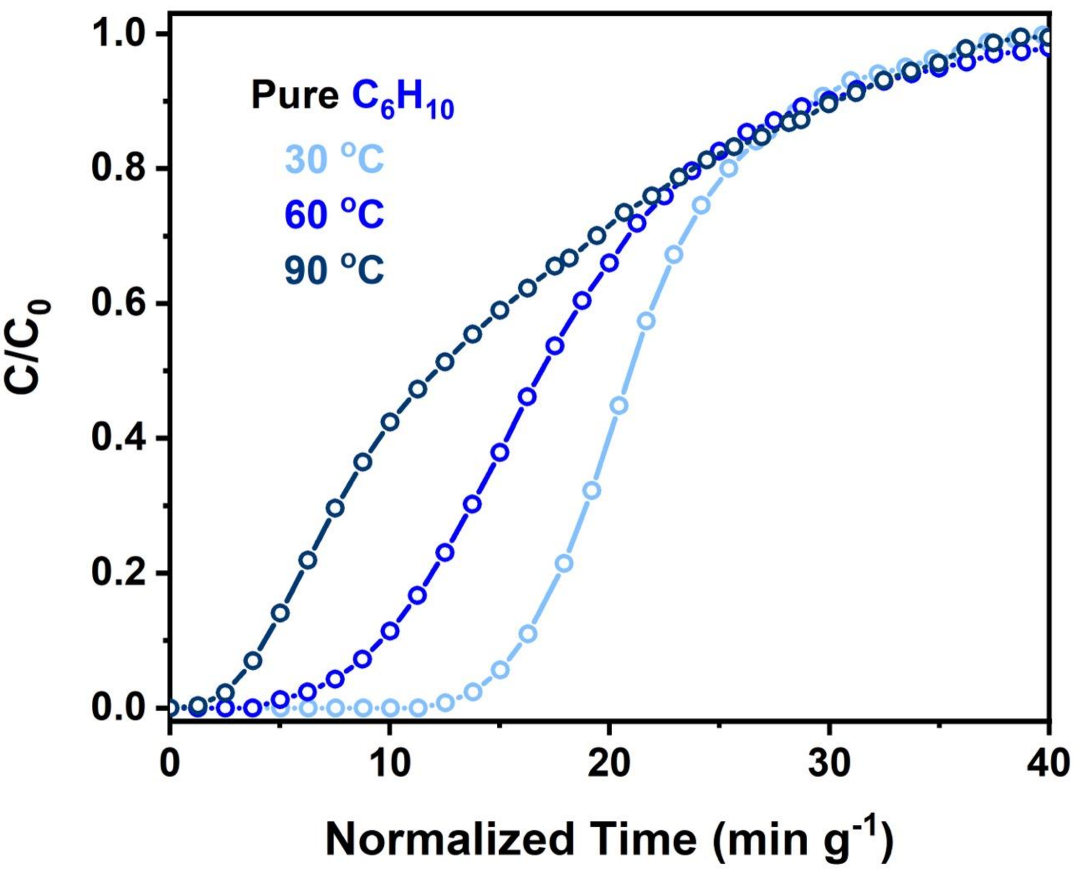

Supplementary Fig. 21. Vapor-phase breakthrough curves for pure cyclohexene on Mn-DHBQ pellets packed in the single column at 30, 60 and 90 °C. The cyclohexene vapor phase was generated in the liquid hydrocarbon bubbler at 25 °C.

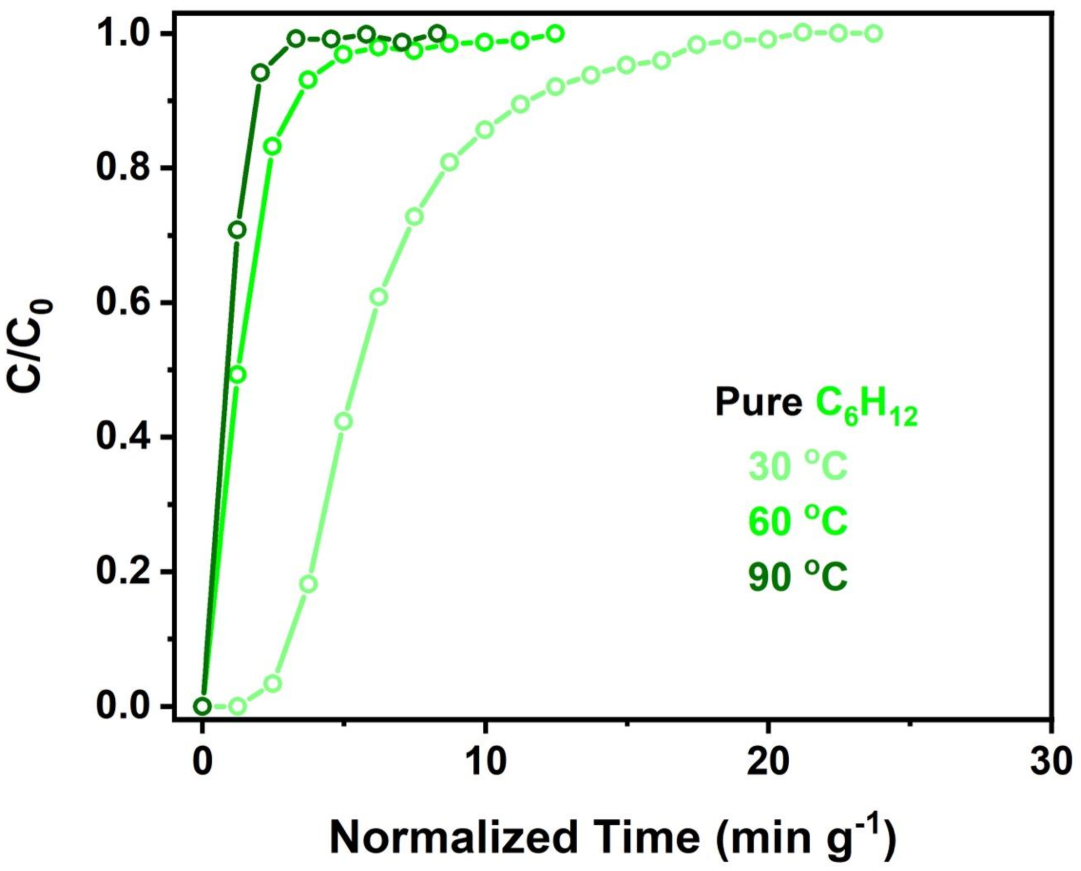

Supplementary Fig. 22. Vapor-phase breakthrough curves for pure cyclohexane on Mn-DHBQ pellets packed in the single column at 30, 60 and 90 °C. The cyclohexane vapor phase was generated in the liquid hydrocarbon bubbler at 25 °C.

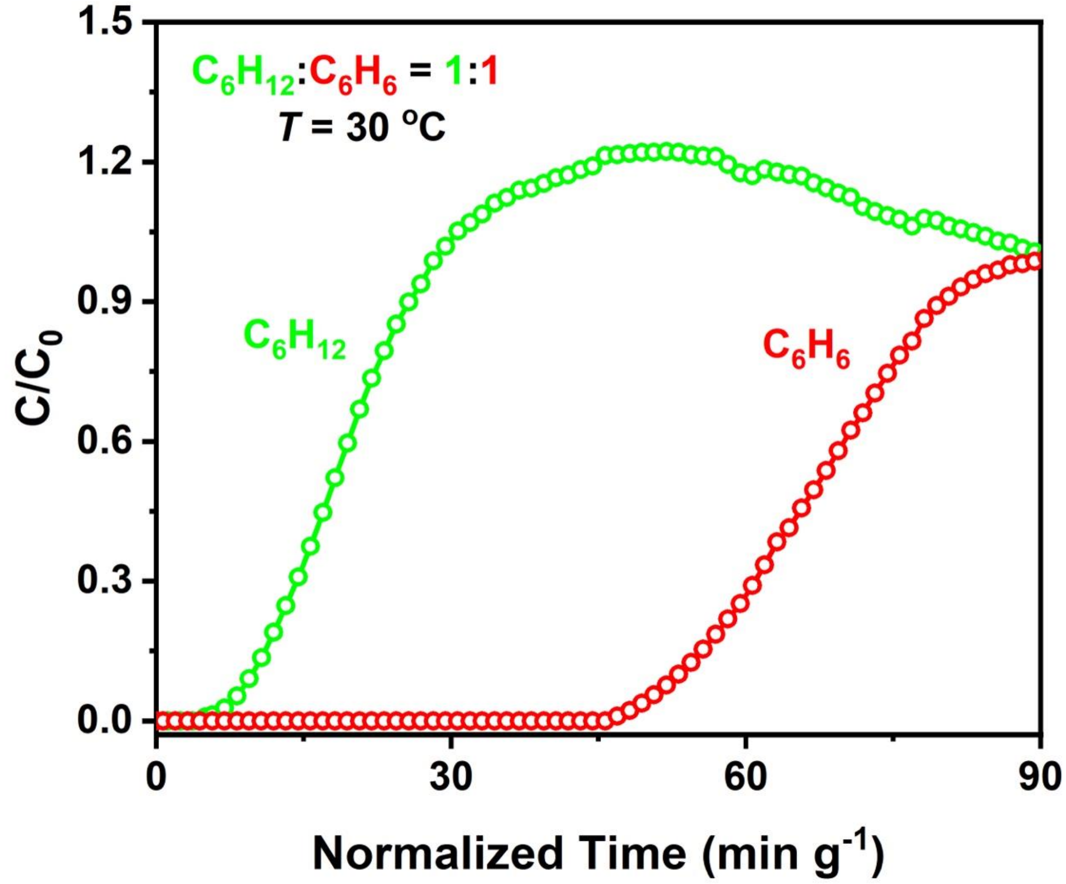

Supplementary Fig. 23. Two-component vapor-phase breakthrough curves for an equimolar binary mixture of benzene-cyclohexane on Mn-DHBQ pellets packed in the single column at 30 °C. The vapor phases were generated in the liquid hydrocarbon bubbler at 25 °C.

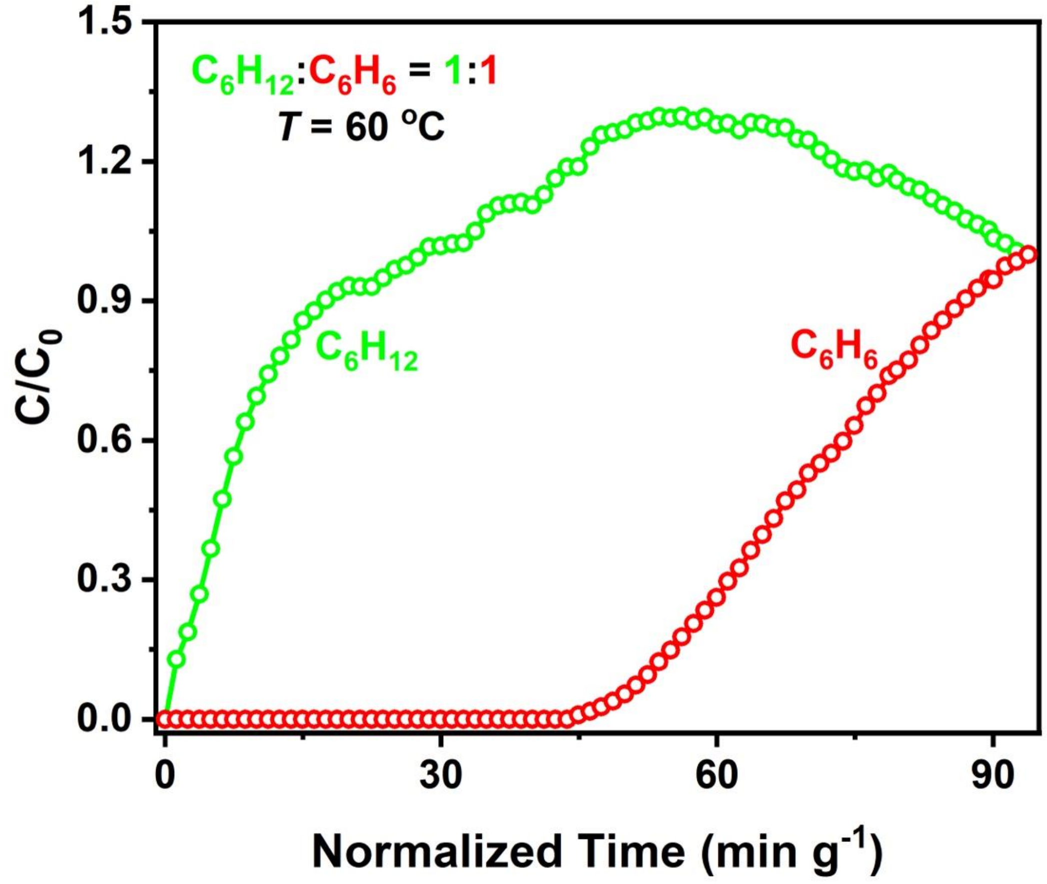

Supplementary Fig. 24. Two-component vapor-phase breakthrough curves for an equimolar binary mixture of benzene-cyclohexane on Mn-DHBQ pellets packed in the single column at 60 °C. The vapor phases were generated in the liquid hydrocarbon bubbler at 25 °C.

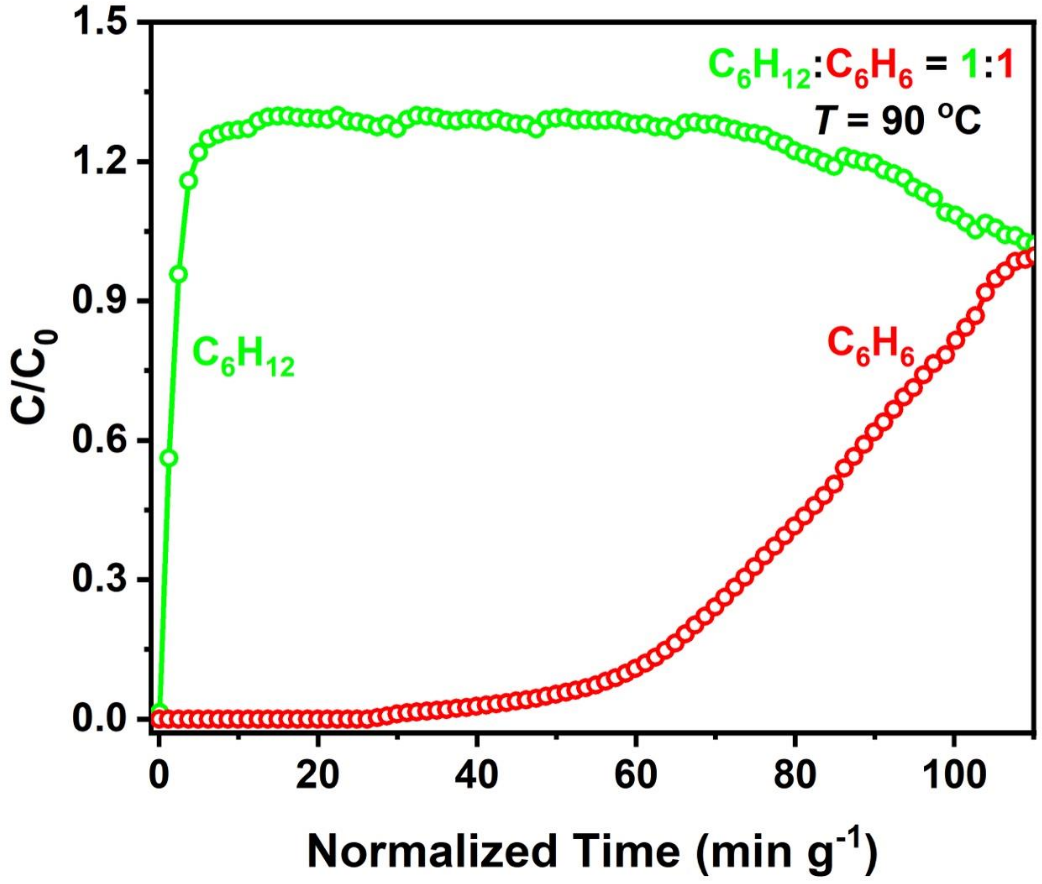

Supplementary Fig. 25. Two-component vapor-phase breakthrough curves for an equimolar binary mixture of benzene-cyclohexane on Mn-DHBQ pellets packed in the single column at 90 °C. The vapor phases were generated in the liquid hydrocarbon bubbler at 25 °C.

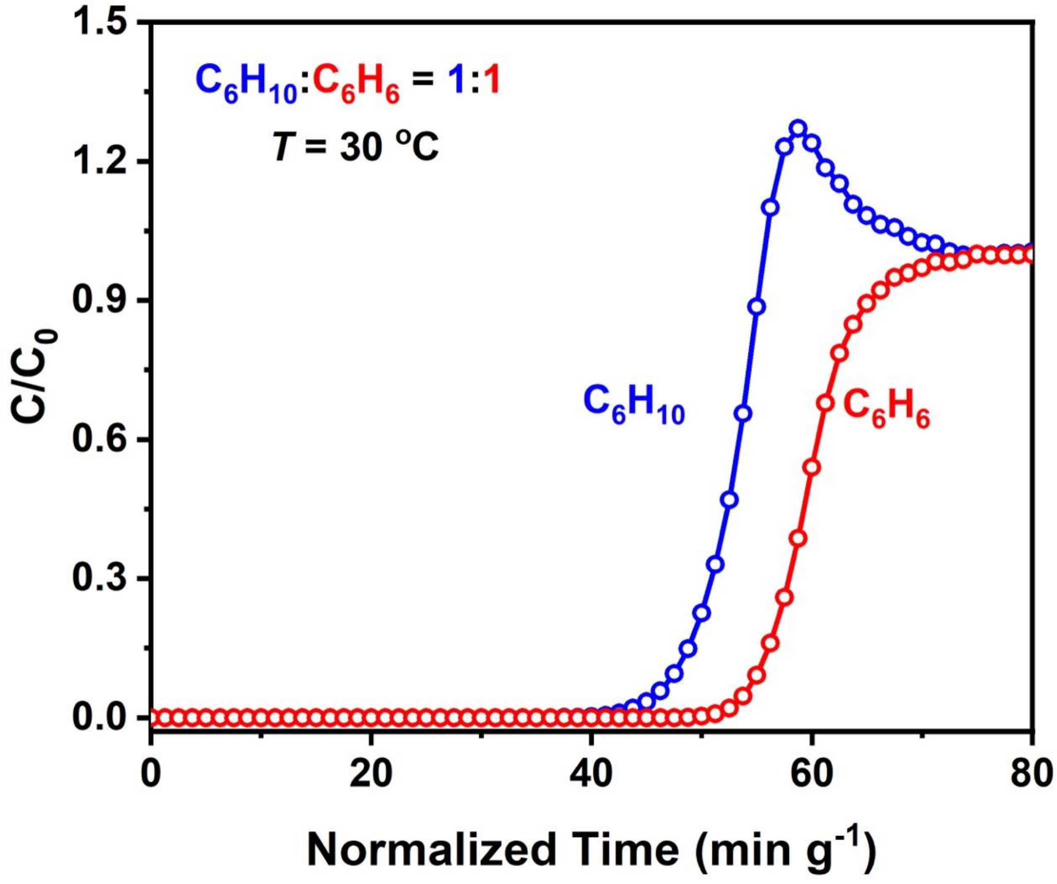

Supplementary Fig. 26. Two-component vapor-phase breakthrough curves for an equimolar binary mixture of benzene-cyclohexene on Mn-DHBQ pellets packed in the single column at 30 °C. The vapor phases were generated in the liquid hydrocarbon bubbler at 25 °C.

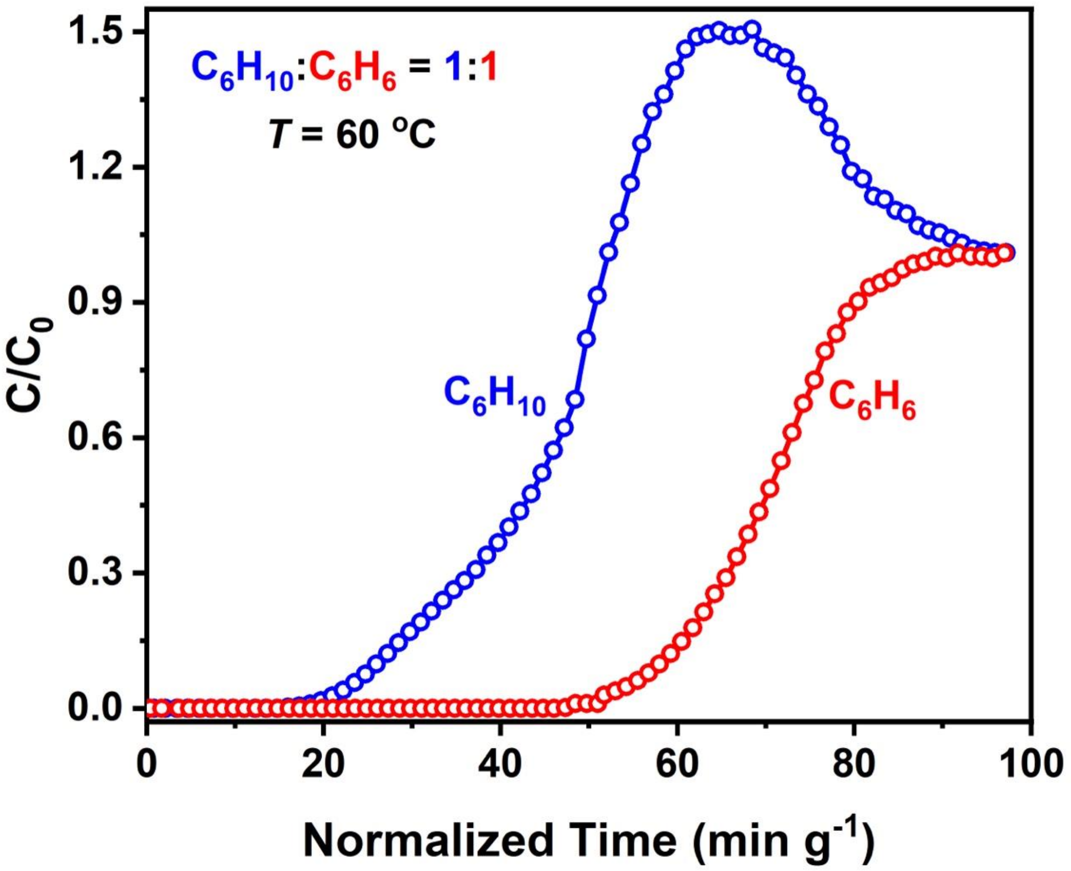

Supplementary Fig. 27. Two-component vapor-phase breakthrough curves for an equimolar binary mixture of benzene-cyclohexene on Mn-DHBQ pellets packed in the single column at 60 °C. The vapor phases were generated in the liquid hydrocarbon bubbler at 25 °C.

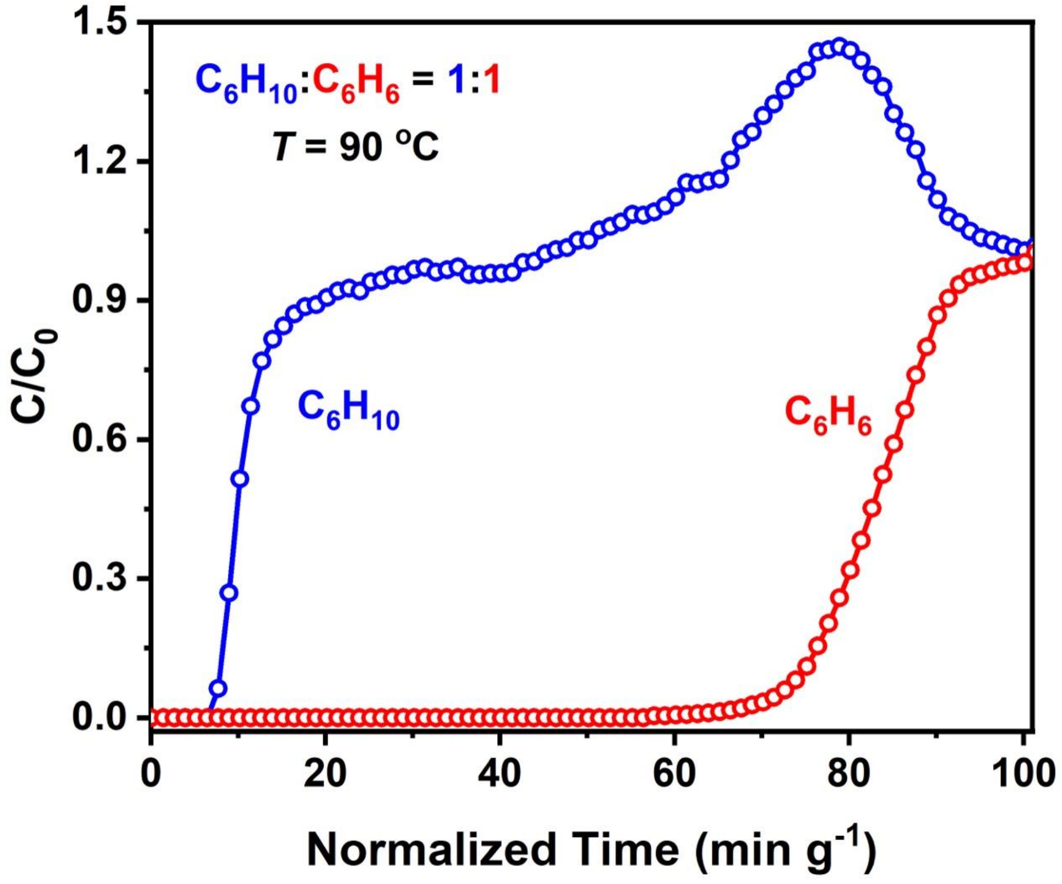

Supplementary Fig. 28. Two-component vapor-phase breakthrough curves for an equimolar binary mixture of benzene-cyclohexene on Mn-DHBQ pellets packed in the single column at 90 °C. The vapor phases were generated in the liquid hydrocarbon bubbler at 25 °C.

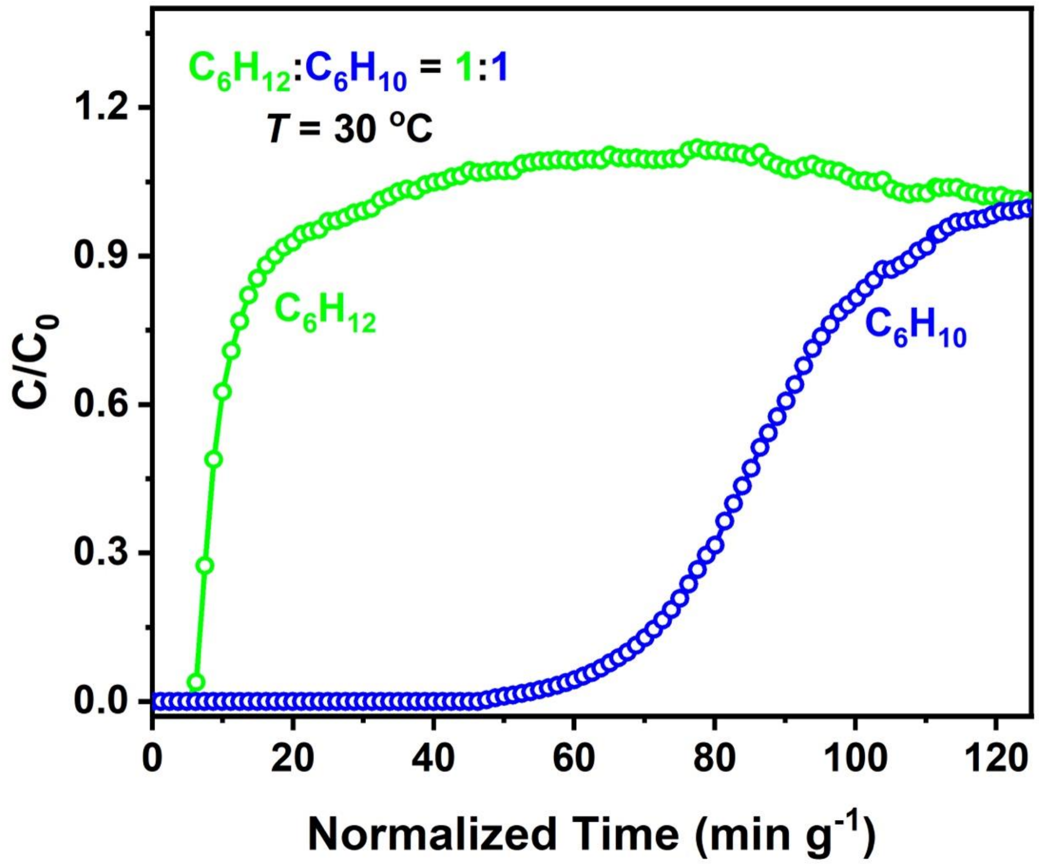

Supplementary Fig. 29. Two-component vapor-phase breakthrough curves for an equimolar binary mixture of cyclohexene-cyclohexane on Mn-DHBQ pellets packed in the single column at 30 °C. The vapor phases were generated in the liquid hydrocarbon bubbler at 25 °C.

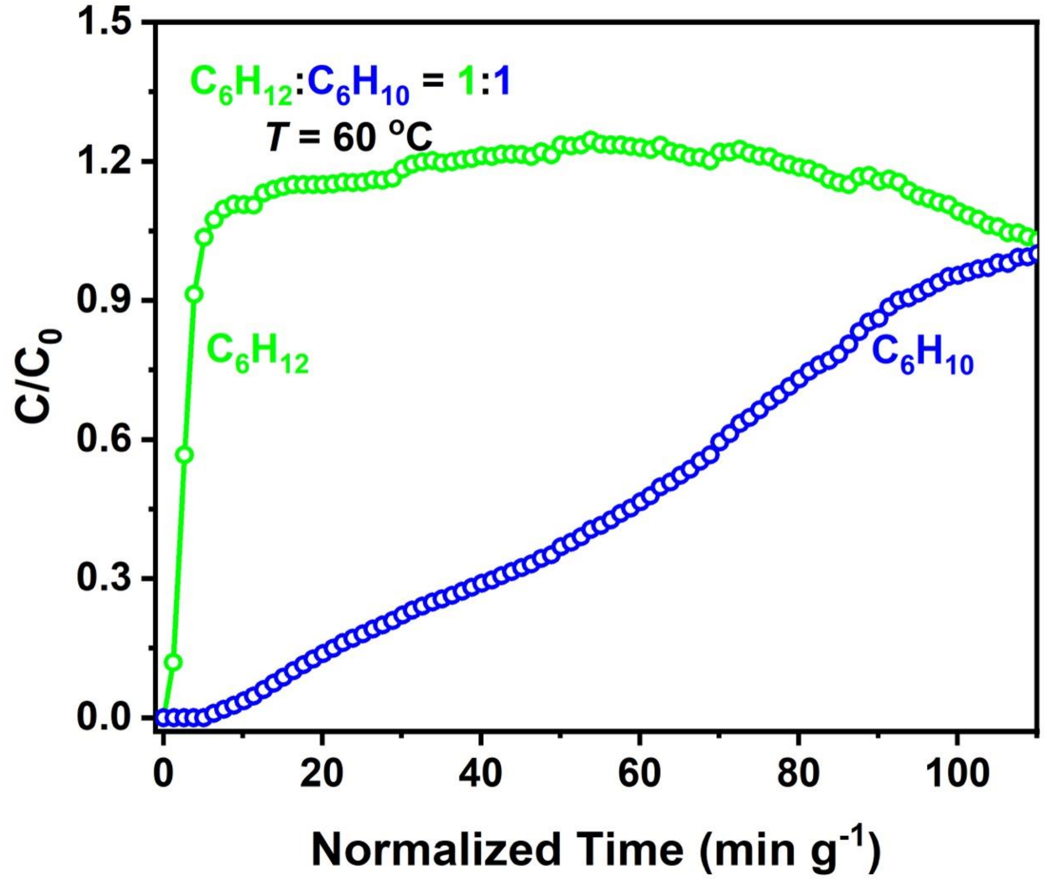

Supplementary Fig. 30. Two-component vapor-phase breakthrough curves for an equimolar binary mixture of cyclohexene-cyclohexane on Mn-DHBQ pellets packed in the single column at 60 °C. The vapor phases were generated in the liquid hydrocarbon bubbler at 25 °C.

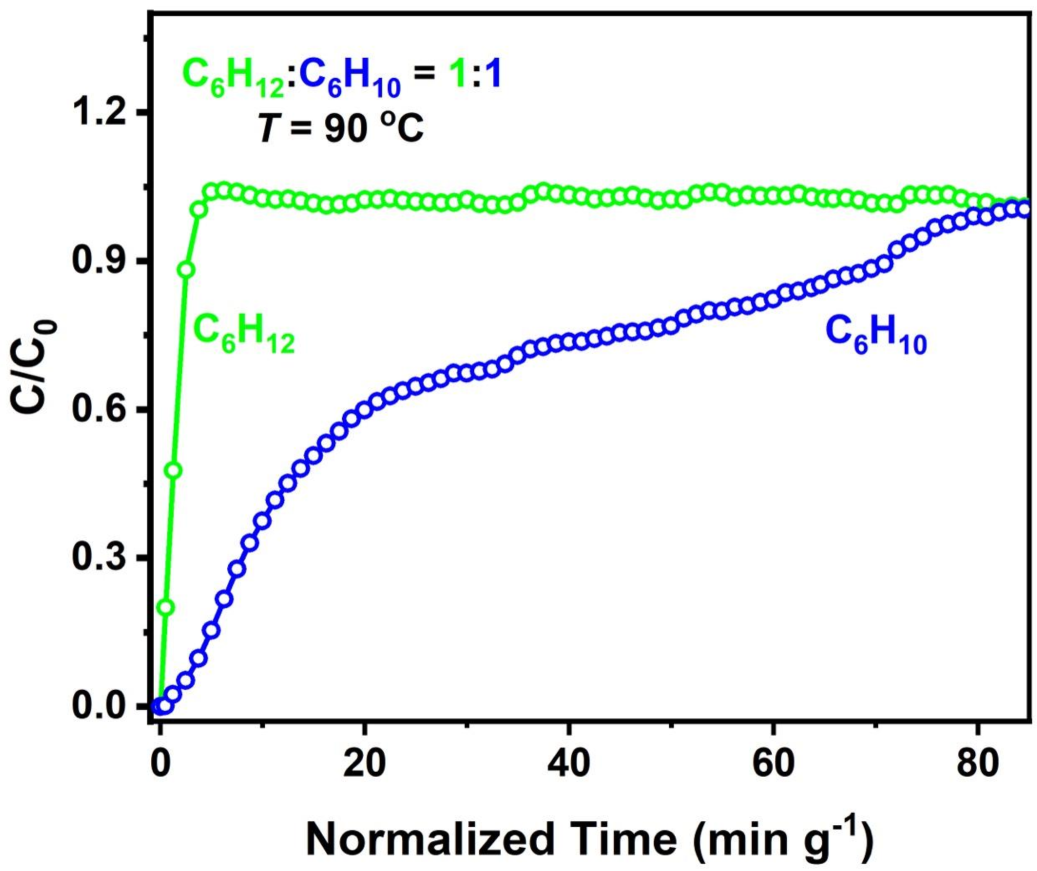

Supplementary Fig. 31. Two-component vapor-phase breakthrough curves for an equimolar binary mixture of cyclohexene-cyclohexane on Mn-DHBQ pellets packed in the single column at 90 °C. The vapor phases were generated in the liquid hydrocarbon bubbler at 25 °C.

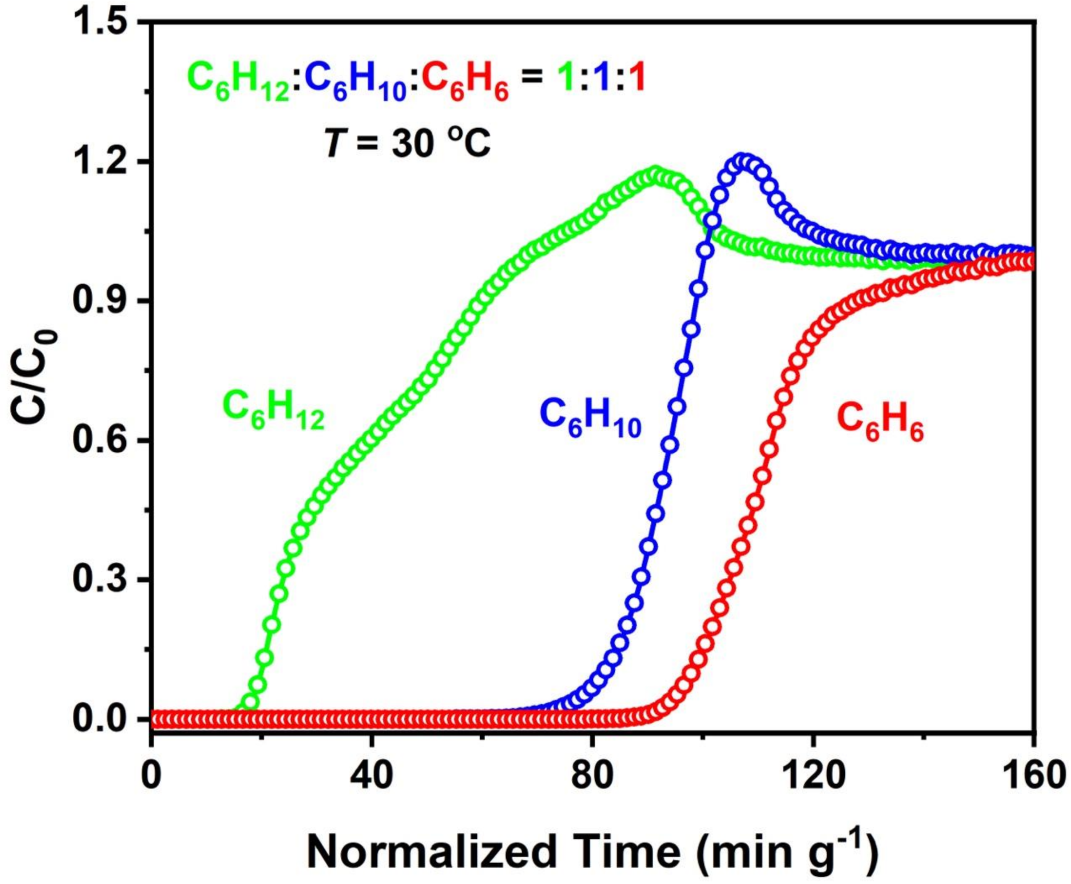

Supplementary Fig. 32. Three-component vapor-phase breakthrough curves for an equimolar ternary mixture of benzene-cyclohexene-cyclohexane on Mn-DHBQ pellets packed in the single column at 30 °C. The vapor phases were generated in the liquid hydrocarbon bubbler at 25 °C.

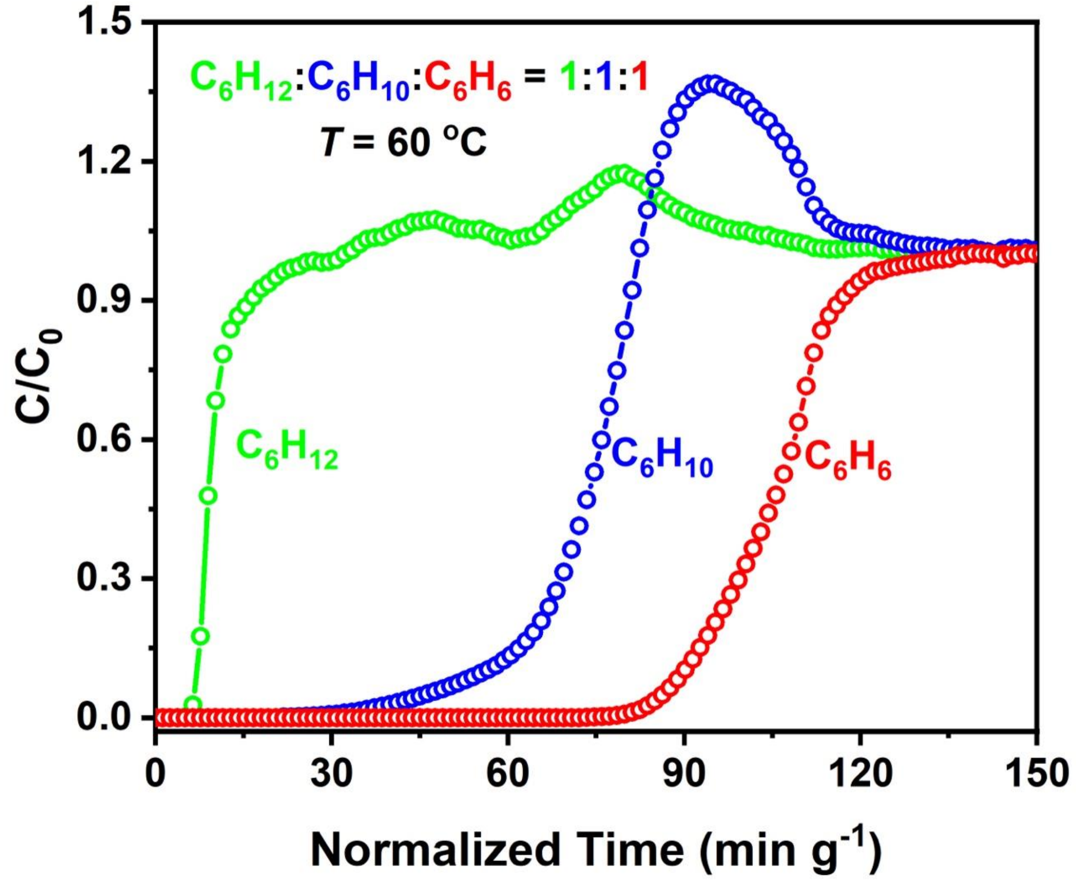

Supplementary Fig. 33. Three-component vapor-phase breakthrough curves for an equimolar ternary mixture of benzene-cyclohexene-cyclohexane on Mn-DHBQ pellets packed in the single column at 60 °C. The vapor phases were generated in the liquid hydrocarbon bubbler at 25 °C.

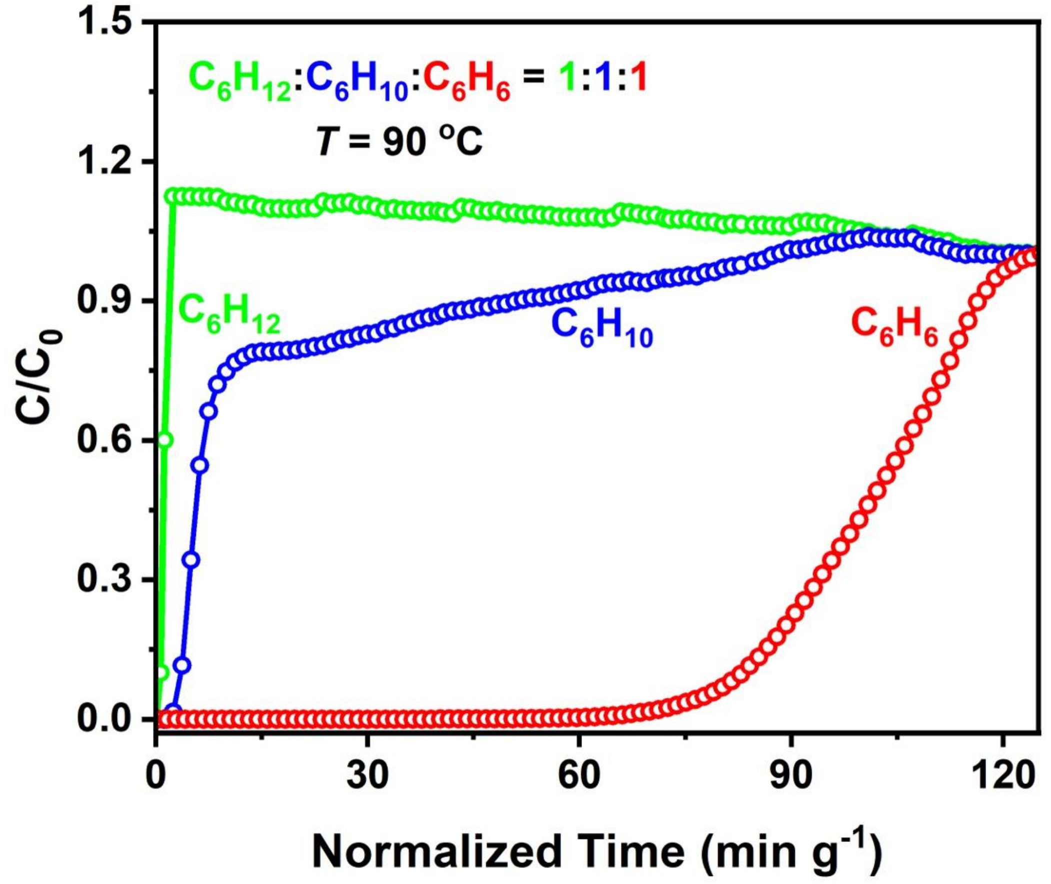

Supplementary Fig. 34. Three-component vapor-phase breakthrough curves for an equimolar ternary mixture of benzene-cyclohexene-cyclohexane on Mn-DHBQ pellets packed in the single column at 90 °C. The vapor phases were generated in the liquid hydrocarbon bubbler at 25 °C.

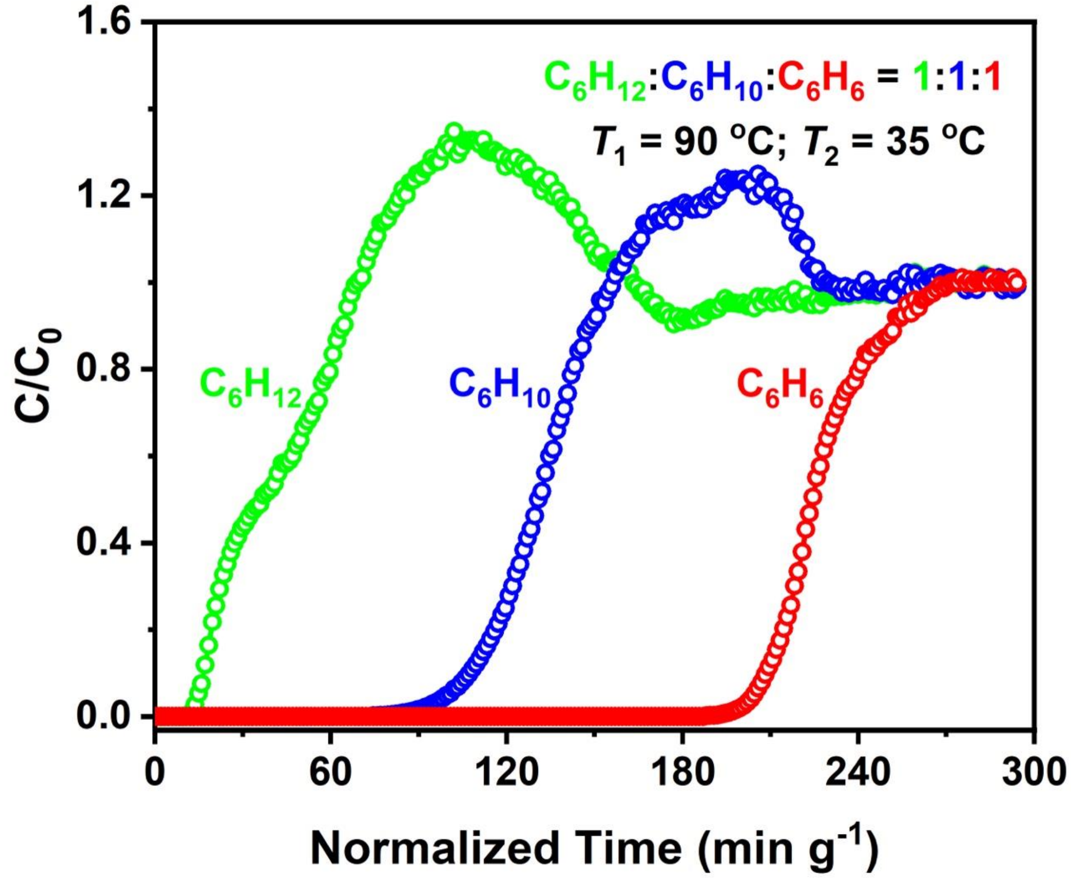

Supplementary Fig. 35. Three-component vapor-phase breakthrough curves for an equimolar ternary mixture of benzene-cyclohexene-cyclohexane on Mn-DHBQ pellets packed in two connected columns at 90 °C and 35 °C, respectively. The vapor phases were generated in the liquid hydrocarbon bubbler at 25 °C.

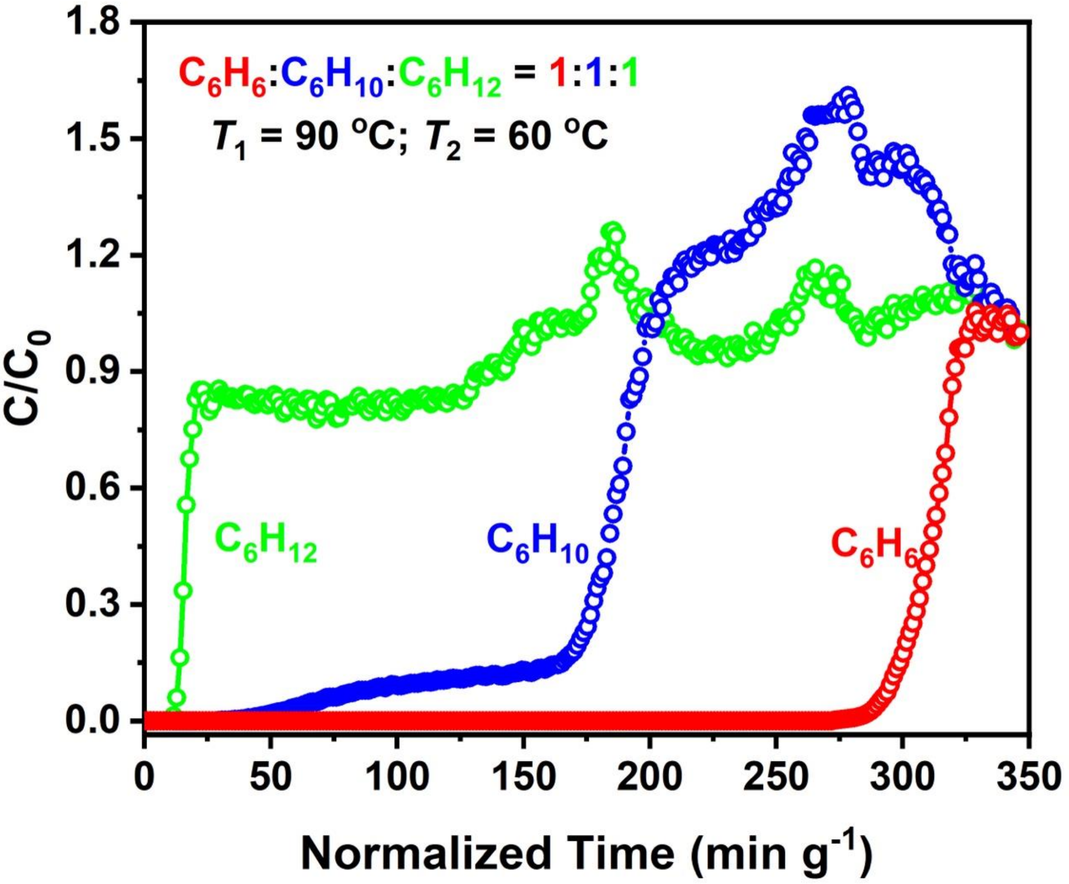

Supplementary Fig. 36. Three-component vapor-phase breakthrough curves for an equimolar ternary mixture of benzene-cyclohexene-cyclohexane on Mn-DHBQ pellets packed in two connected columns at 90 °C and 60 °C, respectively. The vapor phases were generated in the liquid hydrocarbon bubbler at 25 °C.

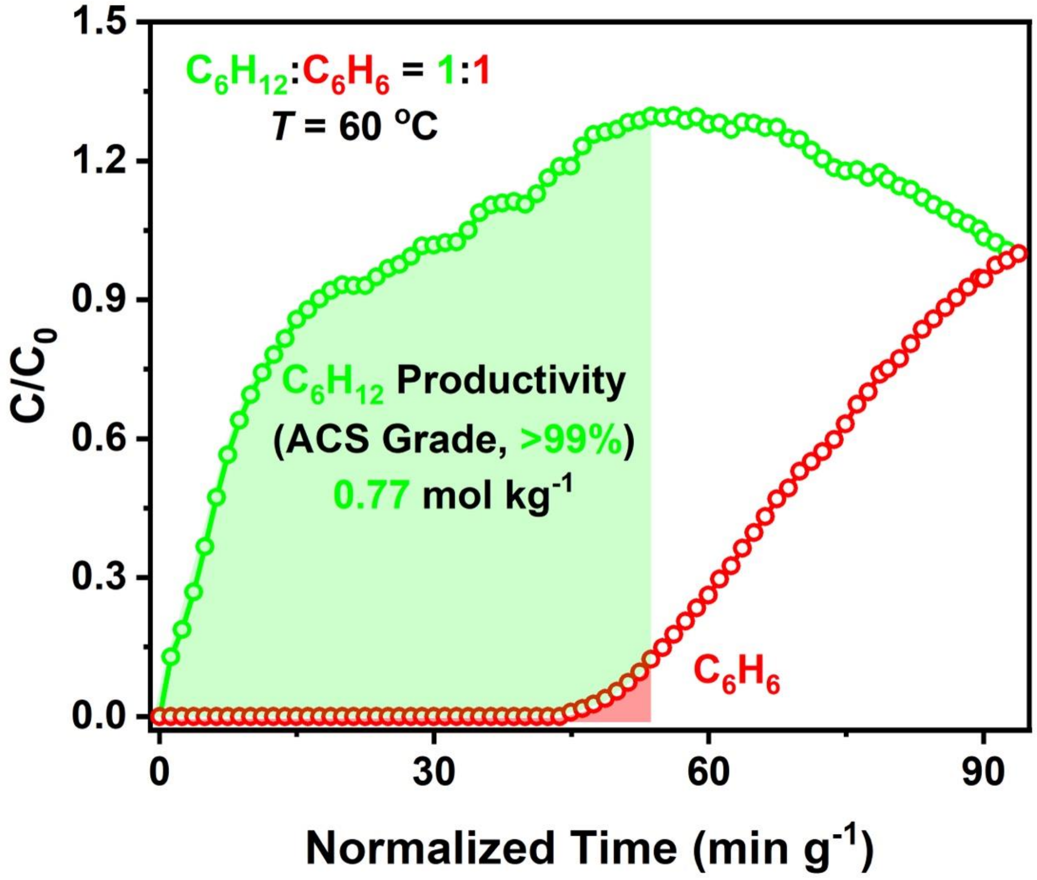

Supplementary Fig. 37. Production of ACS grade C_6H_{12} from an equimolar binary mixture of benzene-cyclohexane on Mn-DHBQ pellets packed in the single column at 60 °C. The productivity was calculated by integration of the area.

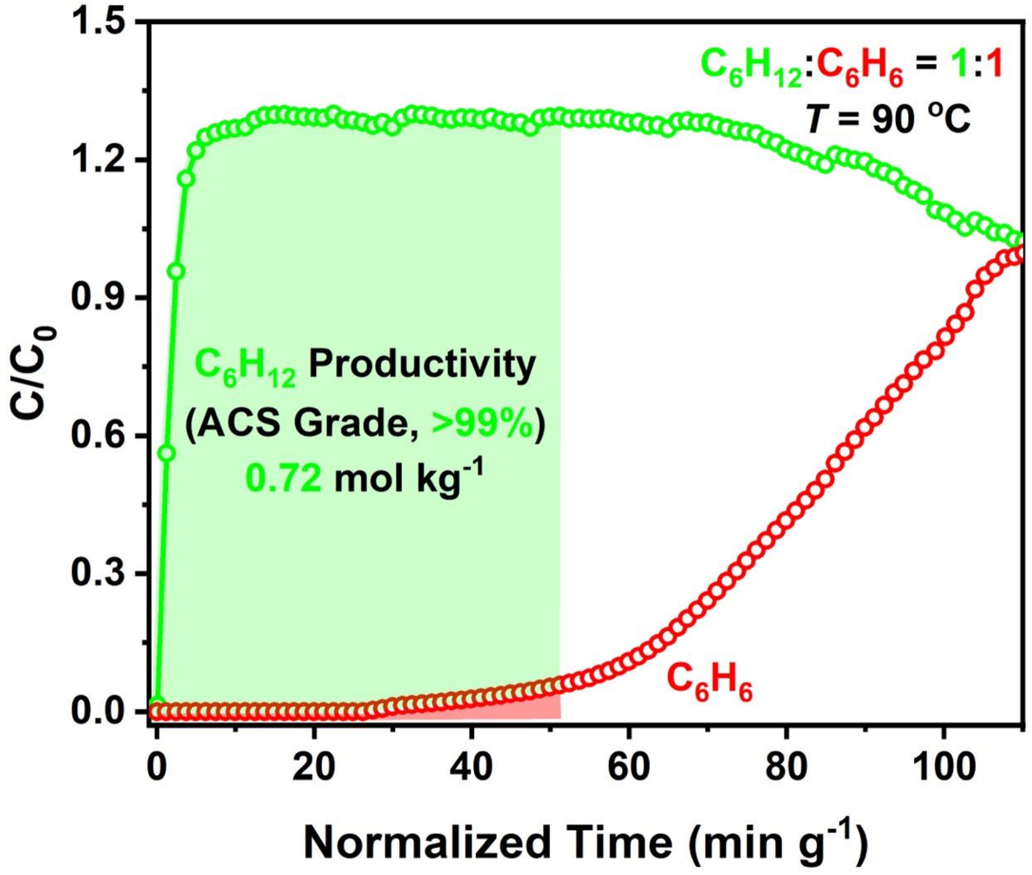

Supplementary Fig. 38. Production of ACS grade C_6H_{12} from an equimolar binary mixture of benzene-cyclohexane on Mn-DHBQ pellets packed in the single column at 90 °C. The productivity was calculated by integration of the area.

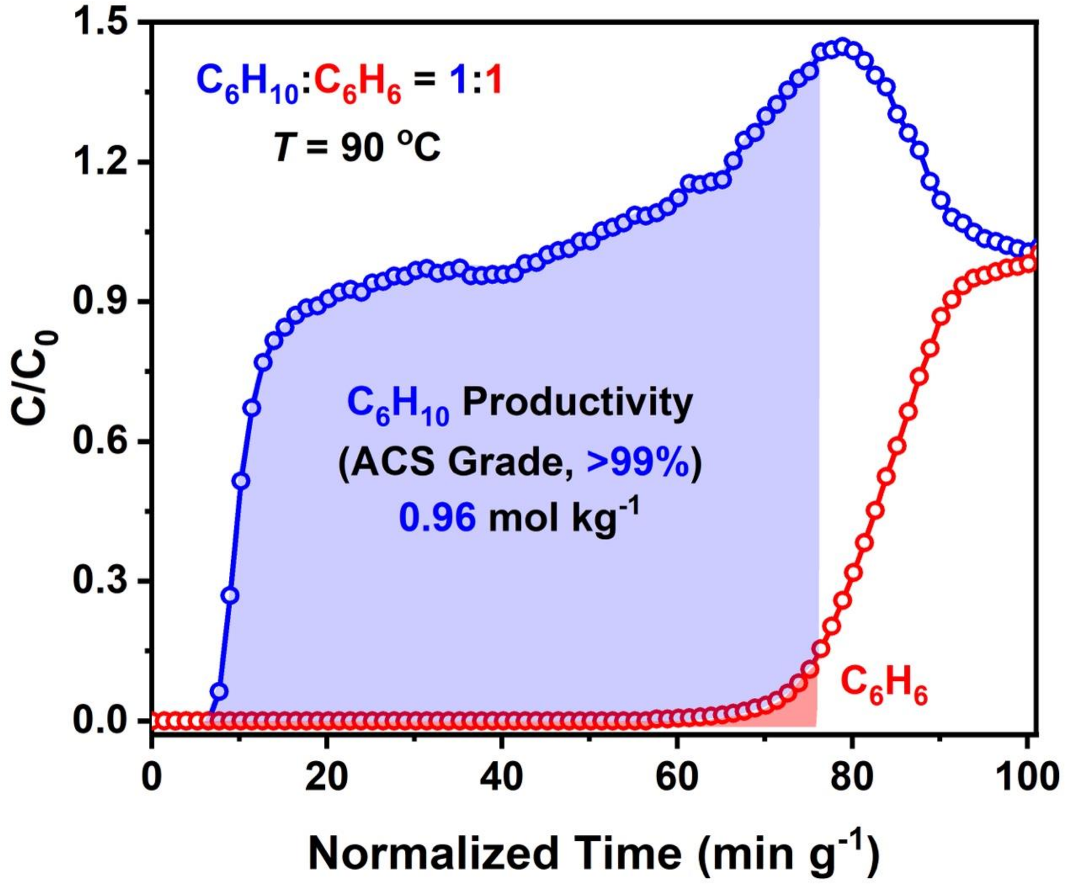

Supplementary Fig. 39. Production of ACS grade C_6H_{10} from an equimolar binary mixture of benzene-cyclohexene on Mn-DHBQ pellets packed in the single column at 90 °C. The productivity was calculated by integration of the area.

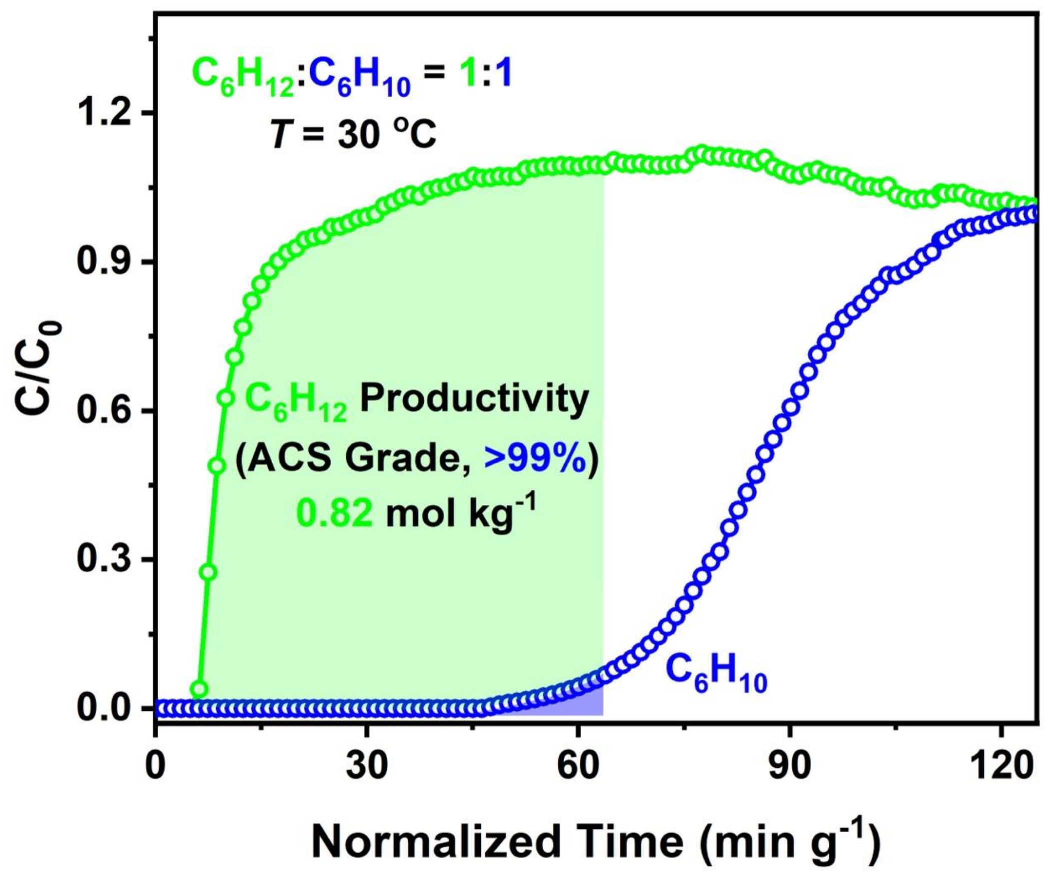

Supplementary Fig. 40. Production of ACS grade C_6H_{12} from an equimolar binary mixture of cyclohexene-cyclohexane on Mn-DHBQ pellets packed in the single column at 30 °C. The productivity was calculated by integration of the area.

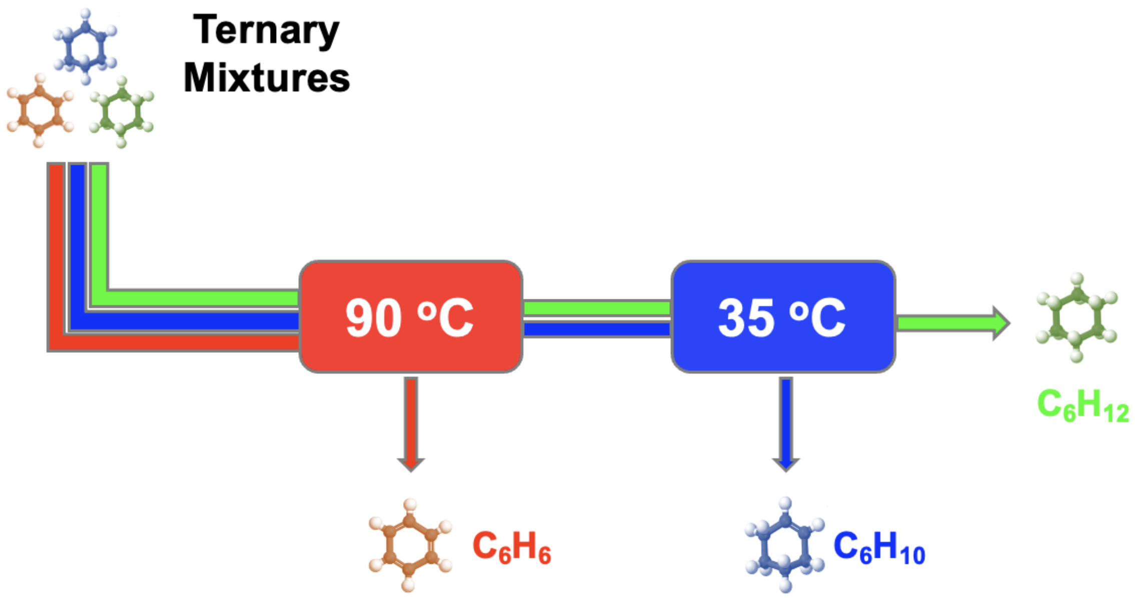

Supplementary Fig. 41. Schematic representation of the separation of benzene-cyclohexene-cyclohexane ternary mixture based on molecular sieving mechanism using two columns set at different temperatures.

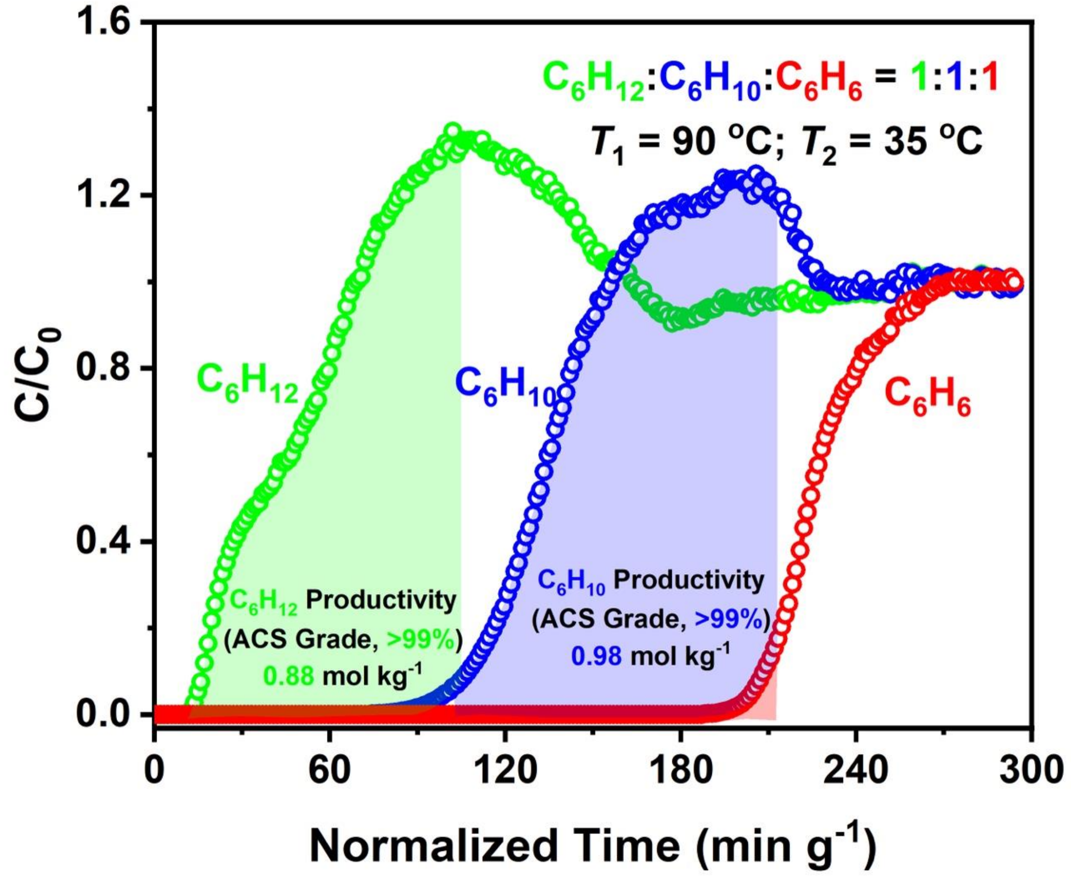

Supplementary Fig. 42. Production of ACS grade C_6H_{12} and C_6H_{10} for an equimolar ternary mixture of benzene-cyclohexene-cyclohexane on Mn-DHBQ pellets packed in two connected columns at 90 °C and 35 °C, respectively. The productivity was calculated by integration of the area.

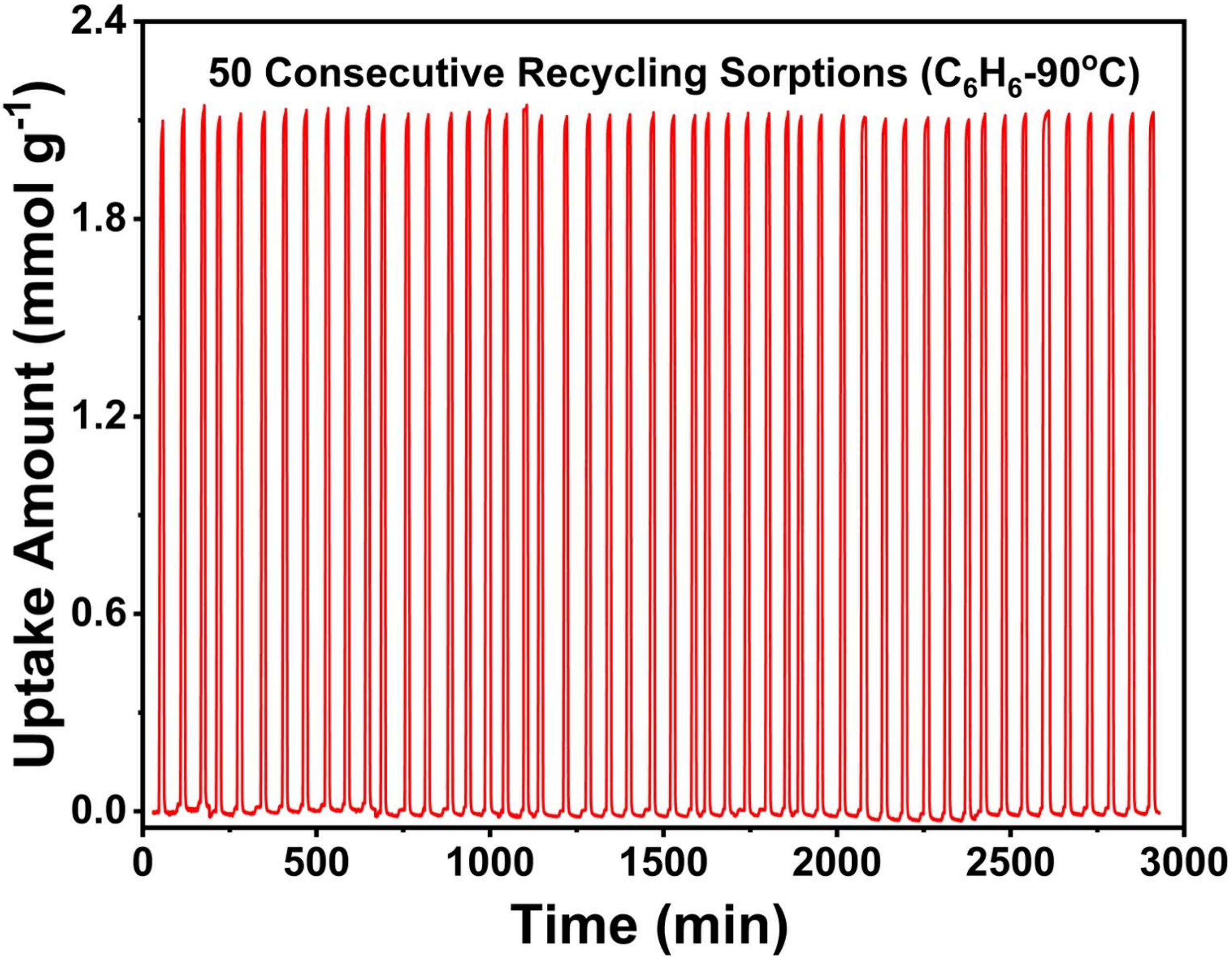

Supplementary Fig. 43. Adsorption-desorption recyclability test results for benzene on Mn-DHBQ for 50 consecutive sorption cycles at 90 °C.

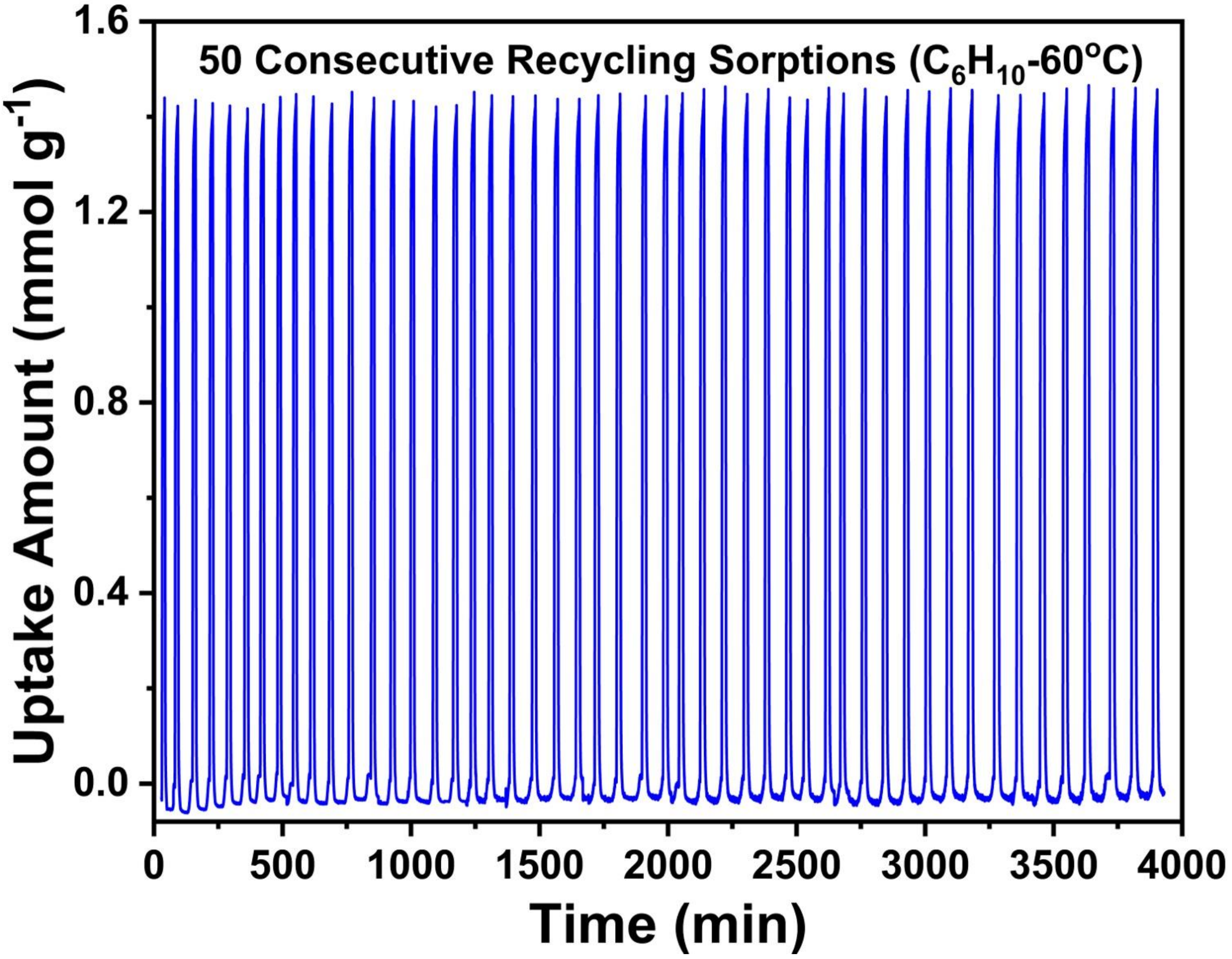

Supplementary Fig. 44. Adsorption-desorption recyclability test results for cyclohexene on Mn-DHBQ for 50 consecutive sorption cycles at 60 °C.

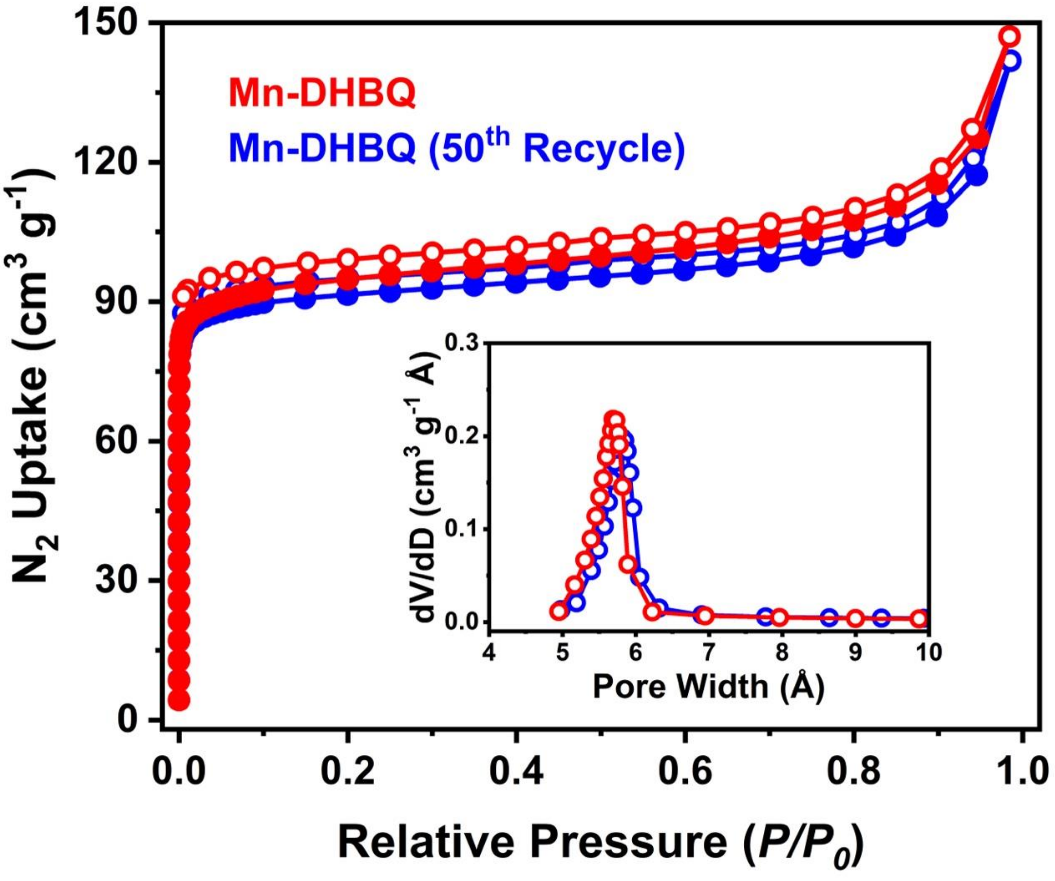

Supplementary Fig. 45. The N_2 sorption isotherms at 77 K and related pore size distribution of Mn-DHBQ samples after 50 consecutive sorption cycles.

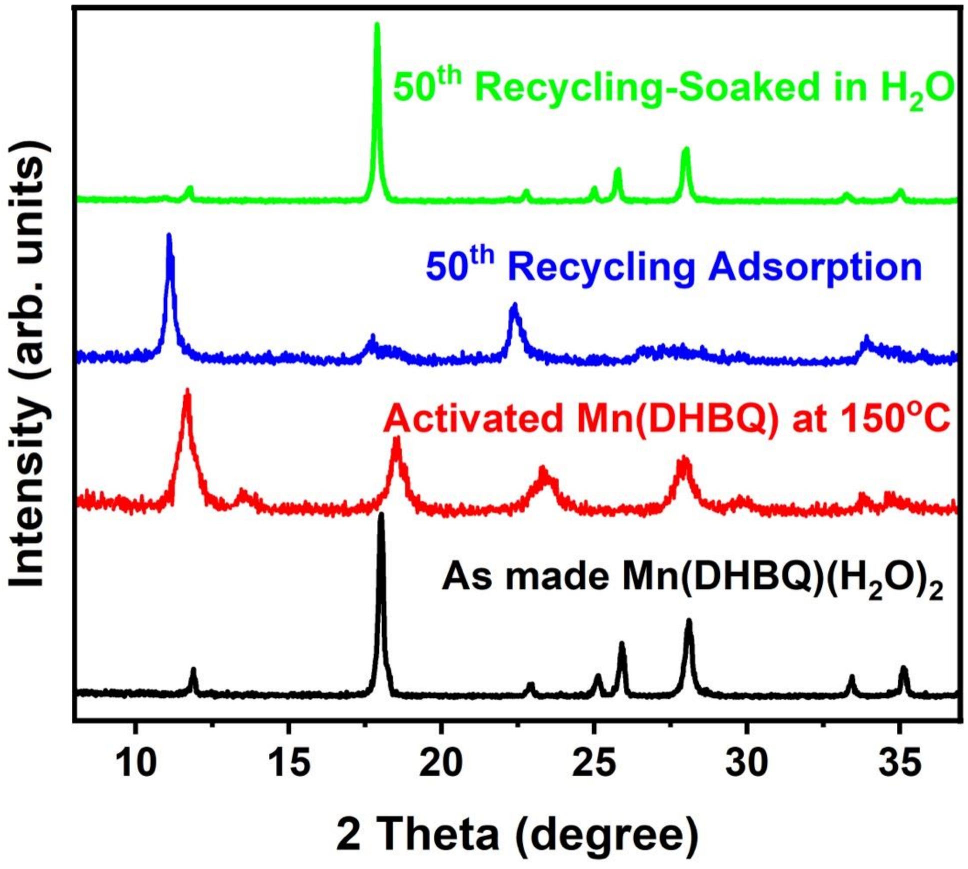

Supplementary Fig. 46. The PXRD patterns of the activated Mn-DHBQ samples before and after 50 consecutive C_6H_6 sorption cycles along with those of as-made and recovered samples.

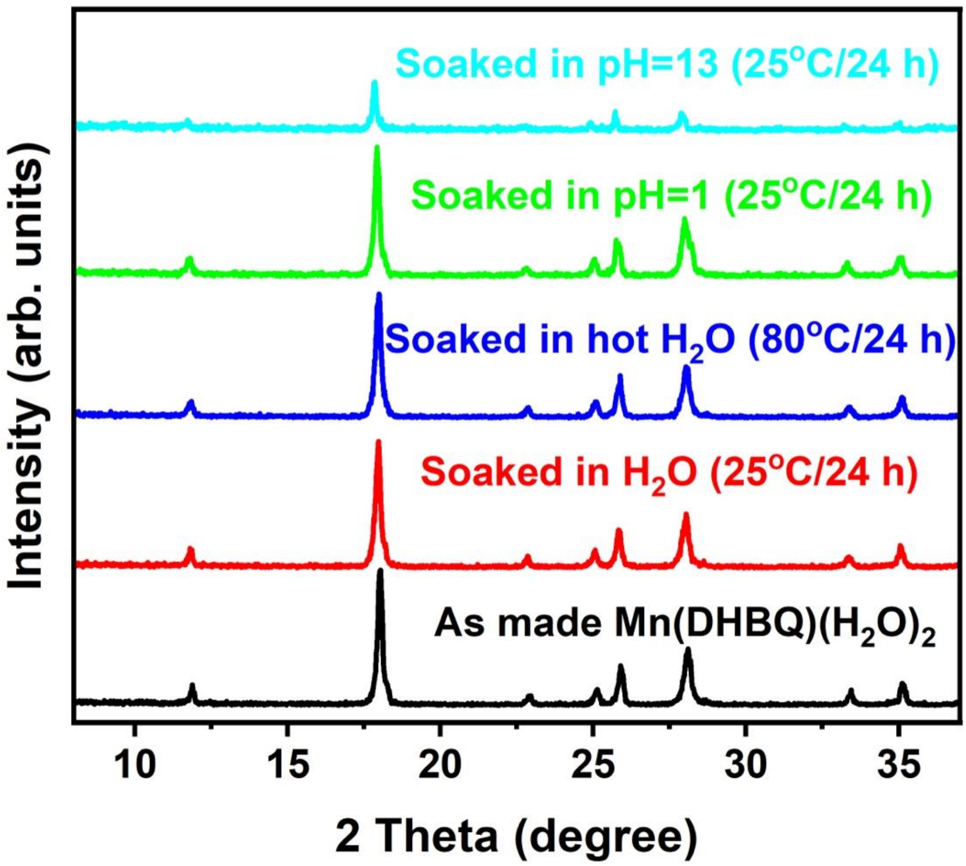

Supplementary Fig. 47. The PXRD patterns of Mn-DHBQ samples after various treatments.

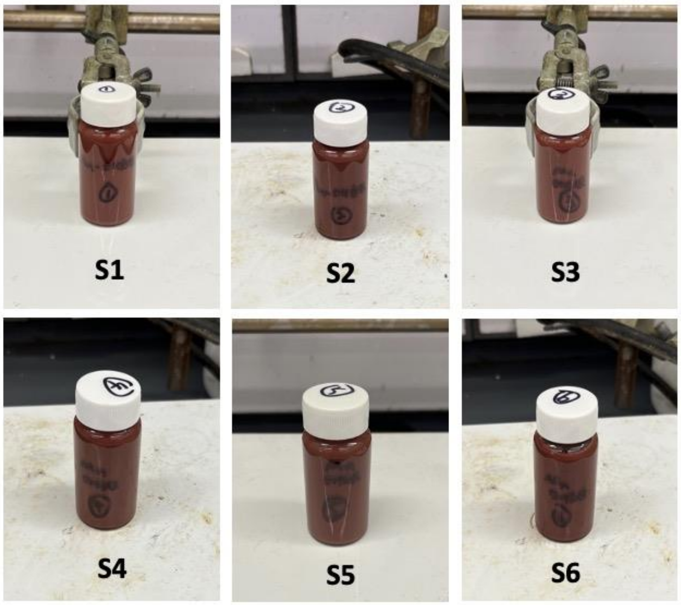

Sample	Product (mg)	Yield (%)
S1	222.4	97.1
S2	219.1	95.7
S3	224.1	97.9
S4	223.7	97.7
S5	222.3	97.1
S6	221.1	96.6

Reaction: 1 mmol $Mn(OAc)_2$ was mixed with 1 mmol H_2DHBQ in a 20 mL aqueous solution, and the mixture was stirred at room temperature overnight. The resulting products were collected via centrifugation and subsequently dried under ambient conditions.

Supplementary Fig. 48. Reproducible preparation (six batches) of Mn-DHBQ samples and product yields.

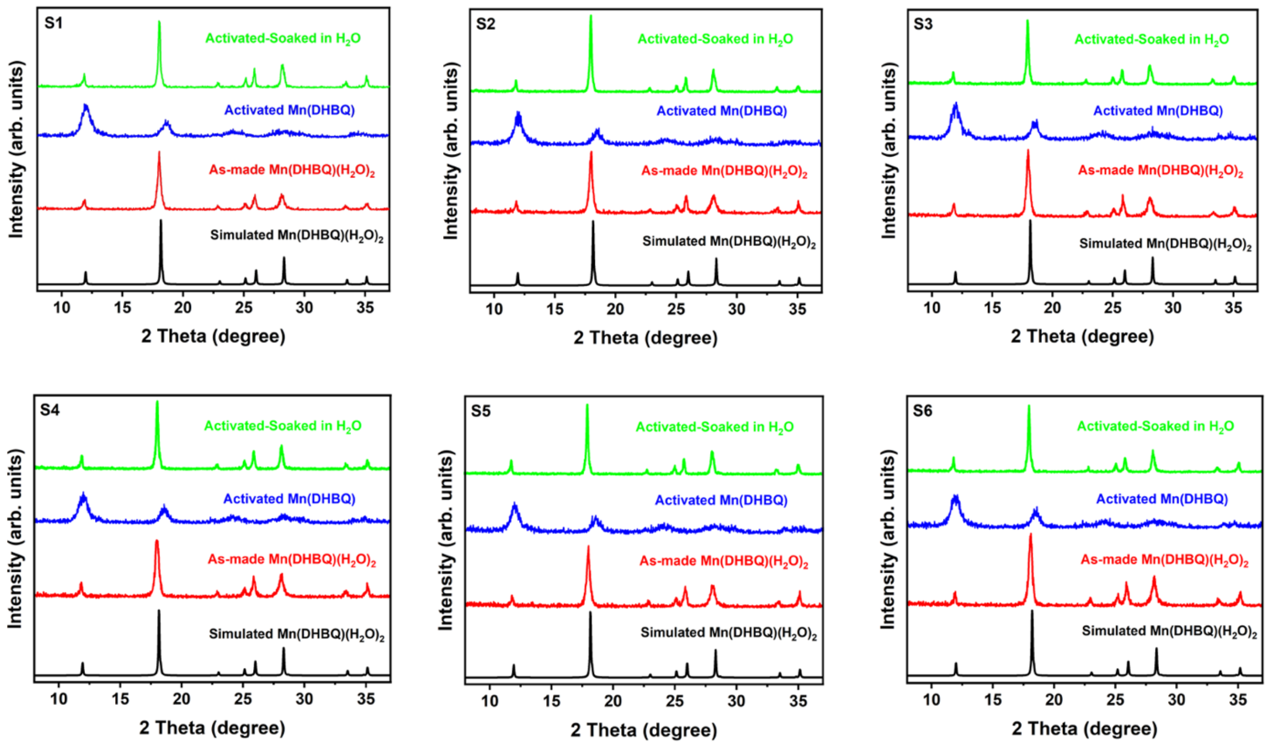

Supplementary Fig. 49. The PXRD patterns of Mn-DHBQ samples from six batches of parallel synthesis.

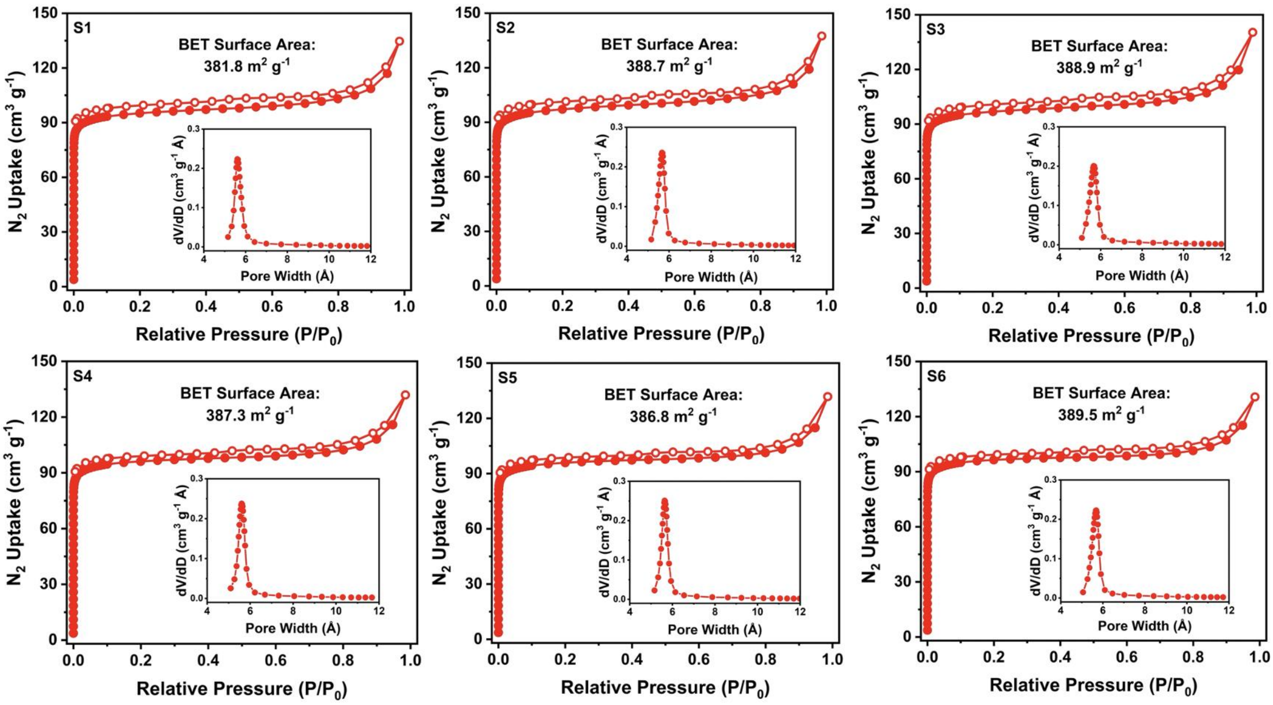

Supplementary Fig. 50. The N_2 sorption isotherms at 77 K and pore size distribution of Mn-DHBQ samples from six batches of parallel synthesis.

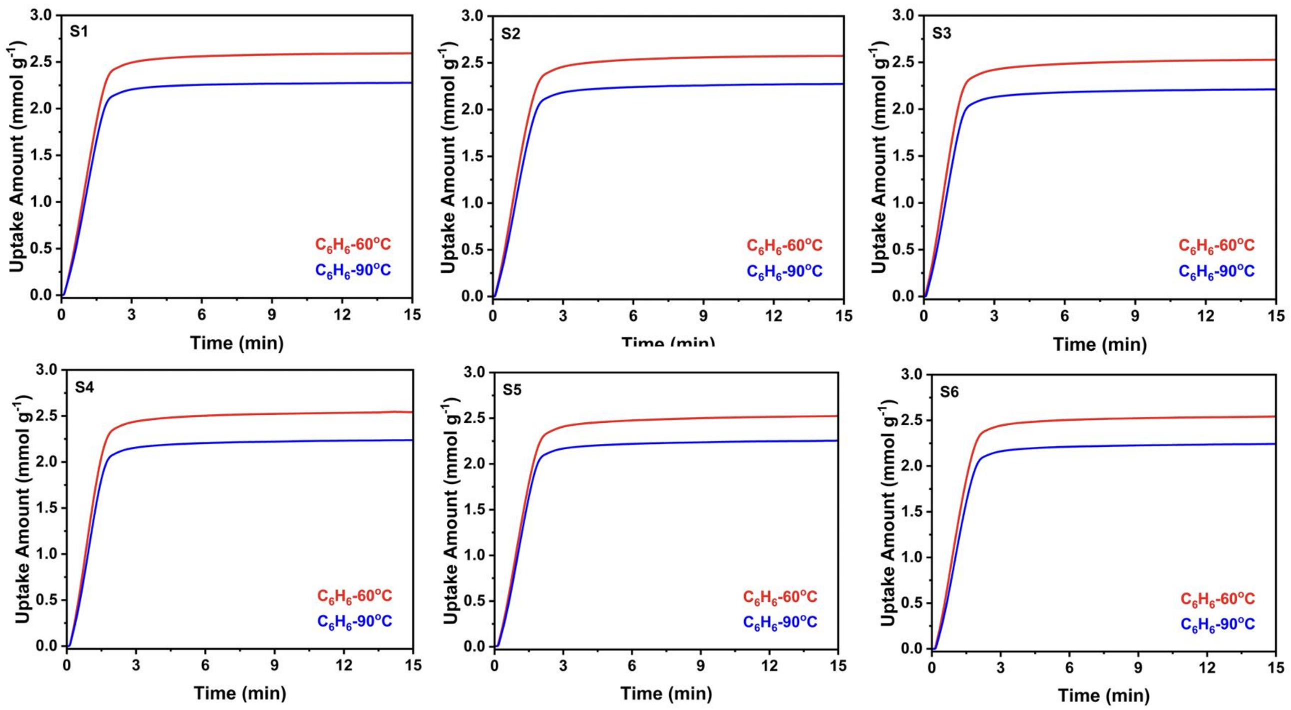

Supplementary Fig. 51. The dynamic C_6H_6 adsorption isotherms of Mn-DHBQ samples (six batches) collected at different temperatures (60 and 90 °C).

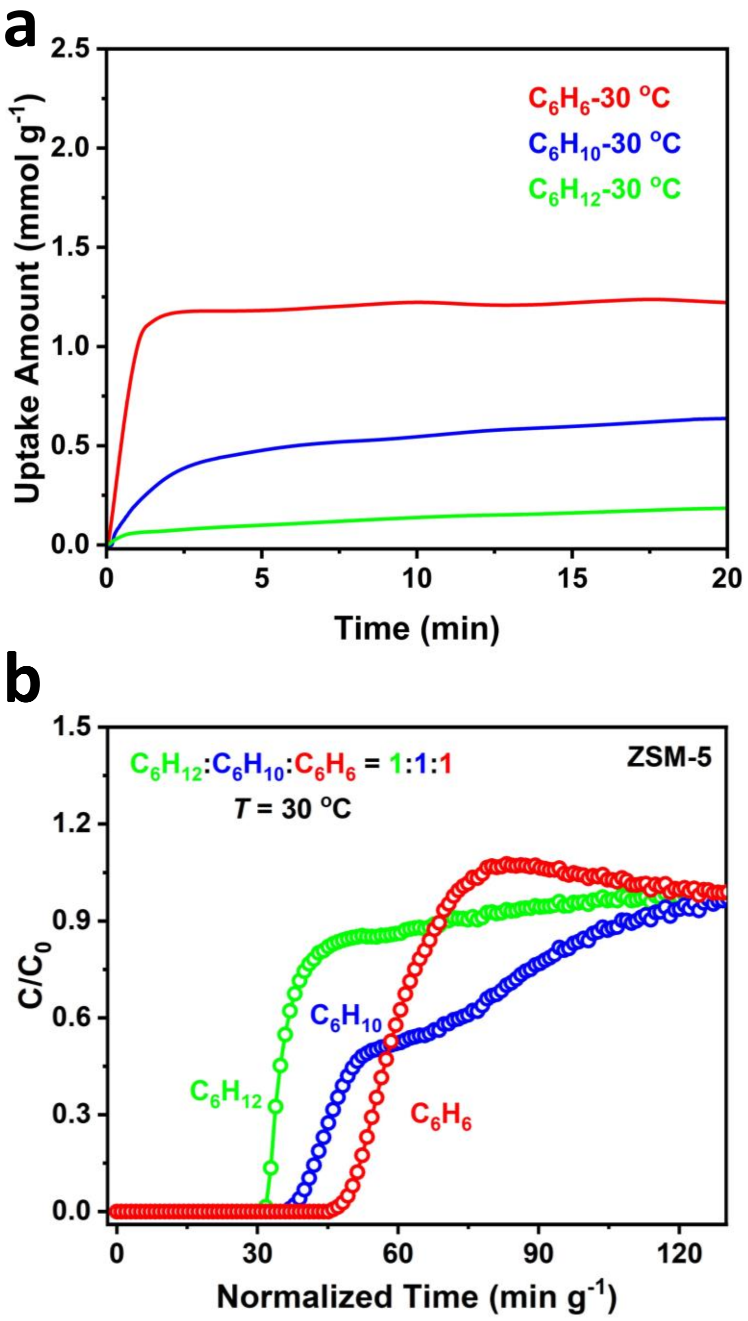

Supplementary Fig. 52. (**a**) Dynamic adsorption isotherms of C6 cyclic hydrocarbons on ZSM-5 at 30 °C; (**b**) Breakthrough curves of an equimolar ternary C_6H_6/C_6H_{10}/C_6H_{12} mixture on a ZSM-5 sample packed in the single column at 30 °C.

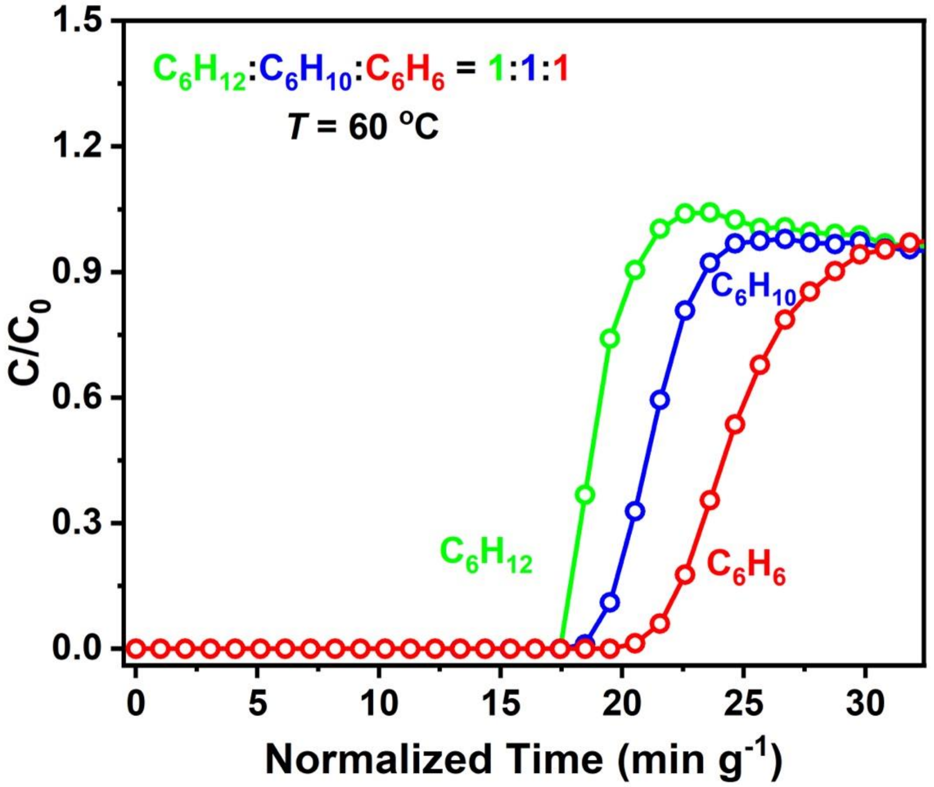

Supplementary Fig. 53. Breakthrough curves of an equimolar ternary C_6H_6/C_6H_{10}/C_6H_{12} mixture on ZSM-5 sample packed in the single column at 60 °C.

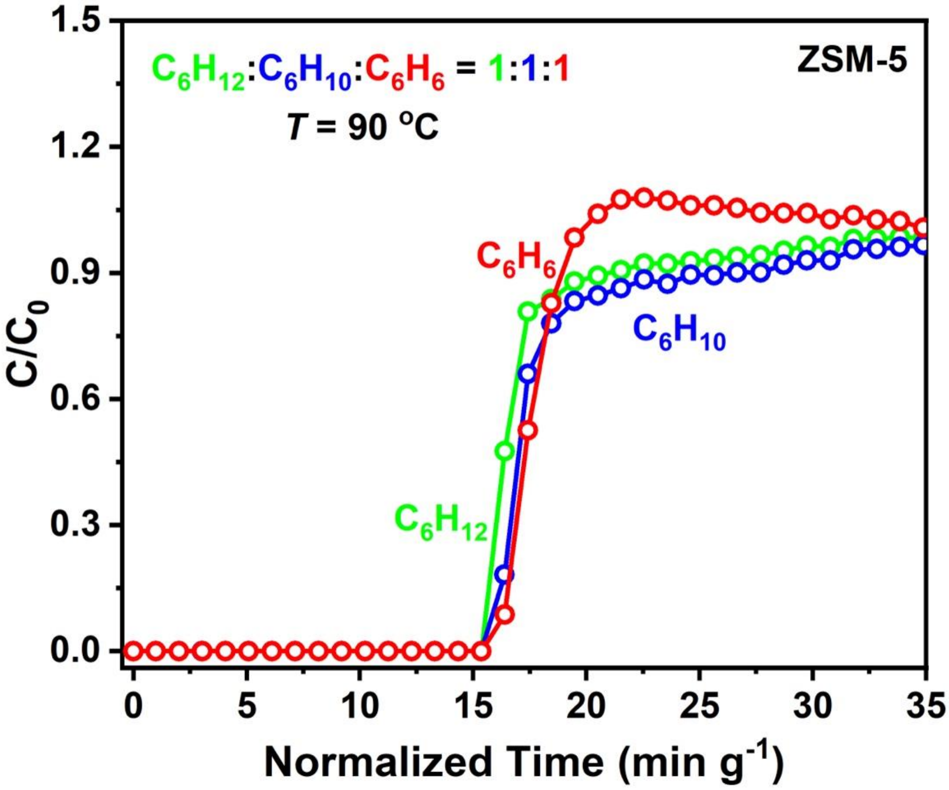

Supplementary Fig. 54. Breakthrough curves of an equimolar ternary $C_6H_6/C_6H_{10}/C_6H_{12}$ mixture on ZSM-5 sample packed in the single column at 90 °C.

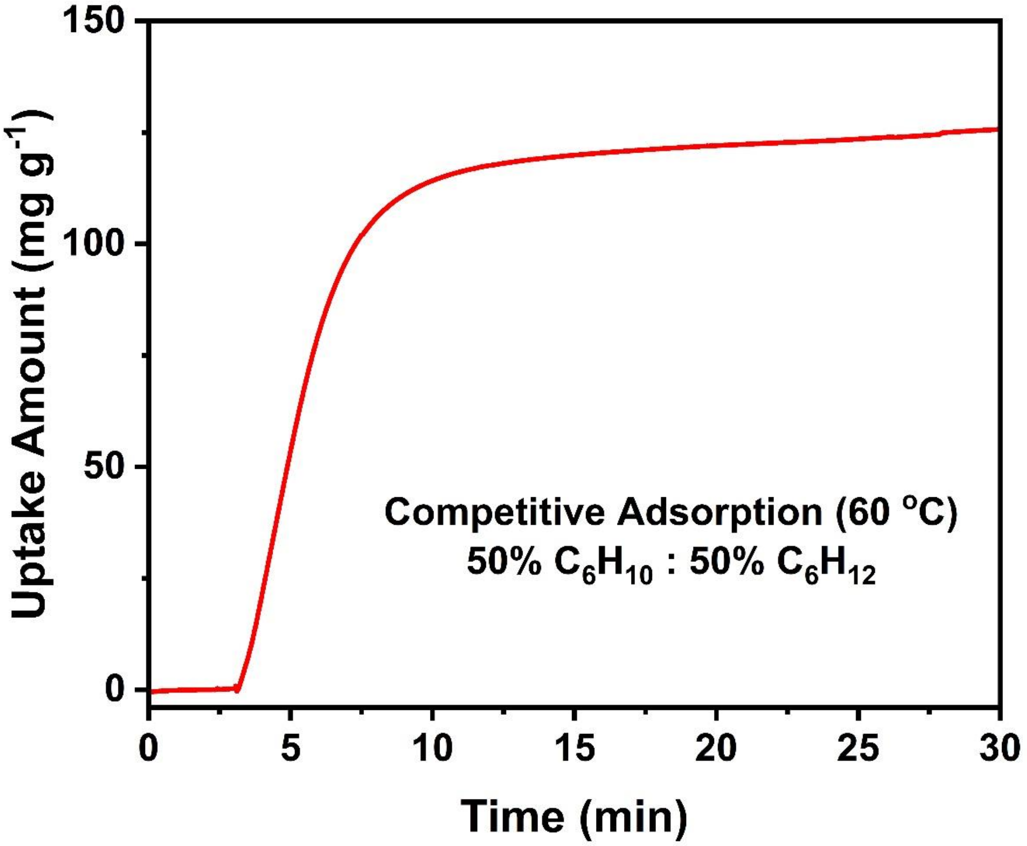

Supplementary Fig. 55. The dynamic competitive adsorption curve of equimolar binary mixture of cyclohexene and cyclohexane vapors on Mn-DHBQ at 60 °C.

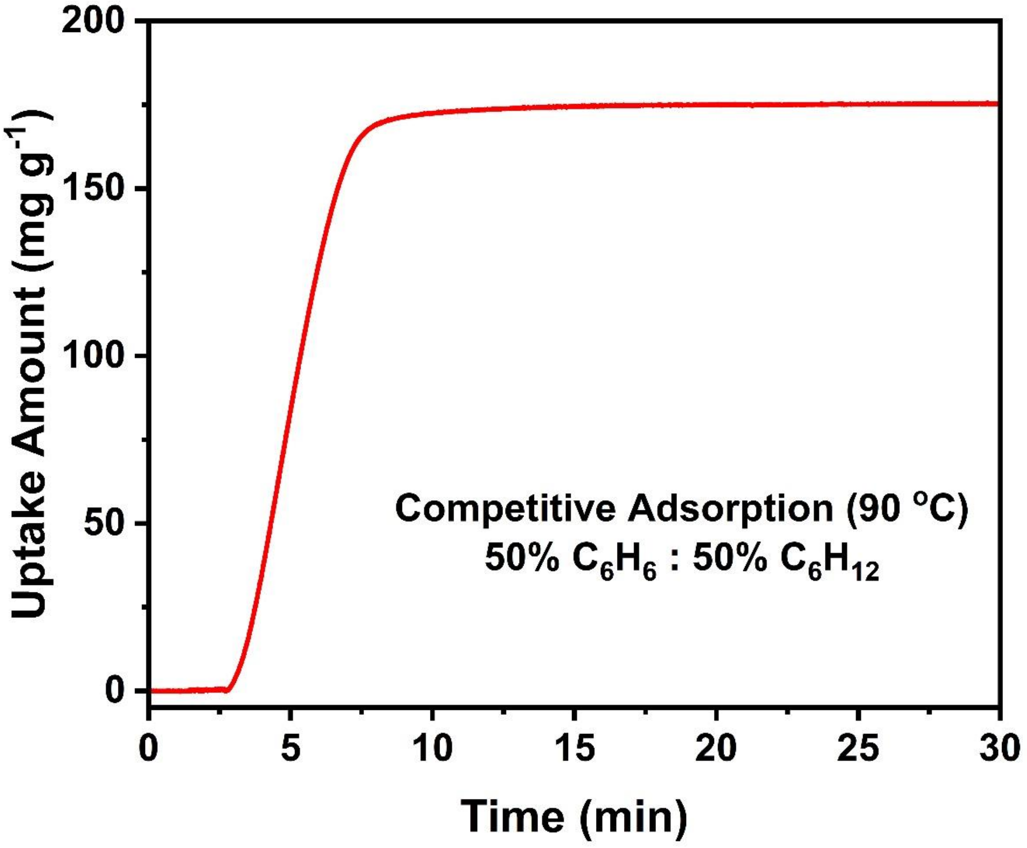

Supplementary Fig. 56. The dynamic competitive adsorption curve of equimolar binary mixture of benzene and cyclohexane vapors on Mn-DHBQ at 90 °C.

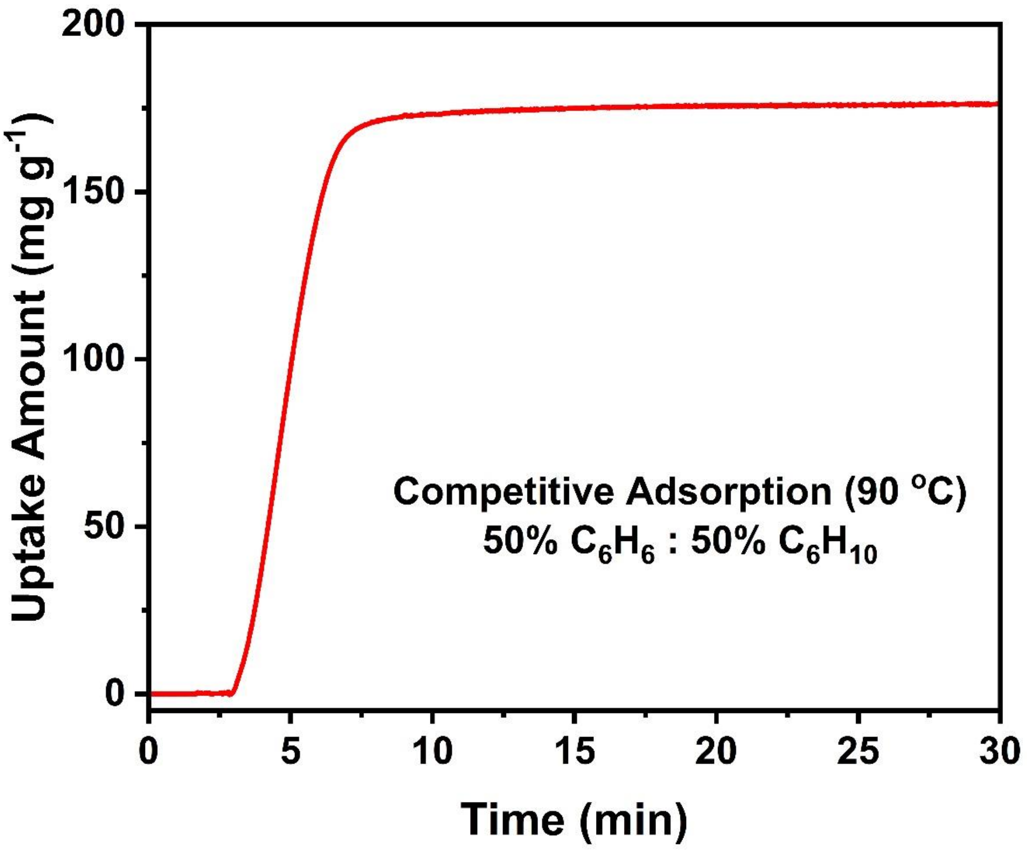

Supplementary Fig. 57. The dynamic competitive adsorption curve of equimolar binary mixture of benzene and cyclohexene vapors on Mn-DHBQ at 90 °C.

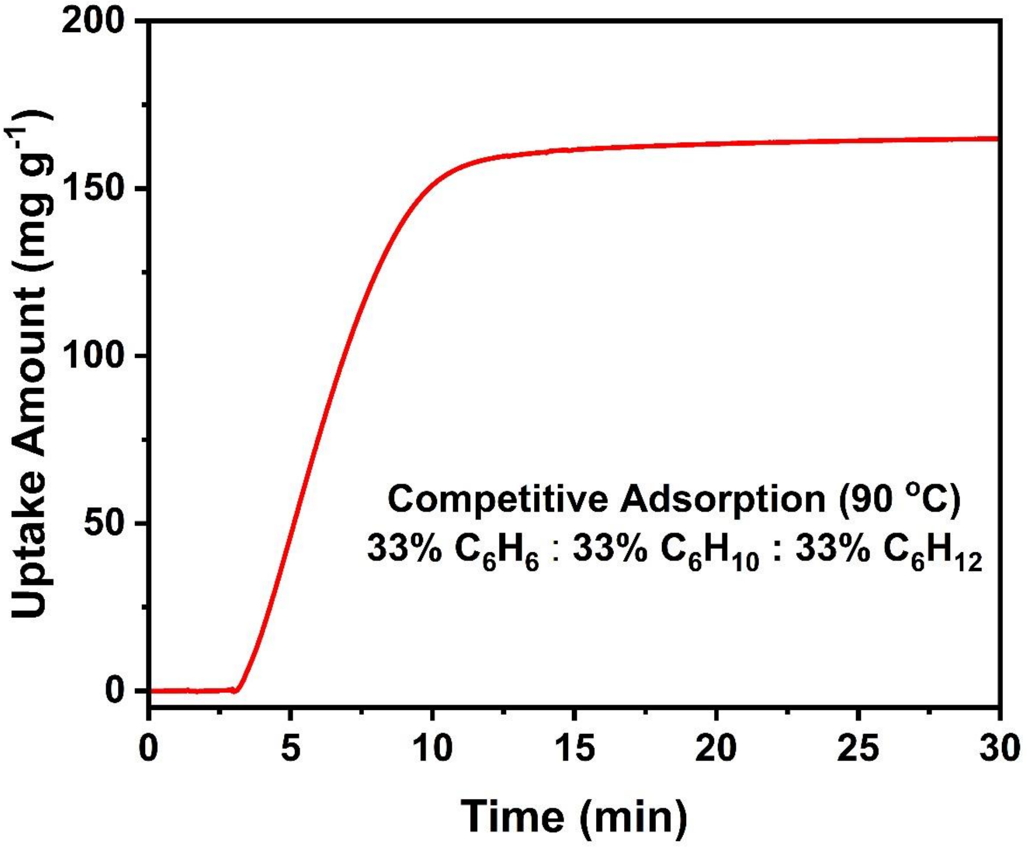

Supplementary Fig. 58. The dynamic competitive adsorption curve of equimolar ternary mixture of benzene, cyclohexene and cyclohexane vapors on Mn-DHBQ at 90 °C.

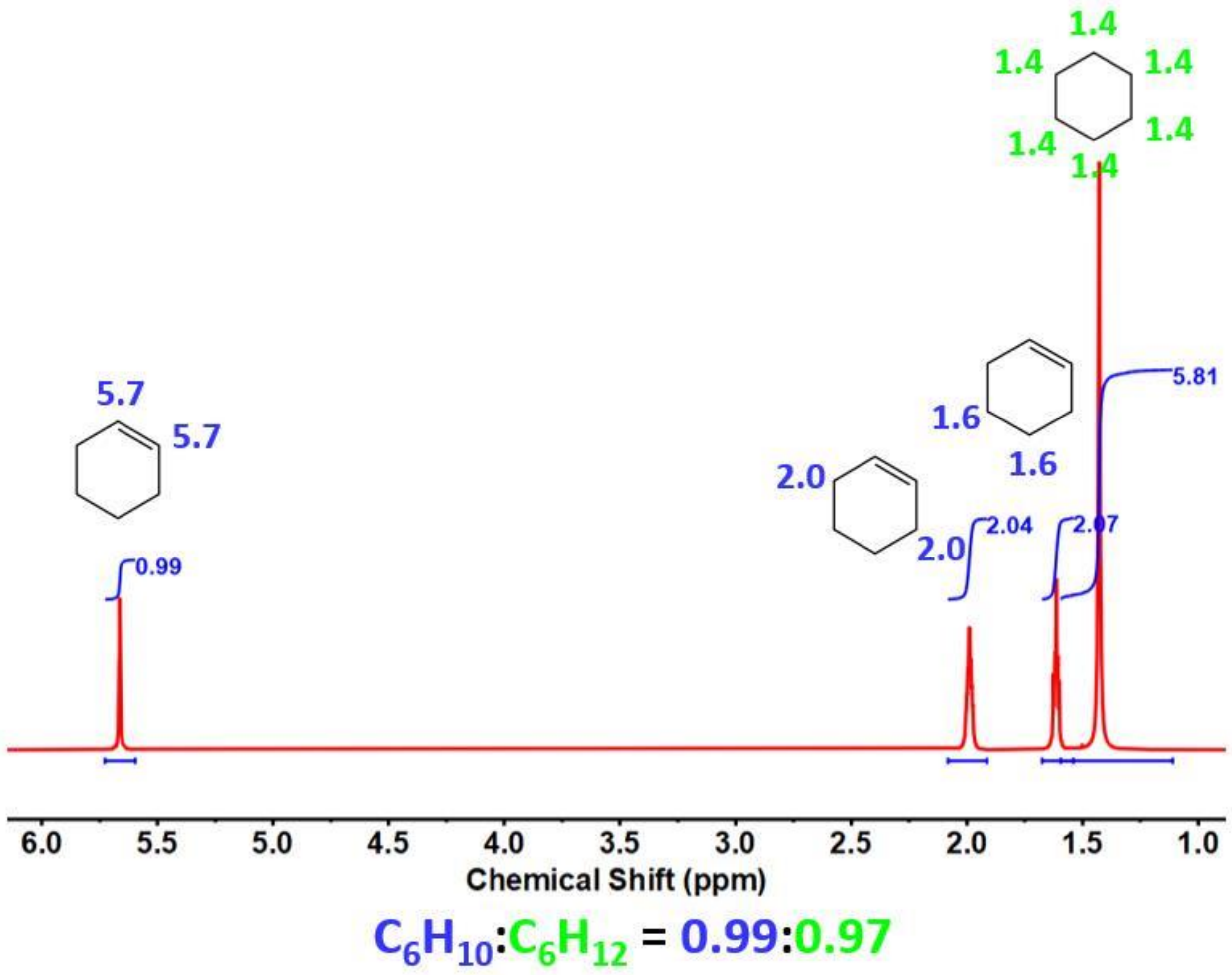

Supplementary Fig. 59. Magnified ^{1}H NMR spectrum for the standard equimolar cyclohexene-cyclohexane binary mixture.

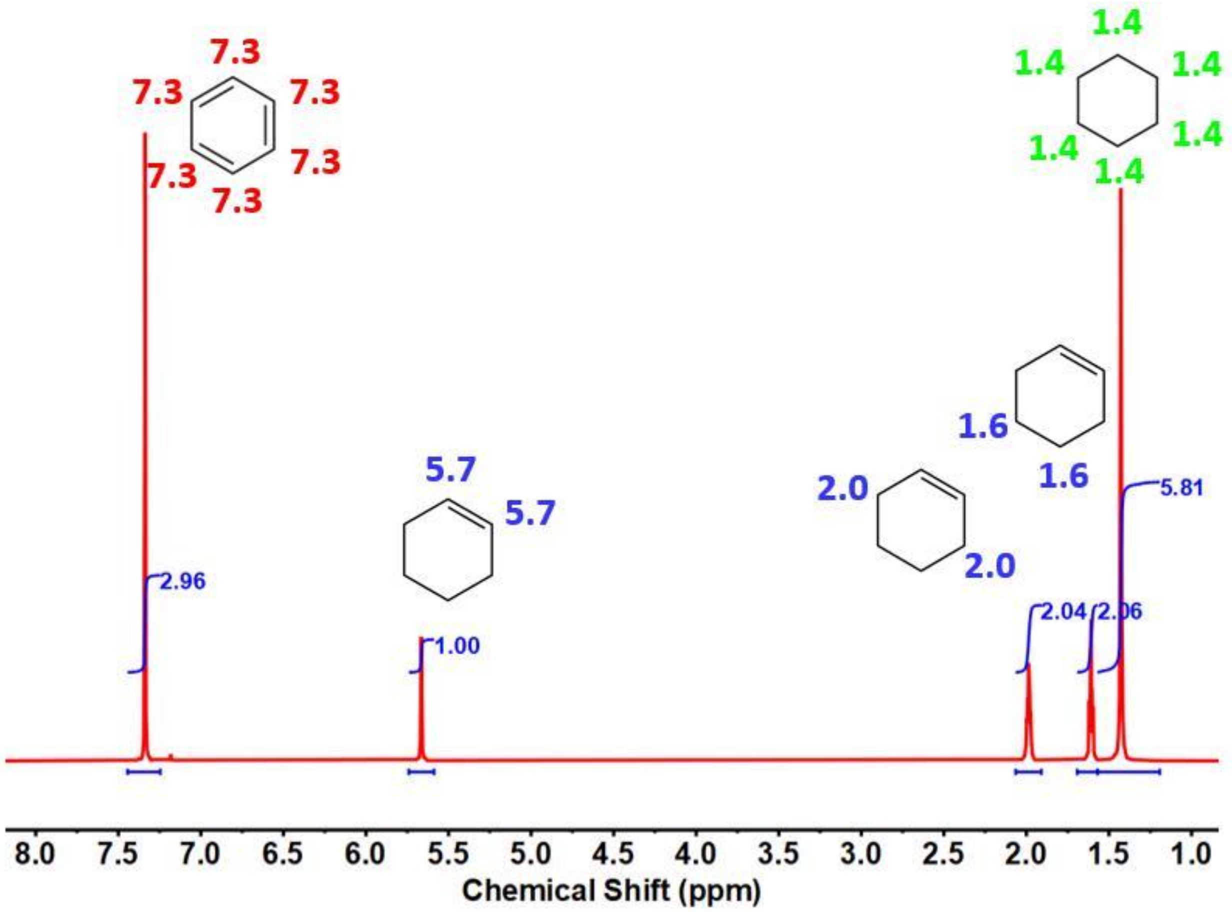

C_6H_6:C_6H_{10}:C_6H_{12} = 0.99:1.00:0.97

Supplementary Fig. 60. Magnified ^{1}H NMR spectrum for the standard equimolar benzene-cyclohexene-cyclohexane ternary mixture.

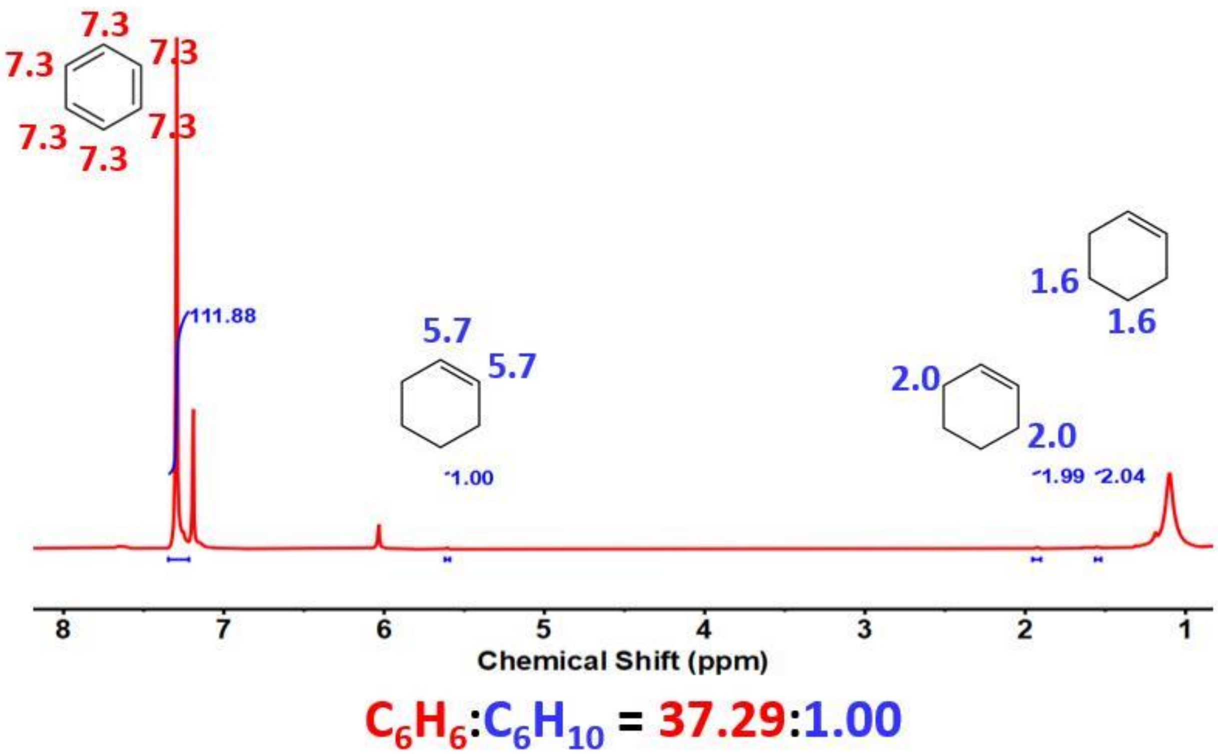

Supplementary Fig. 61. Magnified ^{1}H NMR spectrum for the dynamic competitive adsorption of equimolar binary mixture of benzene and cyclohexene vapors on Mn-DHBQ at 90 °C. The vapor-adsorbed Mn-DHBQ samples were decomposed by 2 mL of concentrated HCl solution and then exchanged by 2 mL of $CDCl_3$.

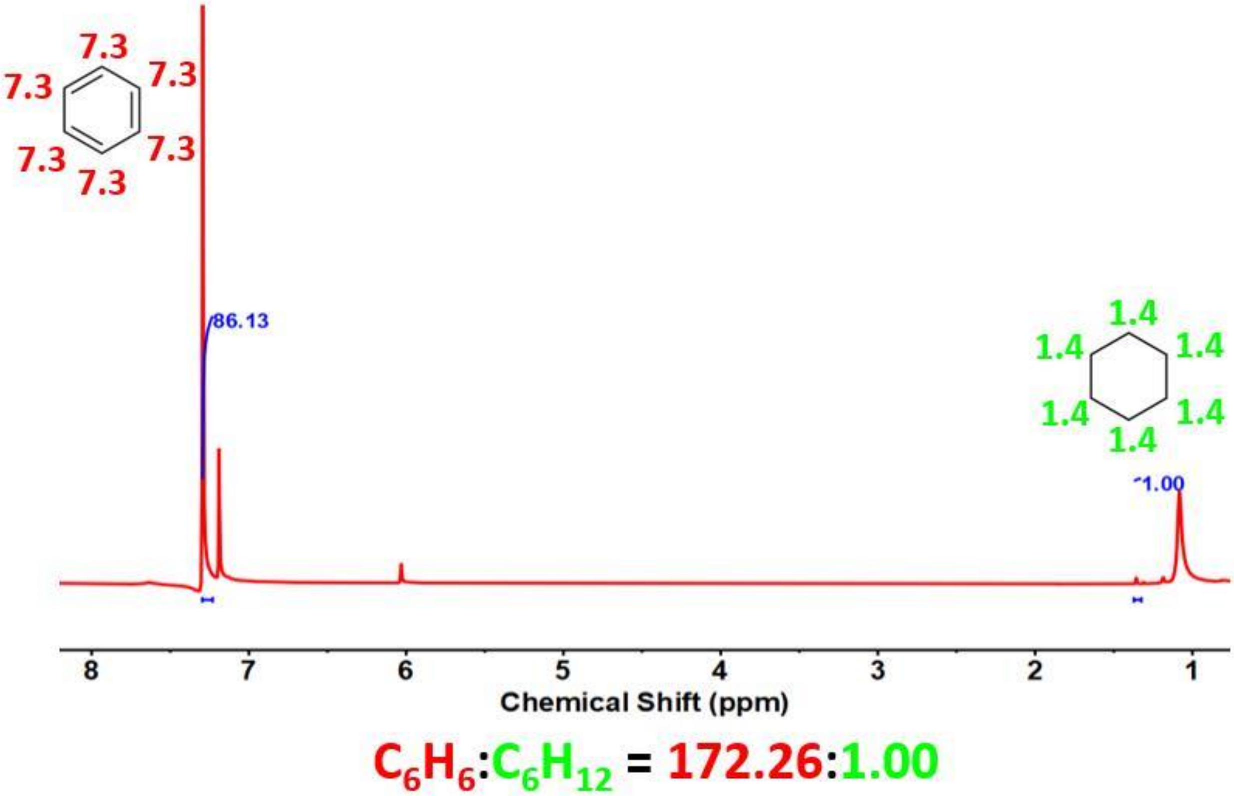

Supplementary Fig. 62. Magnified ^{1}H NMR spectrum for the dynamic competitive adsorption of equimolar binary mixture of benzene and cyclohexane vapors on Mn-DHBQ at 90 °C. The vapor-adsorbed Mn-DHBQ samples were decomposed by 2 mL of concentrated HCl solution and then exchanged by 2 mL of $CDCl_3$.

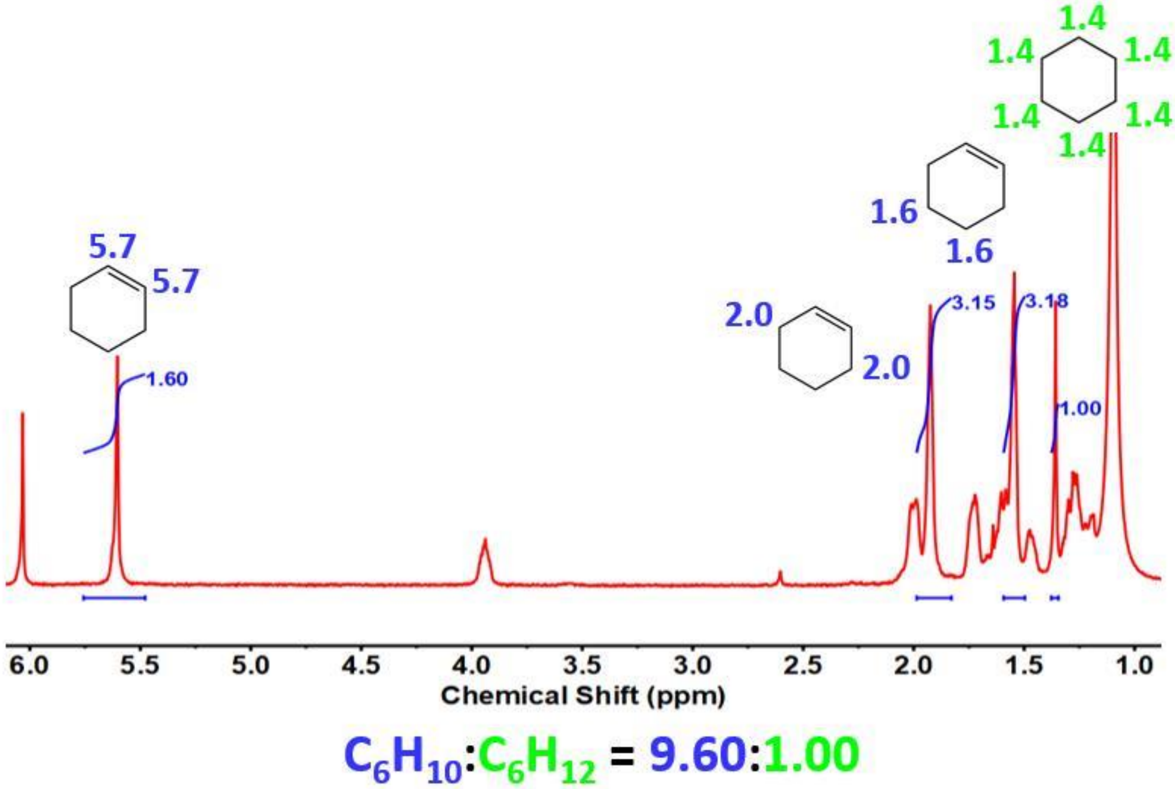

Supplementary Fig. 63. Magnified ^{1}H NMR spectrum for the dynamic vapor competitive adsorption of equimolar binary mixture of cyclohexene and cyclohexane vapors on Mn-DHBQ at 60 °C. The vapor-adsorbed Mn-DHBQ samples were decomposed by 2 mL of concentrated HCl solution and then exchanged by 2 mL of $CDCl_3$.

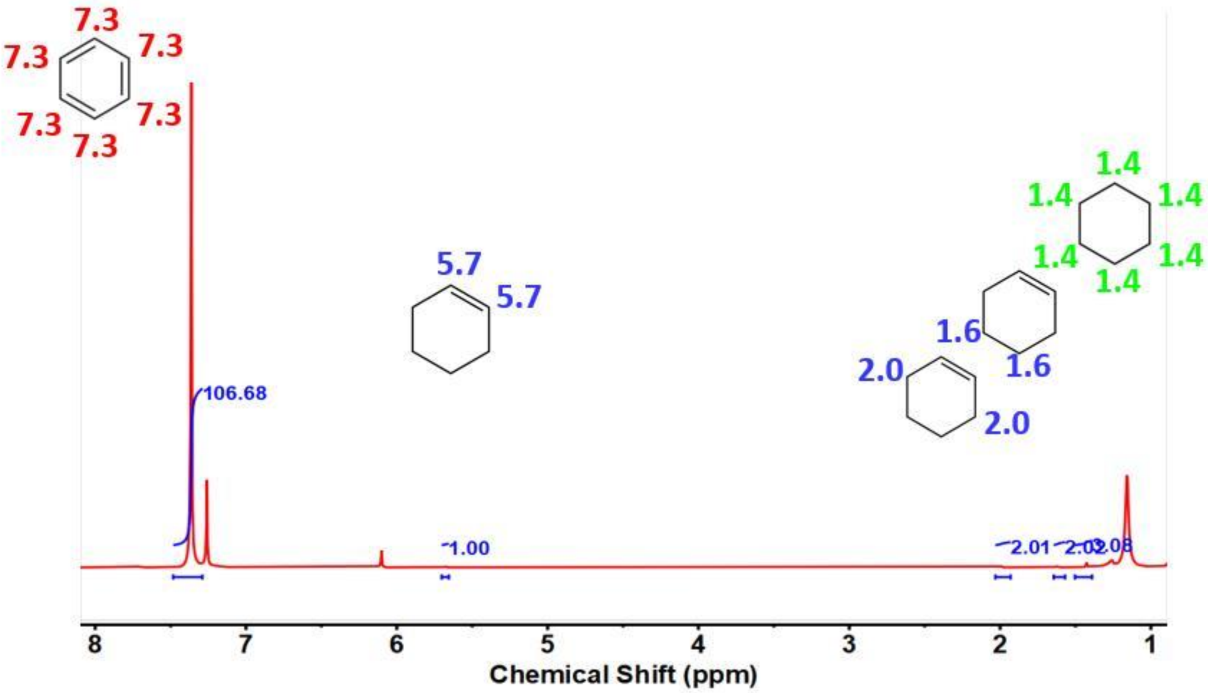

C_6H_6:C_6H_{10}:C_6H_{12} = 35.56:1.00:0.33

Supplementary Fig. 64. Magnified ^{1}H NMR spectrum for the dynamic competitive adsorption of equimolar ternary mixture of benzene, cyclohexene and cyclohexane vapors on Mn-DHBQ at 90 °C. The vapor-adsorbed Mn-DHBQ samples were decomposed by 2 mL of concentrated HCl solution and then exchanged by 2 mL of $CDCl_3$.

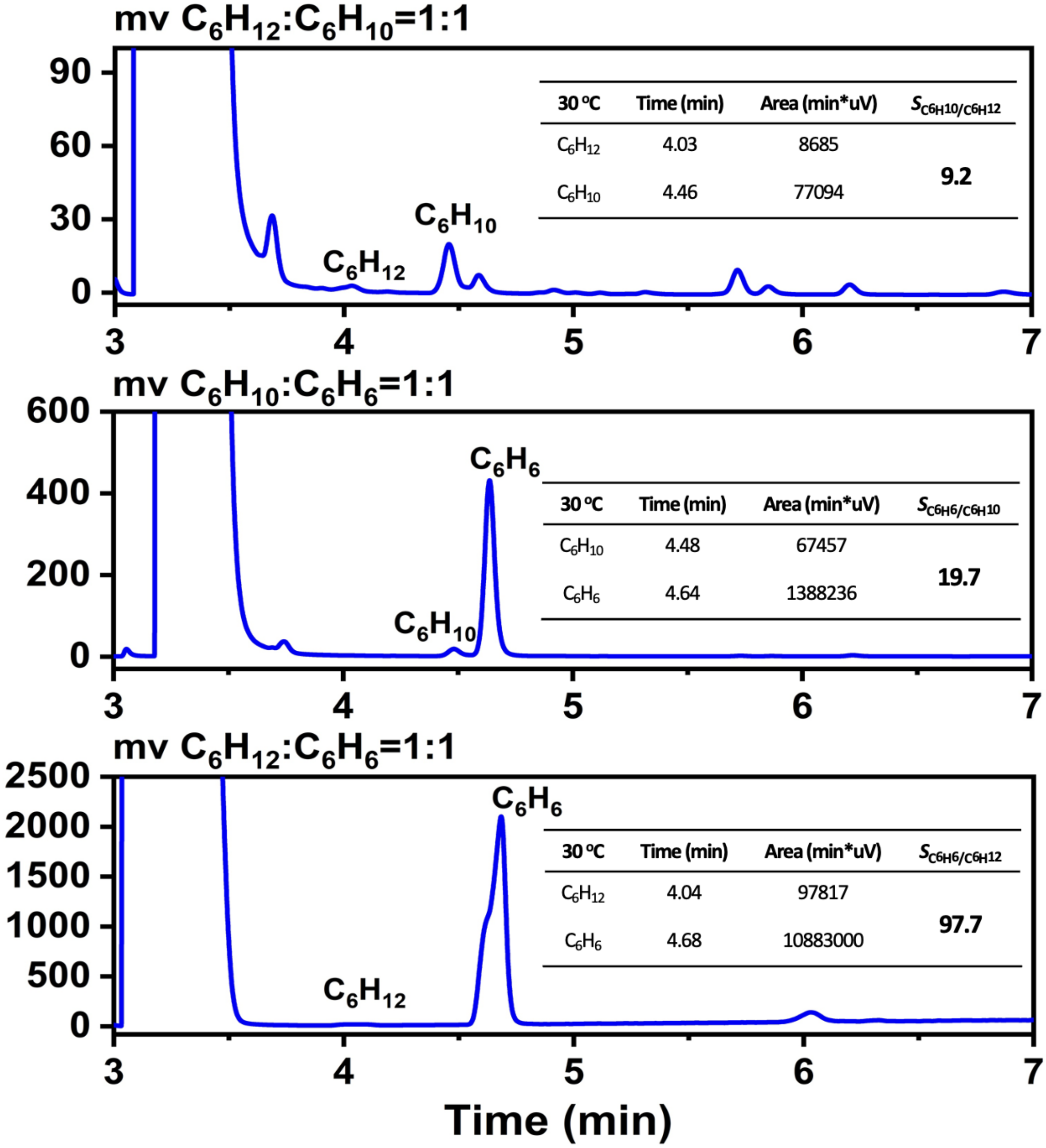

Supplementary Fig. 65. GC plots and selectivity coefficients for the static liquid competitive adsorption of equimolar binary mixtures of cyclohexane-cyclohexene (top), cyclohexene-benzene (middle), and cyclohexane-benzene (bottom) on Mn-DHBQ at 30 °C. The liquid-adsorbed Mn-DHBQ samples were decomposed by 2 mL of concentrated HCl solution and then exchanged by 2 mL of $CDCl_2$.

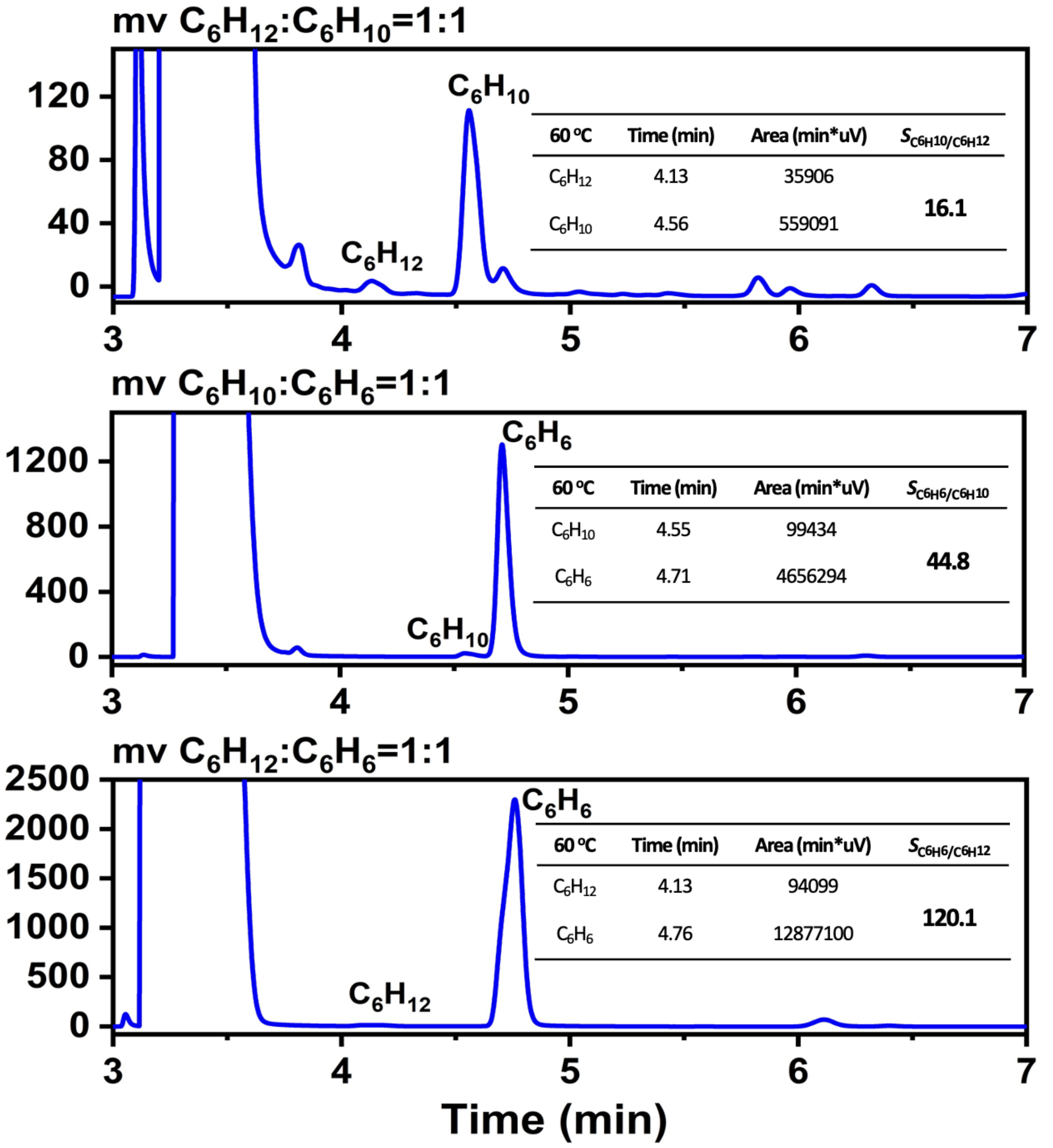

Supplementary Fig. 66. GC plots and the selectivity coefficients for the static liquid competitive adsorption of equimolar binary mixtures of cyclohexane-cyclohexene (top), cyclohexene-benzene (middle), and cyclohexane-benzene (bottom) on Mn-DHBQ at 60 °C. The liquid-adsorbed Mn-DHBQ samples were decomposed by 2 mL of concentrated HCl solution and then exchanged by 2 mL of $CDCl_2$.

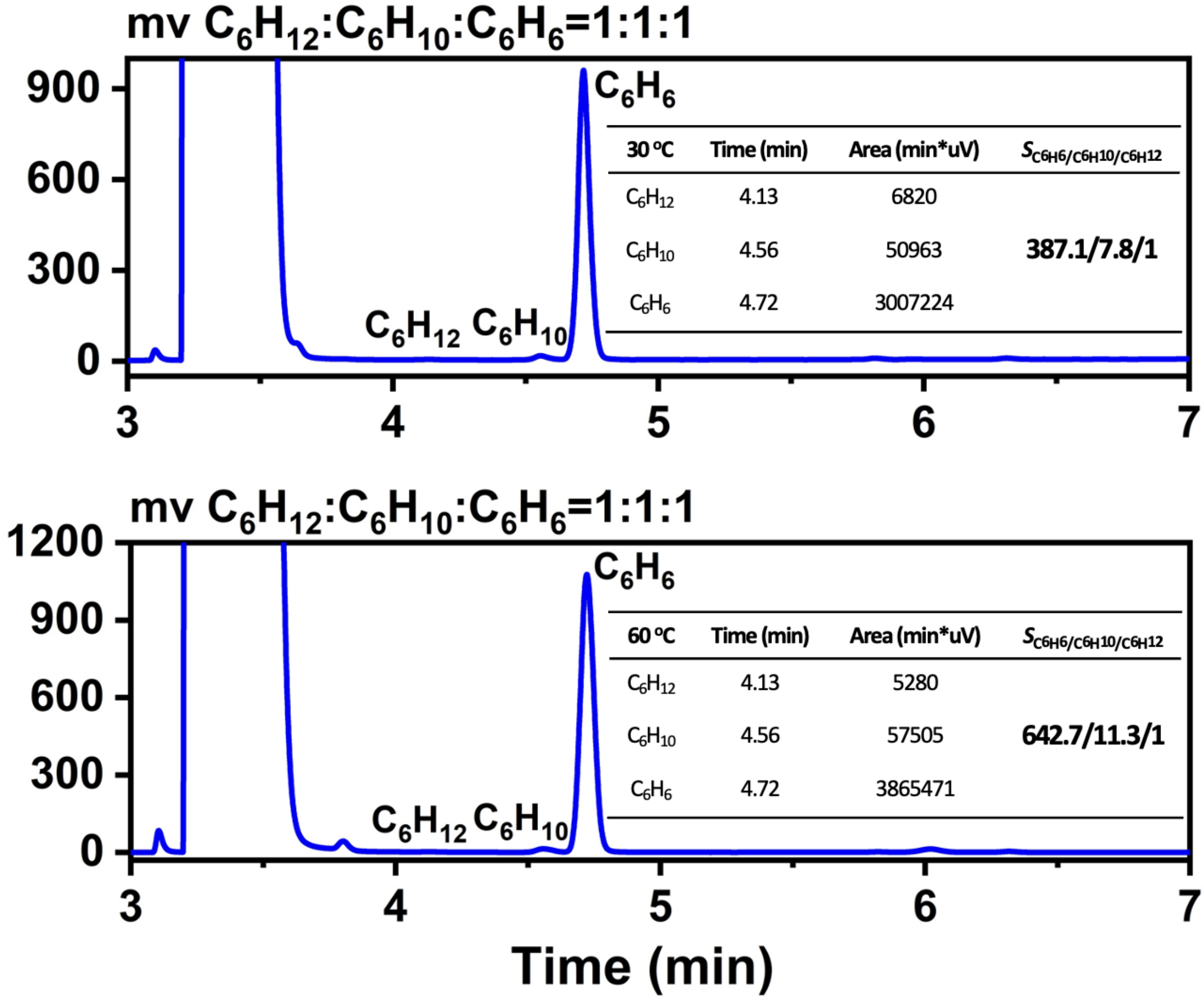

Supplementary Fig. 67. GC plots and the selectivity coefficients for the static liquid competitive adsorption of equimolar ternary mixtures of cyclohexane-cyclohexene-benzene on Mn-DHBQ at 30 °C (top) and 60 °C (bottom). The liquid-adsorbed Mn-DHBQ samples were decomposed by 2 mL of concentrated HCl solution and then exchanged by 2 mL of $CDCl_2$.

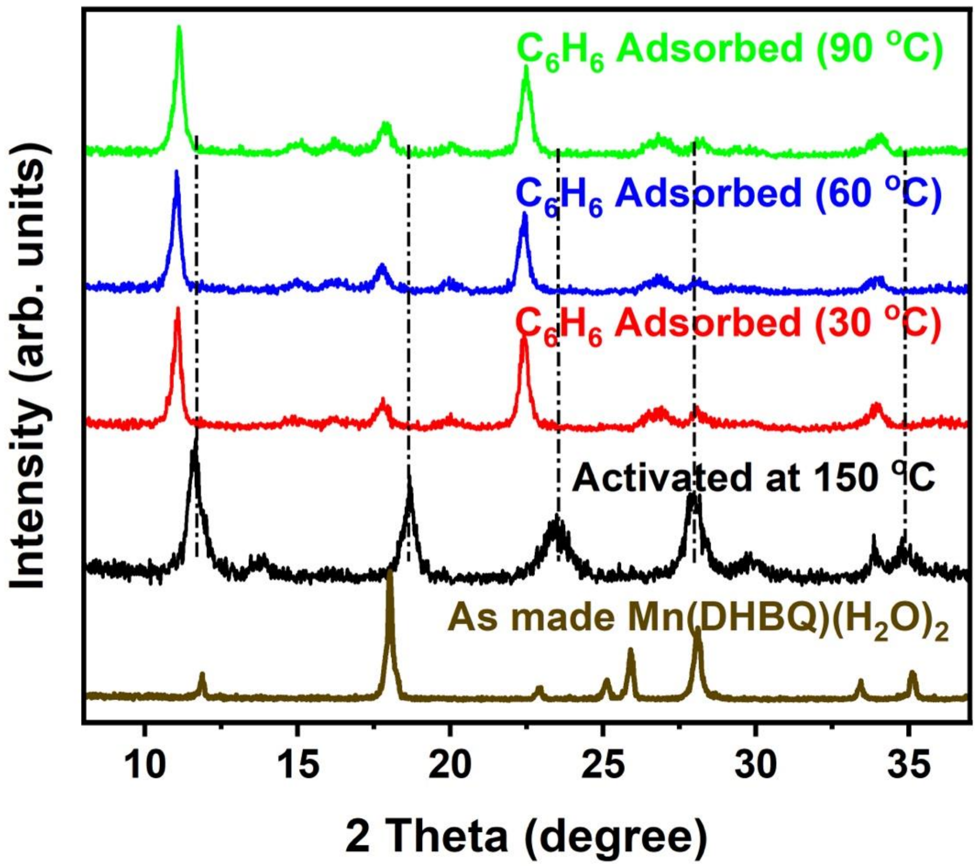

Supplementary Fig. 68. The PXRD patterns of benzene-adsorbed Mn-DHBQ samples at 30, 60 and 90 °C.

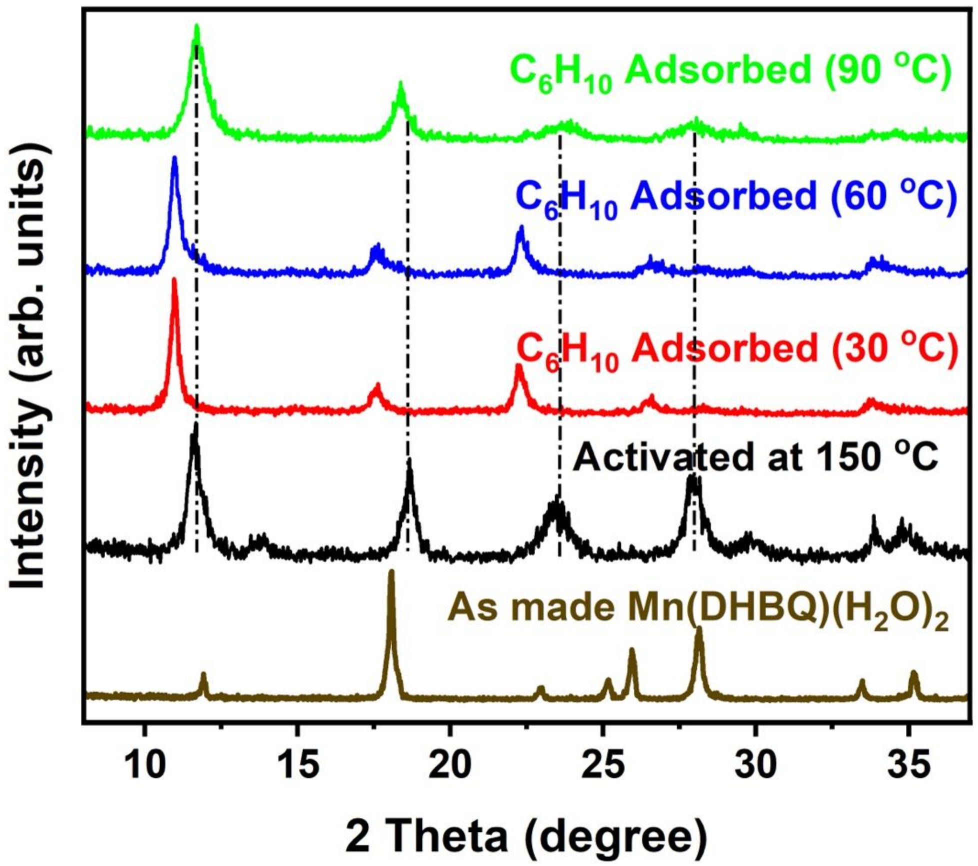

Supplementary Fig. 69. The PXRD patterns of cyclohexene-adsorbed Mn-DHBQ samples at 30, 60 and 90 °C.

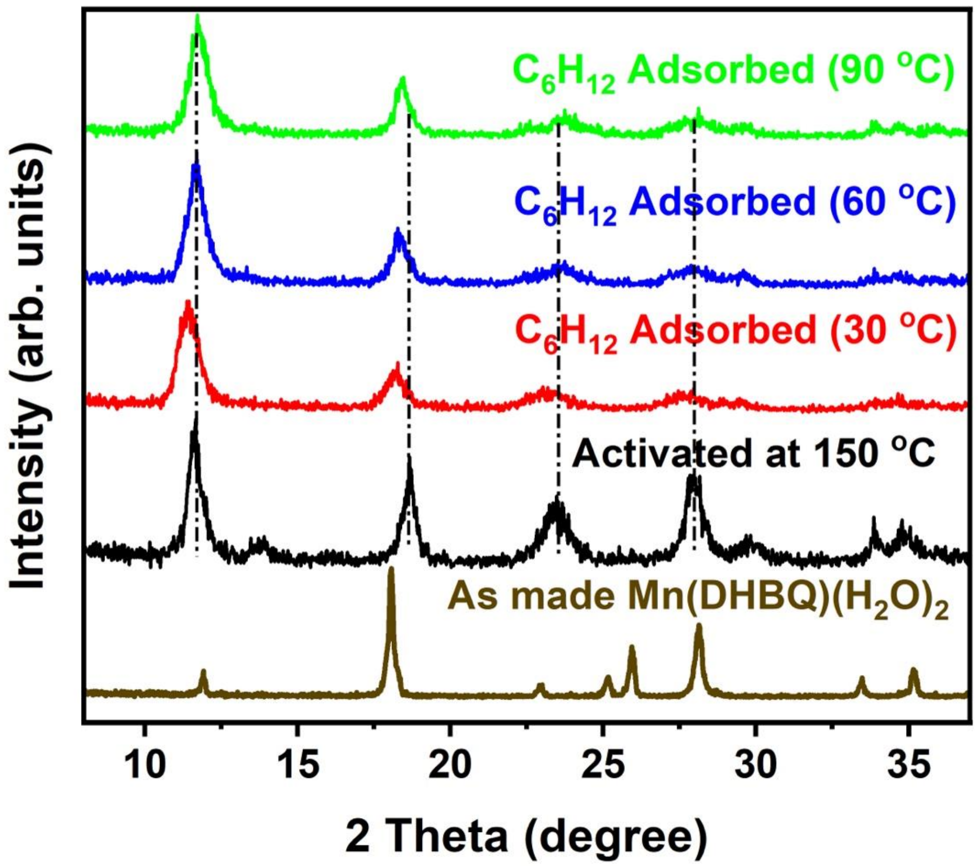

Supplementary Fig. 70. The PXRD patterns of cyclohexane-adsorbed Mn-DHBQ samples at 30, 60 and 90 °C.

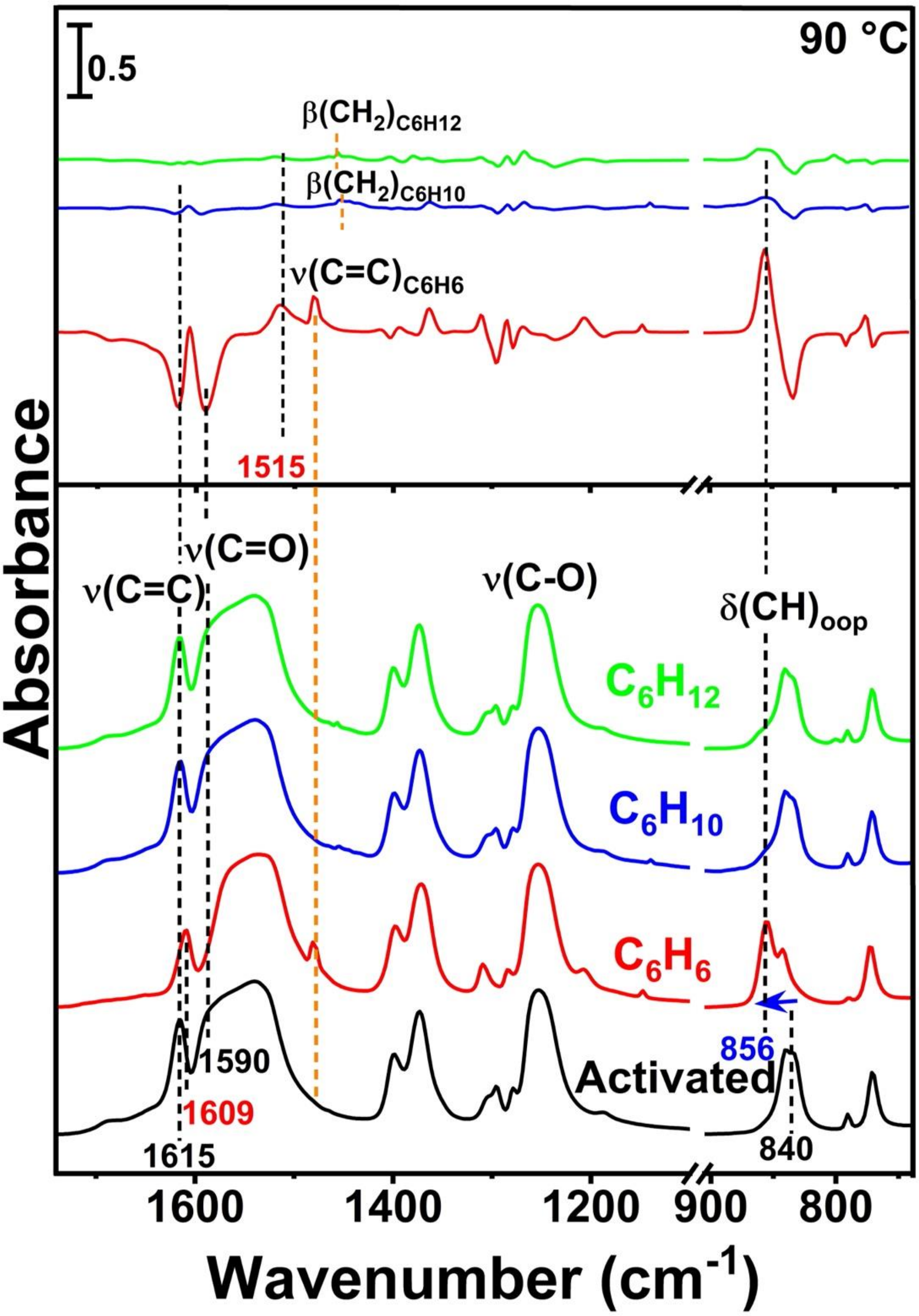

Supplementary Fig. 71. *In situ* IR spectra of hydrocarbon-loaded Mn-DHBQ at 90 °C.

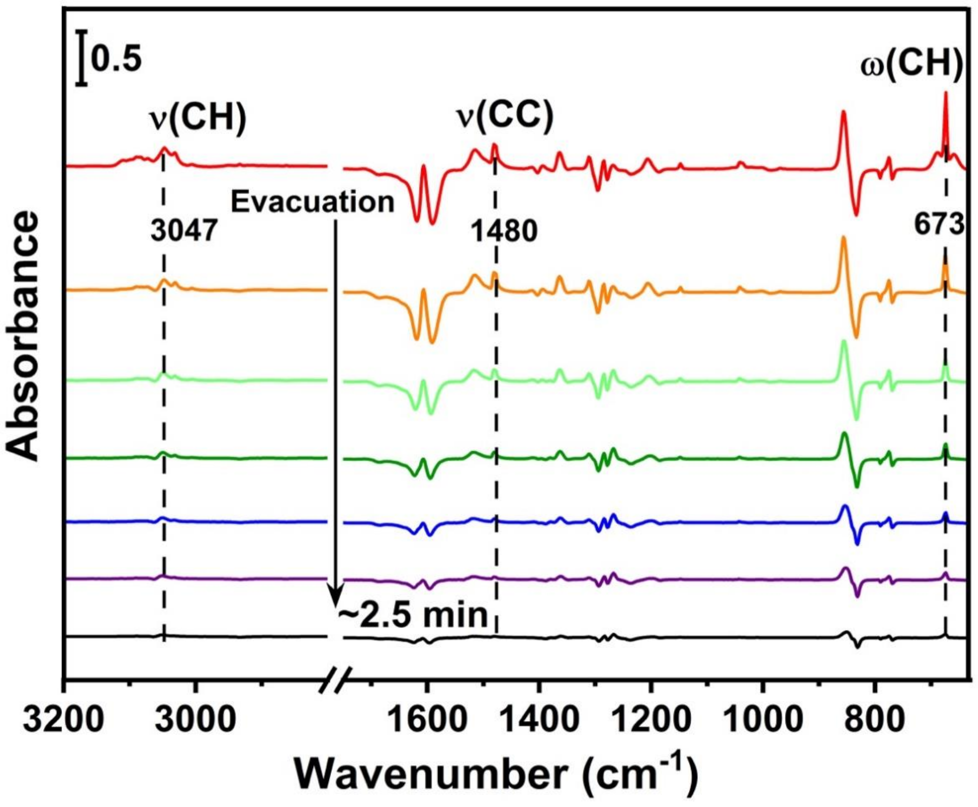

Supplementary Fig. 72. *In situ* IR spectra of benzene desorption on Mn-DHBQ under vacuum.

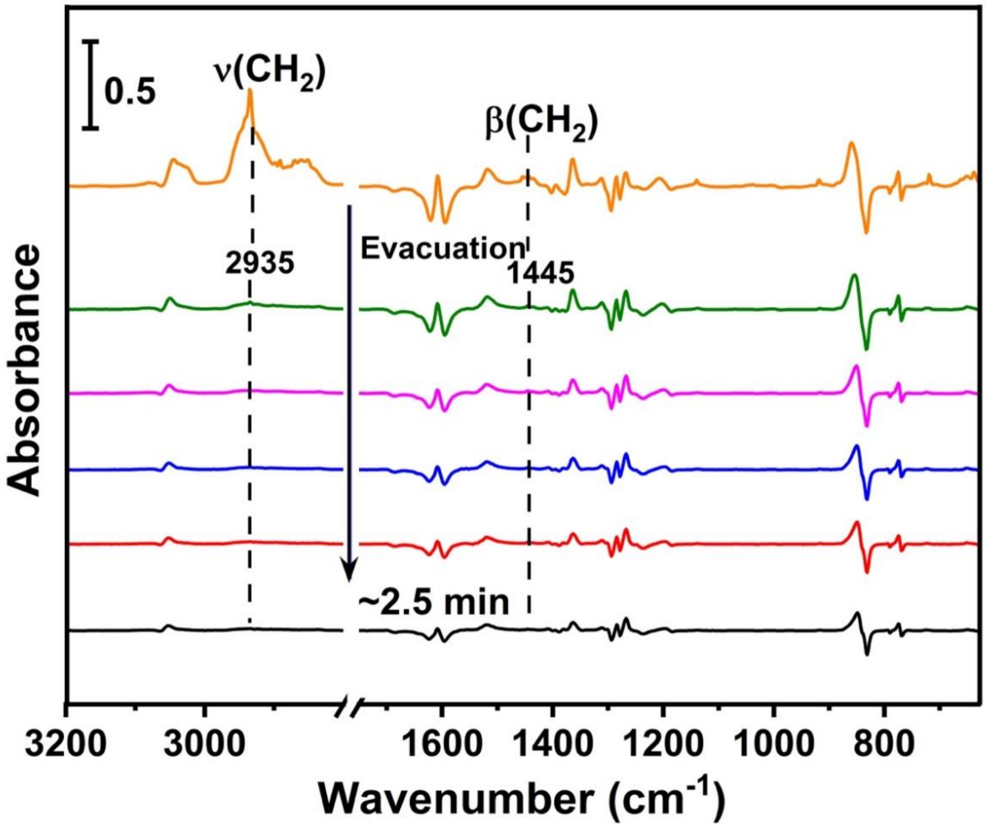

Supplementary Fig. 73. *In situ* IR spectra of cyclohexene desorption on Mn-DHBQ under vacuum.

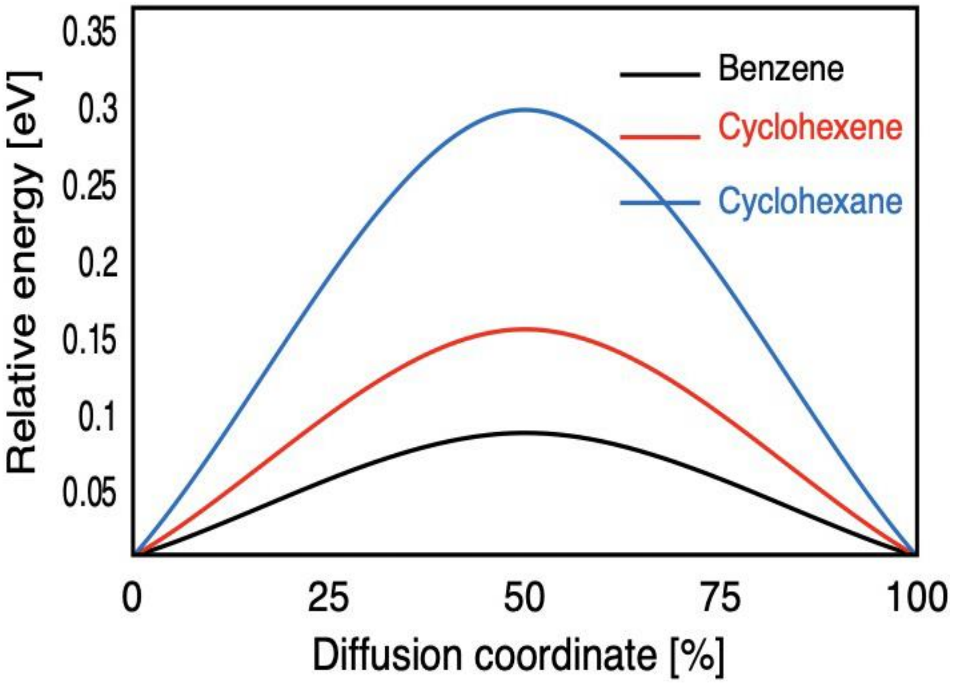

Supplementary Fig. 74. Diffusion barriers of the C6-cyclic hydrocarbons within Mn(DHBQ).

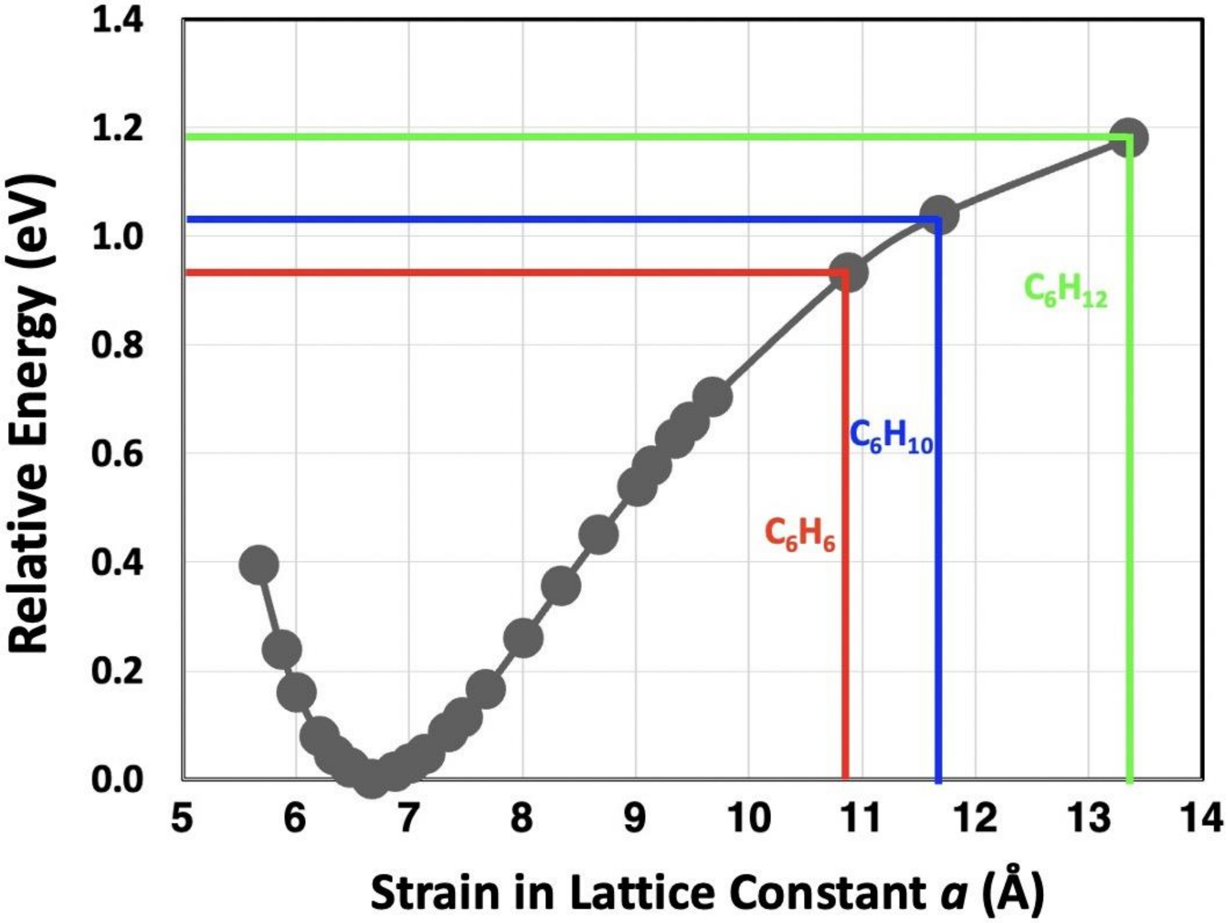

Supplementary Fig. 75. Energy cost of compressive and tensile strain along the crystallographic a-axis for a fully periodic Mn(DHBQ) (with water molecules removed). The three strains required to accommodate the three guest molecules are marked. Benzene requires the lowest stretching in comparison with the other two hydrocarbons and therefore requires the lowest energy for stretching. The framework is quite flexible and can be stretched to twice its lattice constant (*a*) at the cost of just ~1 eV.

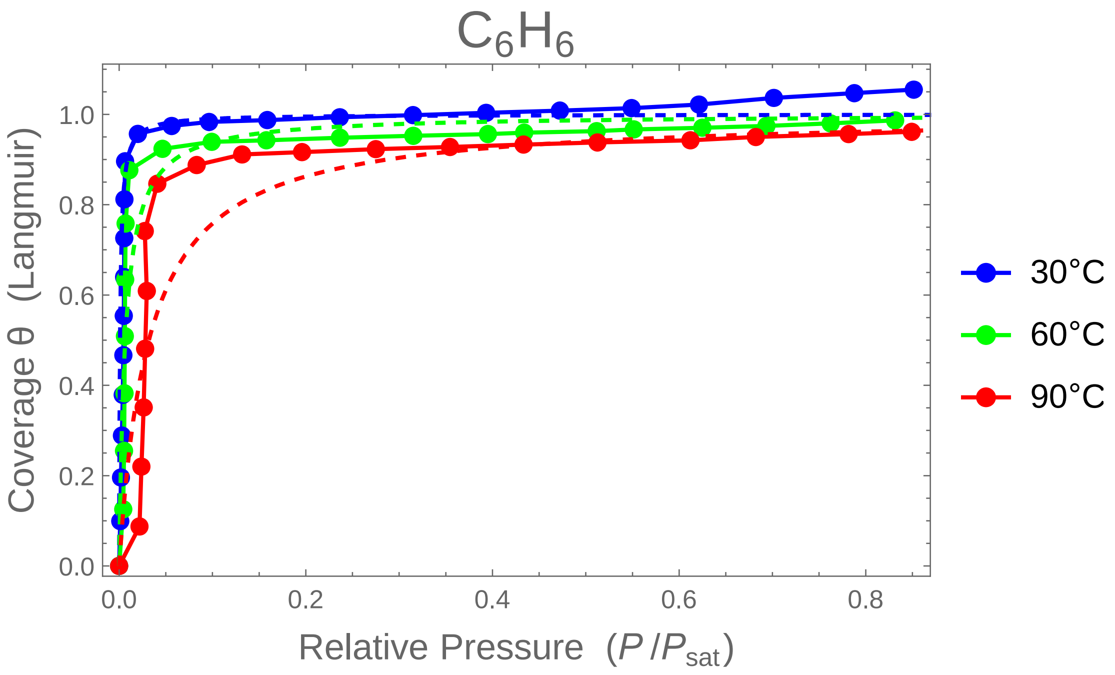

Supplementary Fig. 76: Experimental (solid-dotted) and simulated (dashed) isotherms for C_6H_6 as a function of temperature. The experimental line at 30 °C was mapped onto a Langmuir isotherm model to obtain the model parameters and serves as the reference. Assuming standard thermodynamics, the temperature dependence of the simulated 60 °C and 90 °C was then obtained through Van't Hoff's equation. Integrating the equation from 30 °C to 60 °C fields a change of the thermodynamic equilibrium constant by a factor of 6.6; integrating from 30 °C to 90 °C results in a factor of 31.9. The experimental 30 °C uptake for C_6H_6 is well captured by a standard Langmuir isotherm. The experimental 60 °C and 90 °C uptakes mostly follow the standard thermodynamics prediction, especially towards the saturation pressure. For low pressures, however, we see small deviations in that the experimental 60 °C and 90 °C loadings reach their saturation values somewhat faster than predicted by standard thermodynamics.

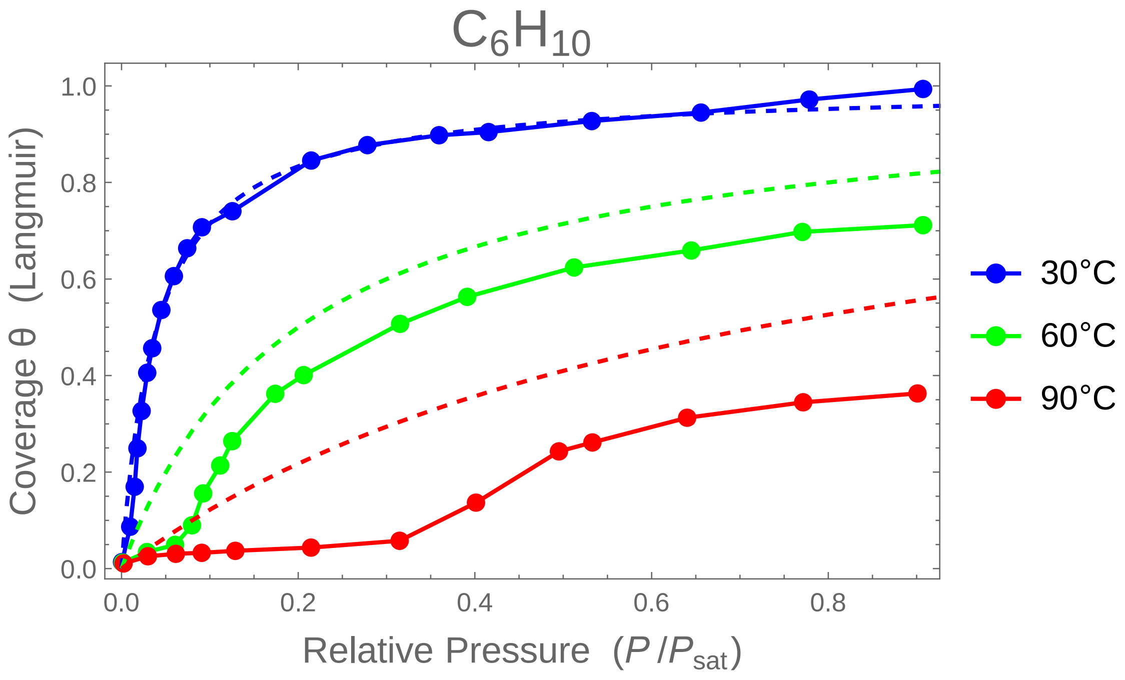

Supplementary Fig. 77: Experimental (solid-dotted) and simulated (dashed) isotherms for C_6H_{10} as a function of temperature. The experimental 30 °C uptake for C_6H_{10} is very well captured by a standard Langmuir isotherm. Going from 30 °C to 60 °C, integrating Van't Hoff's equation predicts that the thermodynamic equilibrium constant changes by a factor of 4.8; from 30 °C to 90 °C by a factor of 18.1. We find that the experimental uptake for 60 °C is below, in particular for very low pressures, the standard thermodynamics prediction. At 90 °C the experimental uptake deviates even more from the standard thermodynamics prediction, most drastically for relative pressures up to 0.4.

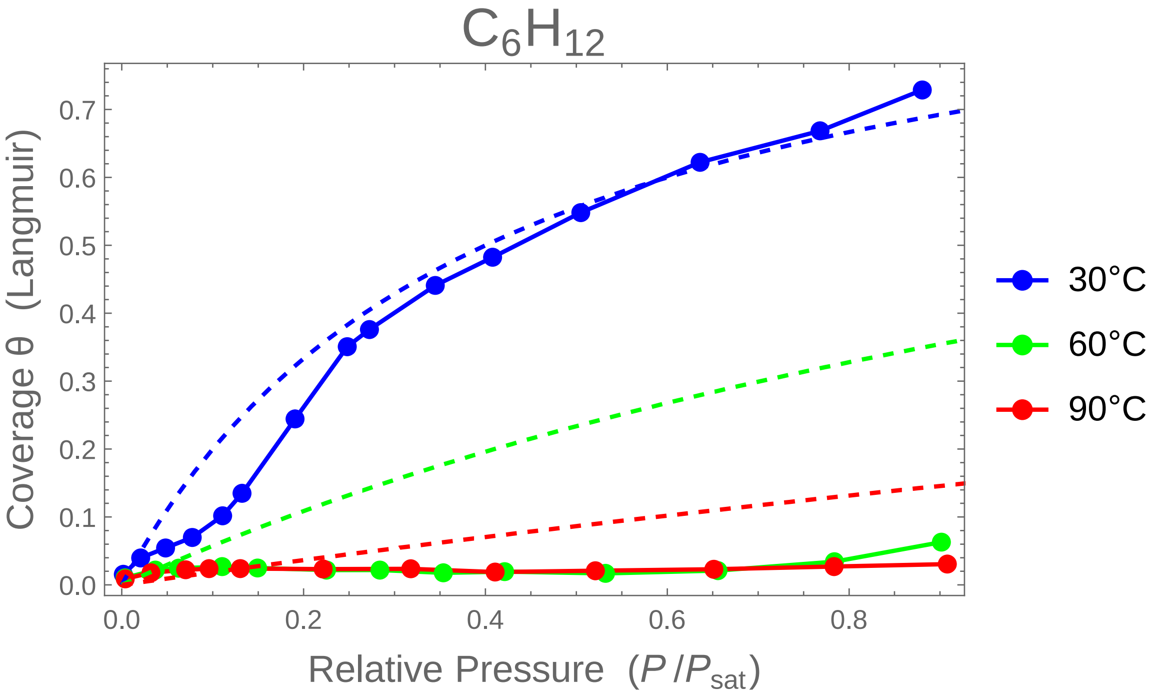

Supplementary Fig. 78: Experimental (solid-dotted) and simulated (dashed) isotherms for C_6H_{12} as a function of temperature. The experimental 30 °C uptake for C_6H_{10} is well captured by a standard Langmuir isotherm starting at relative pressures of 0.25. Going from 30 °C to 60 °C, integrating Van't Hoff's equation predicts that the thermodynamic equilibrium constant changes by a factor of 4.1; from 30 °C to 90 °C by a factor of 13.2.

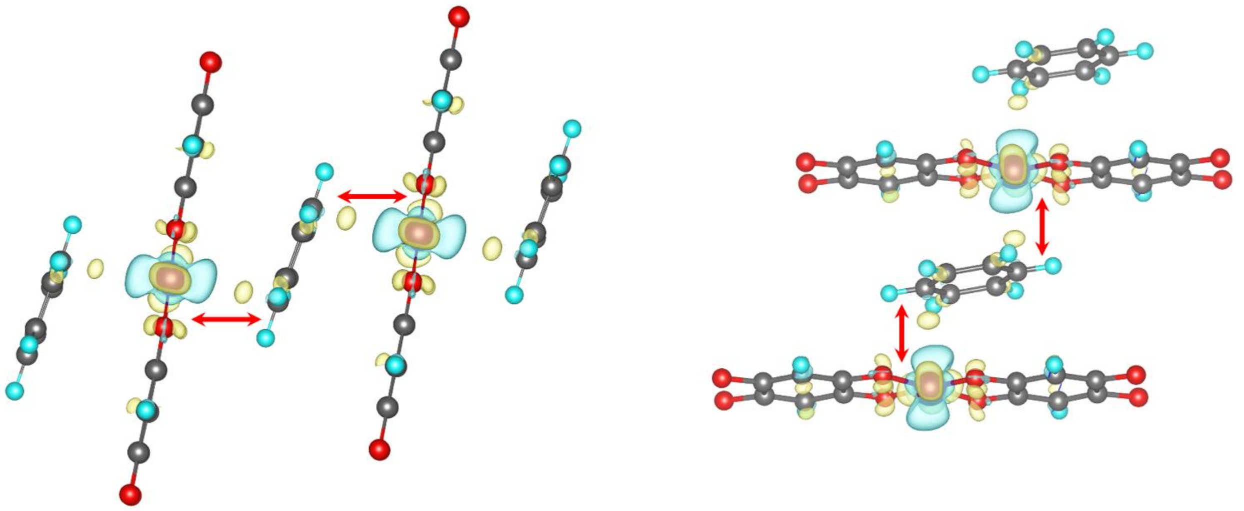

Supplementary Fig. 79. Isosurfaces of the induced charge density for the interactions of benzene molecules in Mn-DHBQ (The isovalue is 0.001 e/Å^3, and arrows are added to guide the highlight).

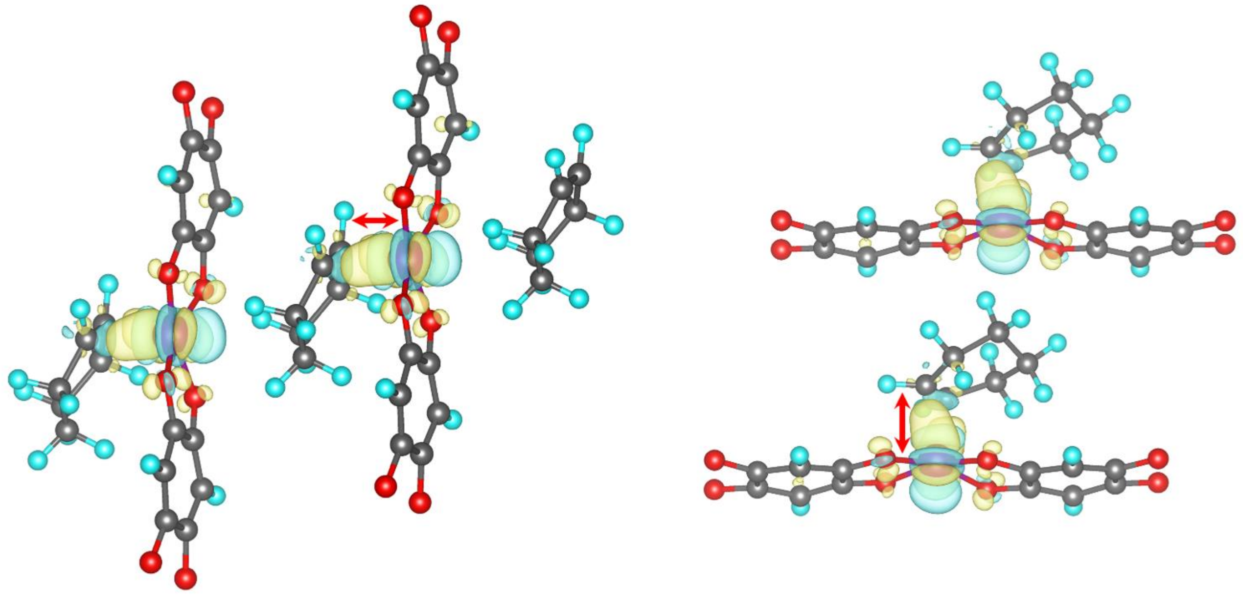

Supplementary Fig. 80. Isosurfaces of the induced charge density for the interactions of cyclohexene molecules in Mn-DHBQ (The isovalue is 0.001 e/Å^3, and arrows are added to guide the highlight).

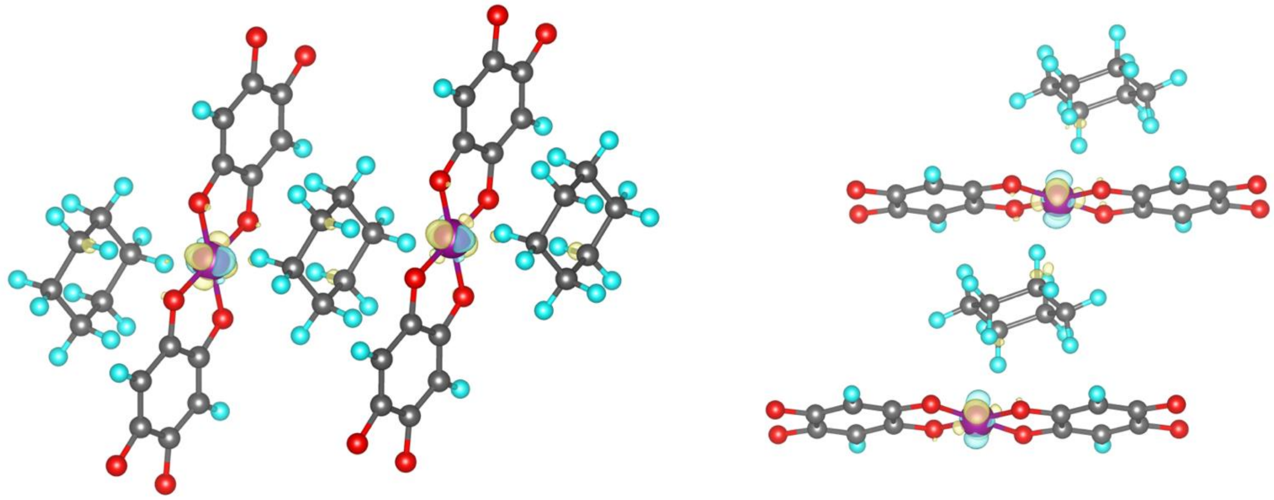

Supplementary Fig. 81. Isosurfaces of the induced charge density for the interactions of benzene molecules in Mn-DHBQ (The isovalue is 0.001 e/Å^3).

Supplementary Tables

Supplementary Table 1. Physical properties of benzene, cyclohexene, cyclohexane.

Property	Benzene	Cyclohexene	Cyclohexane
Chemical formula	C_6H_6	C_6H_{10}	C_6H_{12}
Molecular weight, g mol^{-1}	78.11	82.14	84.16
Boiling point, °C	80.10	82.98	80.74
Melting point, °C	5.53	-103.5	6.47
Dipole moment, $\times 10^{-10}$ esu Å	0	0.33	0
Kinetic diameter,[1,2] Å	5.85	~5.85	6.0
Molecular dimensions (x/y/z),[3] Å	6.628 x 7.337 x 3.277	6.973 x 6.560 x 5.020	7.168 x 6.580 x 4.982

Supplementary Table 2. A, B, C values of benzene, cyclohexene, and cyclohexane.[4,5]

Compound	A	B	C
Benzene (C_6H_6)	4.0181	1203.8	-53.23
Cyclohexene (C_6H_{10})	3.9973	1221.9	-49.98
Cyclohexane (C_6H_{12})	3.9699	1203.5	-50.29

Supplementary Table 3. ^{1}H NMR parameters of benzene, cyclohexene, and cyclohexane.

Compound	Shift (ppm)		
Benzene (C_6H_6)		7.339	
Cyclohexene (C_6H_{10})	5.664 (A)	1.991 (B)	1.612 (C)
Cyclohexane (C_6H_{12})		1.429	

Supplementary Table 4. Summary of vibrational modes of Mn-DHBQ in different forms and free ligand.

Assignment	**Frequency Position** (cm^{-1})			
	DHBQ (solid)	As made Mn-DHBQ	Activated Mn-DHBQ	C_6H_6-loaded Mn-DHBQ
ν(C-OH)	3293			
ν(OH) of interchain H_2O		3313		
β(H_2O)		1640		
ν(C=O)	1620	1600-1550	1590	1515
ν(C=C)	Obscured	1597	1615	1609
ν(C=O)/ν(C=C)		1550-1450	1550-1450	1550-1450
ν(C=C)/ν(C-O)	1375	1394	1371	1371
ν(C-OH)	1200			
ν(C-OMn)		1265	1252	1252
$\delta(CH)_{oop}$	816	816	840	856

Supplementary Table 5. Binding energies and diffusion barriers calculated for the adsorption of benzene, cyclohexene, cyclohexane on Mn-DHBQ.

**Molecule**	**Binding Energy eV (kJ mol^{-1})**	**Diffusion Barrier eV**
Benzene (C_6H_6)	0.57 (55.0)	0.08
Cyclohexene (C_6H_{10})	0.47 (45.3)	0.14
Cyclohexane (C_6H_{12})	0.16 (15.5)	0.29

Supplementary Table 6. Summary of benzene-cyclohexene-cyclohexane adsorption and separation performance of state-of-the-art adsorbents.

Adsorbent	Uptake [a] (mmol g^{-1})			Temp. (K)	Uptake Ratio (Vapor Phase)			Selectivity [b]				Ref.
	C_6H_6	C_6H_{10}	C_6H_{12}		C_6H_6/C_6H_{12}	C_6H_6/C_6H_{10}	C_6H_{10}/C_6H_{12}	C_6H_6/C_6H_{12}	C_6H_6/C_6H_{10}	C_6H_{10}/C_6H_{12}	$C_6H_6/C_6H_{10}/C_6H_{12}$	
Mn-DHBQ	2.9	2.8	1.4	303	2.1	1.1	2	97.7[c]	19.7[c]	9.2[c]	387.7/7.8/1[c]	This work
	2.7	2.0	0.08	333	33.8	1.35	25	120.1[c]	44.8[c]	16.1[c]	642.7/11.3/1[c]	
	2.6	0.15 (at 0.3 P/P_{sat})	0.02	363	130	17.3	7.5	--	--	--	--	
Zn-TCNQ-bpy	3.6	--	0.9	298	4.0	--	--	--	--	--	--	6
Mn-TCNQ-bpy	3.7	--	1.7	298	2.2	--	--	--	--	--	--	7
MAF-2	8.5	--	0.5	298	17	--	--	--	--	--	--	8
Ni-ina-bdc	2.8	--	0.1	298	28	--	--	--	--	--	--	9
CuI-BTTP4	2.6	--	1.2	298	2.2	--	--	--	--	--	--	10
CID-23	1.2	--	0.1	298	12	--	--	--	--	--	--	11
ZnL	1.4	--	0.5	298	2.8	--	--	--	--	--	--	12
$Zn(BCbpy)(PMA)_{0.5}$	0.67	--	0.10	298	6.7	--	--	--	--	--	--	13
$Zn(BCbpe)(PMA)_{0.5}$	1.38	--	0.18	298	7.6	--	--	--	--	--	--	
DAT-MOF-1	1.1	--	0.2	298	5.5	--	--	--	--	--	--	14
LiZn-bdc-NO_2-bpy	2.88	--	0.24	293	12	--	--	--	--	--	--	15
$CuL_2(NO_3)_2$	1.7	--	0.1	298	17	--	--	--	--	--	--	16
CUB-5	7.8	--	6.0	298	1.3	--	--	--	--	--	--	17
UiO-66	2.7	--	2.0	298	1.35	--	--	3[d]	--	--	--	18
UiO-66-Cu^{II}	6.0	--	4.0	298	1.5	--	--	31[d]	--	--	--	
MFM-300(Sc)	5.0	--	3.0	298	1.67	--	--	166[d]	--	--	--	
MFM-300(Cr)	4.1	--	2.3	298	1.78	--	--	103[d]	--	--	--	
MFM-300(Fe)	3.9	--	2.3	298	1.7	--	--	75[d]	--	--	--	
MAF-stu-13	2.5	--	0.2	298	12.5	--	--	138[e]	--	--	--	19
$Zn_4(EGO_2)_2(tdc)_2(dabco)$	0.95	--	0.09	298	10	--	--	77	--	--	--	20
$Zn_4(PrO_2)_2(tdc)_2(dabco)$	1.15	--	0.24	298	4.8	--	--	7	--	--	--	
ZnL'	1.51	--	0.24	298	6.3	--	--	20	--	--	--	21
$Cu(bpp)_2(BF_4)_2$	3.18	--	0.05	298	63	--	--	--	--	--	--	22
Mn-MOF-74	8.5	--	0.23	298	37	--	--	--	--	--	--	23
ZU-61	--	6.1	4.1	298	--	--	1.48	--	--	--	--	24
SIFSIX-1-Cu	--	5.9	5.5	298	--	--	1.07	--	--	--	--	
SIFSIX-2-Cu-i	--	1.1	1.05	298	--	--	1.05	--	--	--	--	
ZSM-5	--	1.3	0.7	298	--	--	1.85	--	--	--	--	
13X	--	1.8	1.5	298	--	--	1.2	--	--	--	--	

a: single-component vapor phase adsorption at relative pressure (P/P_{sat}) of ~0.8 (benzene: saturation pressure of 12.7 kPa at 298 K, cyclohexene: saturation pressure of 12.0 kPa at 298 K, cyclohexane: saturation pressure of 13.0 kPa at 298 K); *b*: selectivity values listed are for liquid phase separation of benzene-cyclohexene-cyclohexane based on GC analysis. The values are ratios of integrated areas for individual C6. *c*: The liquid-phase competitive adsorption experiments were operated for 1 hr at designed temperatures and the collected samples were dried at 25 °C before GC analysis; *d*: liquid-phase competitive adsorptions were operated for 24 hrs and the collected samples were dried at 40 °C before NMR analysis; *e*: liquid-phase competitive adsorptions were operated for 18 hrs and the collected samples were dried at 40 °C before GC analysis.

Supplementary Note 1:

Upon deprotonation of DHBQ and formation of $Mn(DHBQ)(H_2O)_2$ compound, pronounced changes occurred to the ν(C-OH) and ν(C=O) bands which are initially centered at ~1200 and ~1620 cm^{-1} in the spectrum of DHBQ, yet appear respectively at 1265 and 1600-1450 cm^{-1} due to the formation of M-O bonds. It is worth noting that the broad intense band at 1600-1450 cm^{-1} not only contains the contribution of single C=O stretching that is located above 1550 cm^{-1} but also involves mixed C=O/C=C stretching that occurs below 1550 cm^{-1}.[25] Other characteristic DHBQ modes such as mixed ν(C-C)/ν(C-O) band occurs at ~1394 cm^{-1} and localized $\delta(CH)_{oop}$ at ~816 cm^{-1}. In addition, interchain water absorption bands including ν(OH) and $\beta(H_2O)$ are also notable at 3313 and 1640 cm^{-1}, which shift substantially as compared with that of free water molecules at above 3600 and below 1600 cm^{-1},[26] owing to the formation of strong hydrogen-bond with the oxygen of DHBQ linker.

Supplementary References

1 Li, J.-R., Kuppler, R. J. & Zhou, H.-C. Selective gas adsorption and separation in metal–organic frameworks. *Chemical Society Reviews* **38**, 1477-1504 (2009).

2 Webster, C. E., Drago, R. S. & Zerner, M. C. Molecular dimensions for adsorptives. *Journal of the American Chemical Society* **120**, 5509-5516 (1998).

3 Liu, C.-H. *et al.* A B←N framework based on 1D dative B←N polymers for exclusive recognition and separation of benzene from its azeotrope. *Chem* (2023).

4 Willingham, C. B., Taylor, W. J., Pignocco, J. M. & Rossini, F. D. Vapor pressures and boiling points of some paraffin, alkylcyclopentane, alkylcyclohexane, and alkylbenzene hydrocarbons. *Journal of Research of the National Bureau of Standards* **35**, 219-244 (1945).

5 Meyer, E. F. & Hotz, R. D. High-precision vapor-pressure data for eight organic compounds. *Journal of Chemical and Engineering Data* **18**, 359-362 (1973).

6 Shimomura, S., Horike, S., Matsuda, R. & Kitagawa, S. Guest-specific function of a flexible undulating channel in a 7, 7, 8, 8-tetracyano-p-quinodimethane dimer-based porous coordination polymer. *Journal of the American Chemical Society* **129**, 10990-10991 (2007).

7 Shimomura, S., Matsuda, R. & Kitagawa, S. Flexibility of porous coordination polymers strongly linked to selective sorption mechanism. *Chemistry of Materials* **22**, 4129-4131 (2010).

8 Zhang, J.-P. & Chen, X.-M. Exceptional framework flexibility and sorption behavior of a multifunctional porous cuprous triazolate framework. *Journal of the American Chemical Society* **130**, 6010-6017 (2008).

9 Ren, G. *et al.* A 9-connected metal–organic framework with gas adsorption properties. *Journal of Materials Chemistry* **21**, 15909-15913 (2011).

10 Yang, R. *et al.* Two Robust Porous Metal–Organic Frameworks Sustained by Distinct Catenation: Selective Gas Sorption and Single‐Crystal‐to‐Single‐Crystal Guest Exchange. *Chemistry–An Asian Journal* **5**, 2358-2368 (2010).

11 Hijikata, Y. *et al.* Relationship between channel and sorption properties in coordination polymers with interdigitated structures. *Chemistry–A European Journal* **17**, 5138-5144 (2011).

12 Joarder, B. *et al.* Guest‐Responsive Function of a Dynamic Metal–Organic Framework with a π Lewis Acidic Pore Surface. *Chemistry–A European Journal* **20**, 15303-15308 (2014).

13 Ren, C.-X. *et al.* π-Conjugation-directed highly selective adsorption of benzene over cyclohexane. *Journal of Materials Chemistry A* **2**, 9015-9019 (2014).

14 Manna, B. *et al.* A π-electron deficient diaminotriazine functionalized MOF for selective sorption of benzene over cyclohexane. *Chemical Communications* **51**, 15386-15389 (2015).

15 Sapianik, A. A. *et al.* Exceptionally effective benzene/cyclohexane separation using a nitro-decorated metal–organic framework. *Chemical Communications* **56**, 8241-8244 (2020).

16 Karmakar, A., Desai, A. V., Manna, B., Joarder, B. & Ghosh, S. K. An Amide‐Functionalized Dynamic Metal–Organic Framework Exhibiting Visual Colorimetric Anion Exchange and Selective Uptake of Benzene over Cyclohexane. *Chemistry–A European Journal* **21**, 7071-7076 (2015).

17 Macreadie, L. K. *et al.* CUB-5: a contoured aliphatic pore environment in a cubic framework with potential for benzene separation applications. *Journal of the American Chemical Society* **141**, 3828-3832 (2019).

18 Han, Y. *et al.* Control of the pore chemistry in metal-organic frameworks for efficient adsorption of benzene and separation of benzene/cyclohexane. *Chem* **9**, 739-754 (2023).

19 Ye, C. R. *et al.* Harnessing Shape Complementarity for Upgraded Cyclohexane Purification through Adaptive Bottlenecked Pores in an Imidazole‐Containing MOF. *Angewandte Chemie International Edition* **60**, 23590-23595 (2021).
20 Lysova, A. A. *et al.* Tuning the molecular and cationic affinity in a series of multifunctional metal–organic frameworks based on dodecanuclear Zn (II) carboxylate wheels. *Journal of the American Chemical Society* **141**, 17260-17269 (2019).
21 Li, G., Zhu, C., Xi, X. & Cui, Y. Selective binding and removal of organic molecules in a flexible polymeric material with stretchable metallosalen chains. *Chemical Communications*, 2118-2120 (2009).
22 Kondo, A., Suzuki, T., Kotani, R. & Maeda, K. Liquid/vapor-induced reversible dynamic structural transformation of a three-dimensional Cu-based MOF to a one-dimensional MOF showing gate adsorption. *Dalton Transactions* **46**, 6762-6768 (2017).
23 Mukherjee, S. *et al.* Harnessing Lewis acidic open metal sites of metal–organic frameworks: the foremost route to achieve highly selective benzene sorption over cyclohexane. *Chemical Communications* **52**, 8215-8218 (2016).
24 Chen, S. *et al.* Anion-pillared porous materials with suitable pore size for the efficient discrimination of cyclohexene from cyclohexane. *Separation and Purification Technology* **302**, 122095 (2022).
25 Mostafa, S. I. Complexes of 2, 5-dihydroxy-1, 4-benzoquinone and chloranilic acid with second and third row transition elements. *Transition Metal Chemistry* **24**, 306-310 (1999).
26 Tan, K. *et al.* Water reaction mechanism in metal organic frameworks with coordinatively unsaturated metal ions: MOF-74. *Chemistry of Materials* **26**, 6886-6895 (2014).
